# Deciphering m^6^A methylation in monocyte-mediated cardiac fibrosis and monocyte-hitchhiked erythrocyte microvesicle biohybrid therapy

**DOI:** 10.7150/thno.95664

**Published:** 2024-06-01

**Authors:** Jiawen Li, Li Wei, Kaifeng Hu, Yunxiang He, Guidong Gong, Qisong Liu, Yue Zhang, Kaiyu Zhou, Junling Guo, Yimin Hua, Jun Tang, Yifei Li

**Affiliations:** 1 Key Laboratory of Birth Defects and Related Diseases of Women and Children of MOE, Department of Pediatrics, West China Second University Hospital, Sichuan University, Chengdu, Sichuan 610041, China.; 2 Key Laboratory of Birth Defects and Related Diseases of Women and Children of MOE, Laboratory of Genetic Disease and Perinatal Medicine, Sichuan University, Chengdu, Sichuan 610041, China.; 3 BMI Center for Biomass Materials and Nanointerfaces, College of Biomass Science and Engineering, Sichuan University, Chengdu, Sichuan 610065, China.; 4 Department of Chemical and Biological Engineering, The University of British Columbia, Vancouver, BC V6T 1Z4, Canada.

**Keywords:** m^6^A modification, monocytes, cardiac fibrosis, erythrocyte microvesicles, meta-phenolic network

## Abstract

**Rationale:** Device implantation frequently triggers cardiac remodeling and fibrosis, with monocyte-driven inflammatory responses precipitating arrhythmias. This study investigates the role of m^6^A modification enzymes METTL3 and METTL14 in these responses and explores a novel therapeutic strategy targeting these modifications to mitigate cardiac remodeling and fibrosis.

**Methods:** Peripheral blood mononuclear cells (PBMCs) were collected from patients with ventricular septal defects (VSD) who developed conduction blocks post-occluder implantation. The expression of METTL3 and METTL14 in PBMCs was measured. METTL3 and METTL14 deficiencies were induced to evaluate their effect on angiotensin II (Ang II)-induced myocardial inflammation and fibrosis. m^6^A modifications were analyzed using methylated RNA immunoprecipitation followed by quantitative PCR. NF-κB pathway activity and levels of monocyte migration and fibrogenesis markers (CXCR2 and TGF-β1) were assessed. An erythrocyte microvesicle-based nanomedicine delivery system was developed to target activated monocytes, utilizing the METTL3 inhibitor STM2457. Cardiac function was evaluated via echocardiography.

**Results:** Significant upregulation of METTL3 and METTL14 was observed in PBMCs from patients with VSD occluder implantation-associated persistent conduction block. Deficiencies in METTL3 and METTL14 significantly reduced Ang II-induced myocardial inflammation and fibrosis by decreasing m^6^A modification on *MyD88* and *TGF-β1* mRNAs. This disruption reduced NF-κB pathway activation, lowered CXCR2 and TGF-β1 levels, attenuated monocyte migration and fibrogenesis, and alleviated cardiac remodeling. The erythrocyte microvesicle-based nanomedicine delivery system effectively targeted inflamed cardiac tissue, reducing inflammation and fibrosis and improving cardiac function.

**Conclusion:** Inhibiting METTL3 and METTL14 in monocytes disrupts the NF-κB feedback loop, decreases monocyte migration and fibrogenesis, and improves cardiac function. Targeting m^6^A modifications of monocytes with STM2457, delivered via erythrocyte microvesicles, reduces inflammation and fibrosis, offering a promising therapeutic strategy for cardiac remodeling associated with device implantation.

## Introduction

Cardiac remodeling and fibrosis following cardiac device implantation is a multifaceted process central to the development of severe complications, including arrhythmia and heart failure 1,2. This myocardial injury and pathological cardiac remodeling are characterized by a cascade of cellular events initiated by the infiltration and activation of monocytes and macrophages 3,4. The highly activated monocytes produce more aggressive inflammation and responding fibrosis secondary to device implantation.

Despite the acknowledged importance of monocytes in cardiac remodeling, the specific molecular mechanisms underpinning their behavior, particularly concerning epigenetic modifications, have not been thoroughly explored 5. Above all, m^6^A RNA methylation has emerged as a crucial post-transcriptional regulator that influences various cellular processes and has been considered a potential therapeutic target 6,7. Recent studies reveal that m^6^A methylation is vital in maintaining and shaping the function of monocytes and macrophages, proven to be involved in inflammation regulation of atherosclerosis 8,9, immune responses to bacterial infections 10 and cytokine production in endotoxemia 11. However, the exact role of m^6^A modification in monocyte-derived macrophage differentiation during cardiac remodeling remains unclear 12. Deepening insights into the influence of m^6^A RNA methylation on monocyte and macrophage behavior in heart disease is critical to developing strategies that reduce myocardial fibrosis post-device implantation.

**Figure 1:**
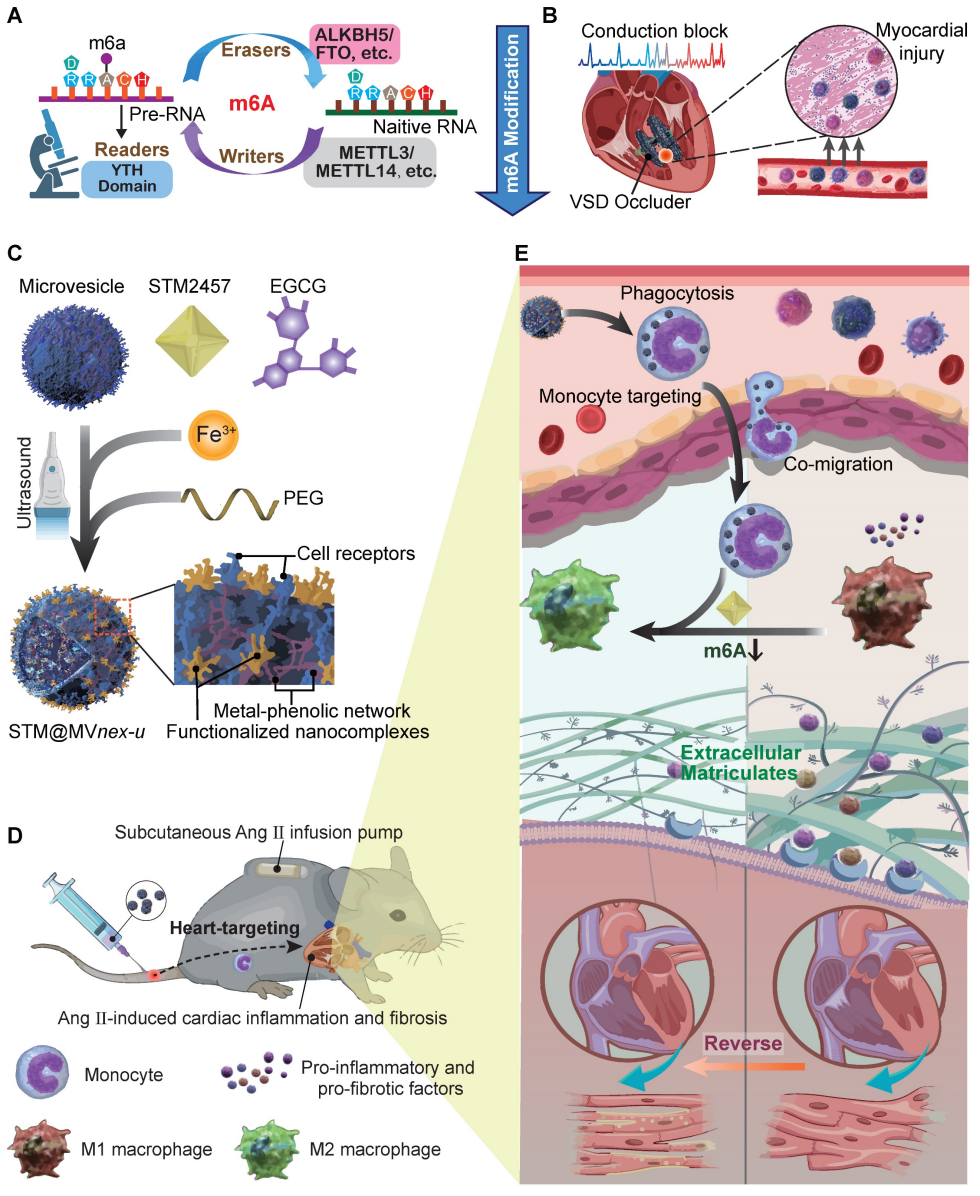
** Schematic of general mechanisms and therapeutic strategies for modulating monocyte m^6^A modification in cardiac fibrosis and remodeling processes.** (**A**) Standard regulation model of m^6^A modification involving writers, erasers, and readers (WERs). (**B**) Local infiltration of leukocytes, mainly monocytes, following occluder implantation for VSD interventional closure. (**C**) Three-step assembly of the erythrocyte microvesicle biohybrid system. (**D**) Subcutaneous implantation of an Ang II minipump in a mouse model of cardiac remodeling, with intravenous injection of erythrocyte MVs carrying STM2457, hitchhiked by monocytes to the targeted heart. (**E**) Monocytes that phagocytose engineered drug-loaded MVs in the blood infiltrate the fibrotic and remodeling cardiac tissue, influencing the differentiation and function of monocyte to macrophage subtypes, thereby improving cardiac function and prognosis.

To explore this issue, we recruit patients undergoing ventricular septal defect (VSD) device closure, aiming to reveal m^6^A involvement in VSD occluder implantation-related conduction block (CB) primarily triggered by local fibrosis. Our investigation indicates that in monocytes of peripheral blood mononuclear cells (PBMCs) from patients or monocytes upon lipopolysaccharide (LPS) stimulation *in vitro*, there is an upregulation of the m^6^A modification enzymes of methyltransferase-like 3 and 14 (METTL3 and METTL14) 13, suggesting their potential association with the inflammatory responses of monocytes/macrophages in cardiac fibrosis secondary to VSD occluder implantation (**Figure 1A**). Furthermore, we explore the consequences of METTL3 and METTL14 deficiency in these cells, determining their molecular contributions to chemotactic migration, inflammation, and fibrogenic activities 8,14,15 (**Figure S1**), which are considered the essential factors involved in VSD occluder implantation-associated cardiac fibrosis 16-18.

After fully understanding the molecular mechanisms of m^6^A in cardiac fibrosis, we introduce an innovative biohybrid delivery system for the METTL3 inhibitor STM2457 19, utilizing erythrocyte-derived microvesicles (MVs) to target m^6^A modifications in activated monocytes (**Figure 1B**) 20-27. Erythrocyte-derived MVs are extracellular vesicles of submicron dimensions, and elevated intracellular calcium levels and oxidative stress predominantly initiate its formation. This process facilitates the expulsion of damaged hemoglobin (Hb), thereby contributing to the extended lifespan of erythrocytes 28,29. This method capitalizes on monocytes' natural phagocytic tendencies towards oxidative stress-induced erythrocyte MVs 30,31, providing insights into m^6^A modification in monocytes and offering a potential breakthrough in cardiac immunotherapy (**Figure 1C**-**D**). Distinct from MVs derived from immortalized cells, erythrocyte MVs are advantageous due to the lack of nuclear, mitochondrial DNA, and retrotransposon elements, thereby reducing extraneous influences and interferences in mechanism exploration and risk of oncogenesis 32. This feature facilitates using bio-nanotechnology to validate biomolecular mechanisms through non-genetic editing approaches 33,34. Through this integrative approach with metal-phenolic network (MPN), combining molecular insights with novel therapeutic strategies, our study aims to provide a comprehensive understanding of the molecular role of m^6^A in monocyte-mediated cardiac fibrosis and introduce new avenues of the biohybrid therapeutic approach for VSD occluder implantation-related complications and heart diseases 35,36.

## Results

### m^6^A hypermethylation aggravates VSD occluder implantation injuries

A cohort of pediatric patients received transcatheter VSD device occlusion in our center. The most prevalent complication, post-VSD occluder implantation-associated CB, had been carefully observed. Postoperative arrhythmic monitoring is conducted over the first seven days. Patients with CB after this period enter prolonged observation, with a follow-up arrhythmia assessment scheduled at approximately three months. Those without arrhythmias or with rapid CB resolution within the initial seven days are classified as the non-CB group (NCB). Patients whose CB persists initially but resolves by the three-month are termed the transient CB group (TCB). Those still exhibiting CB at three months are defined as the persistent CB group (PCB).

We analyzed CB's prevalence and prognostic factors in 97 patients, as detailed in our datasets. The distributions of transient and persistent CB, such as left/right bundle branch block, left anterior fascicular block and atrioventricular block were outlined (**Table S1**). Risk factors influencing the prognosis, including demographic, electrocardiographic, and anatomical variables, are evaluated (**Table S2** and **S3**).

**Figure 2:**
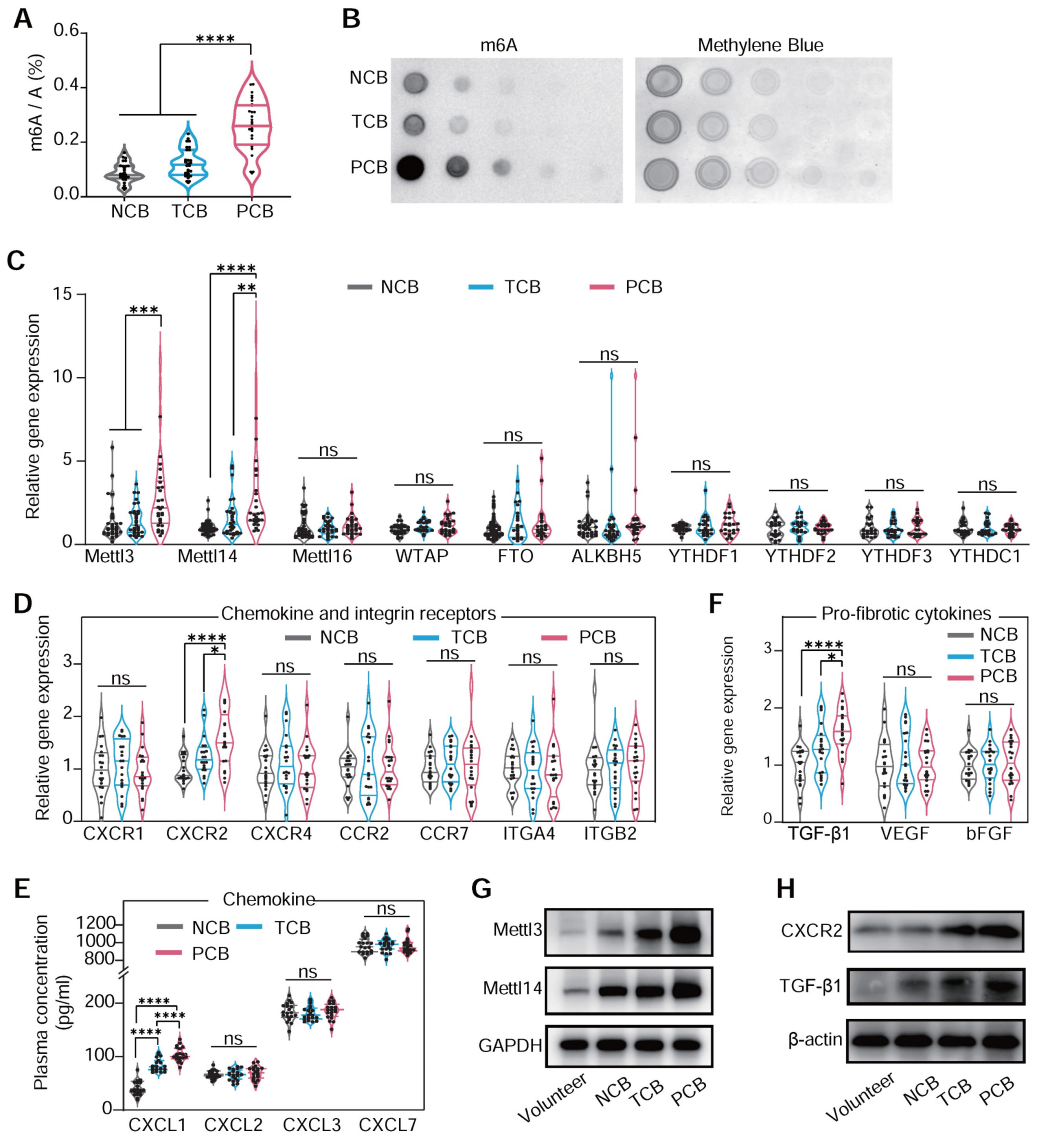
** m^6^A hypermethylation aggravates cardiac VSD occluder implantation injuries.** (**A** and **B**) The RNA m^6^A methylation levels in the PBMCs of patients with either transient or persistent conduction block following VSD occluder implantation and those from patients without postoperative arrhythmias. Global m^6^A level of PBMC mRNA was detected by colorimetric (A) and (B) dot blot. (**C**) mRNA levels of m6a WER genes, including METTL3, METTL14, METTL16, WTAP, FTO, ALKBH5, YTHDF1, YTHDF2, YTHDF3 and YTHDC1 in patients. (**D**) mRNA expression of chemokine receptors of PBMC from patients, including CXCR1, CXCR2, CXCR4, CCR2, CCR7, ITGA4 and ITGB2. (**E**) The cluster of chemokine concentration in the plasma of patients, including CXCL1, CXCL2, CXCL3 and CXCL7. (**F**) mRNA expression of pro-fibrotic molecules of TGF-β1, VEGF and bFGF. (**G**) Western blot for protein level of METTL3/METTL14 in PBMCs from healthy volunteers (control), or patients with or without conduction block after occluder implantation. (**H**) Western blot for protein level of CXCR2 and TGF-β1 in PBMCs. For (A) and (C-F), n≥18 per group. The data are expressed as the mean ± SD. P-values were determined by one-way ANOVA with Fisher's LSD post-hoc test. *, P < 0.05; **, P < 0.01; ***, P < 0.001; ****, P < 0.0001.

Significant predictors of persistent blocks include RV1 voltage and male gender (**Table S2**), while multivariable analysis indicated limited predictive power for most variables, except for a notable association in the likelihood of persistent block with left ventricle enlargement (**Table S3**). This structured approach provides valuable insights into the factors that may influence the clinical management of CB. In previous research, device-related arrhythmia was induced by localized inflammation and aggressive myocardial fibrosis 37. Our analysis of PBMCs mRNA from patients of occluder implantation for VSD closure showed a significantly elevated m^6^A level in the PCB group compared to NCB and TCB patients, as determined by dot blot and colorimetric assays (**Figure 2A**-**B**). This suggested that m^6^A modification in monocytes might be crucial in VSD occluder implantation-induced cardiac inflammation and fibrosis. Generally, m^6^A modification is primarily mediated by three parts of components: writers, erasers, and readers, collectively known as WER proteins 6. To validate our hypothesis and identify the potential factor driving the responding pathological changes of m^6^A modification during myocardial fibrosis, we analyzed various m^6^A WER genes. This led to the discovery of a significantly increased expression of *METLL3* and *METLL14* in the PCB group (**Figure 2C**).

To understand monocyte migration from circulation to injured myocardium, we investigated the expression of crucial chemokine receptors in PBMCs associated with monocyte movement and infiltration 18,38. Our examination included a range of well-recognized chemokine receptors 39 and observed a marked increase in CXCR2 mRNA in PCB patients, whereas CXCR1, CXCR4, CCR2, and CCR7 expression levels remained consistent among three groups. Adhesion molecules ITGA4 and ITGB2 also showed no significant change among the groups (**Figure 2D**) 40. This alternation coincided with the change of plasma level CXCL1, a ligand that binds to CXCR2, ranking the top upregulation in patients of PCB (**Figure 2E**). Regarding pro-fibrotic molecules 41, TGF-β1 protein was significantly upregulated in the PCB group (**Figure 2F**). Aligning with the trends observed in mRNA expression, METLL3, METLL14, CXCR2, and TGF-β1 protein levels were notably higher in the PCB group compared to the healthy volunteers and patients in NCB /TCB groups. (**Figure 2G**-**H**). Further analysis of cytokine mRNA in the PCB group showed increased IL-10, IL-6, and TNF-α levels (**Figure S2**). This finding demonstrated the significant activation and involvement in the inflammatory processes of monocytes in patients with VSD occluder implantation-associated CB.

Our study illuminates the critical interplay between myocardial injuries, monocyte activity, and m^6^A modifications in pediatric patients with occluder implantation for VSD. It revealed the potential factors collectively involved in the development of postoperative myocardial injuries, providing a promising target in VSD occluder implantation-related complications management.

### METTL3/METTL14 activates monocytes participating in cardiac fibrosis

Using single-cell RNA sequencing, we evaluated the enrichment of METTL3 and METTL14 in human and mouse heart cells, focusing on their enrichment in cardiac macrophages. Enrichment prediction scores across various cardiac cell types underscored distinct roles for these genes, especially in macrophages where t-SNE and UMAP analyses demonstrated relatively high expression levels in humans and mice (**Figure S3A**-**B**). This suggests a crucial regulatory impact of METTL3 and METTL14 on macrophage transcriptomes. Both GO-Term and Gene Set Enrichment Analyses (GSEA) highlighted substantial activation of pathways primarily related to cytokine production in macrophages after Ang II induction (**Figure S3C**-**E**). Additionally, AUCell analysis confirmed increased activity in pathways associated with producing pro-inflammatory cytokines, emphasizing the crucial roles of METTL3 and METTL14 in modulating inflammatory responses in cardiac macrophages under chronic stress conditions (**Figure S3F**).

To investigate the role of METTL3/METTL14-mediated m^6^A modification in regulating the function of monocytes/macrophages, we inhibited METTL3 and METTL4 expression by siRNAs transfection in RAW264.7 cells with high efficacy (**Figure S4A**-**D**). Based on the site-specific siRNA identified with the highest knockdown efficiency in the RAW264.7 cell line, we verified the chosen siRNA exhibits a notable efficiency in primary bone marrow-derived monocytes (BMDMs). (**Figure S4E**-**F**). We employed clodronate liposomes to deplete endogenous macrophages and monocytes in mouse models with Ang II-induced cardiac fibrosis and remodeling 42. Monocyte counts in the blood increase post-Ang II induction and decrease markedly after a 5-day treatment with clodronate liposomes. Other white blood cell populations remain stable, showing minimal response to these interventions (**Figure S5**). Ang II induction significantly increases the number of cardiac macrophages in mice, primarily those derived from circulating monocytes, indicating an inflammatory cardiac state under chronic stress. Five days post intraperitoneal administration of clodronate liposomes, nearly all resident cardiac macrophages are depleted, along with a marked reduction in macrophages differentiated from circulating monocytes (**Figure S6**). Subsequently, we externally injected wild-type (WT) BMDMs pre-treated with PBS (BMDMs^WT^), LPS (BMDMs^LPS^), or LPS combined with siRNA inhibiting METTL3 and METTL14 (BMDMs^LPS+siM3&14^).

**Figure 3:**
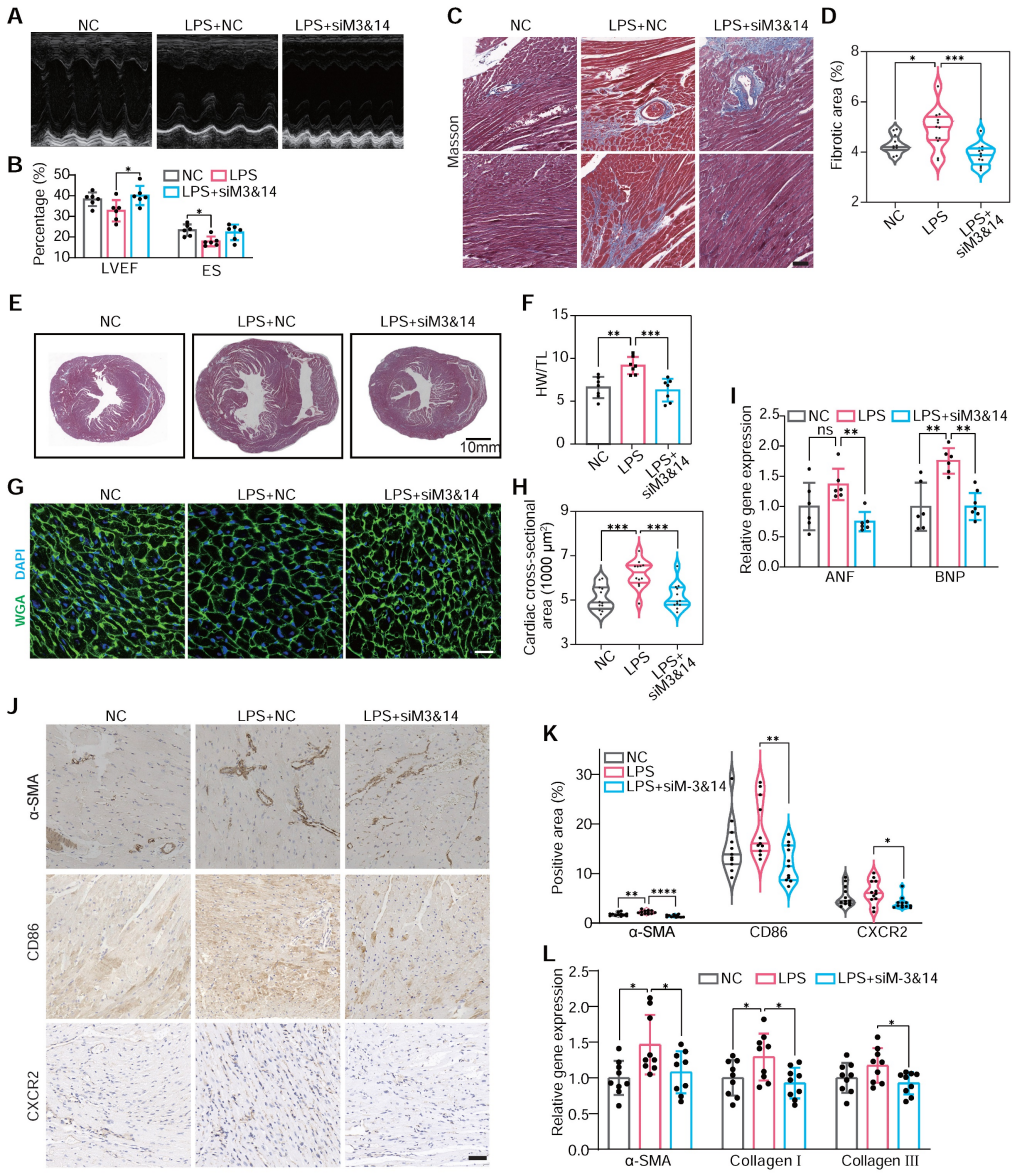
** METTL3/METTL14 activates monocytes that participate in cardiac fibrosis.** (**A** and **B**) M-mode echocardiography (A) of the left ventricular chamber and measurement of ejection fraction (EF%) and fractional shortening (FS%) (B). n = 6 per group. (**C** and **D**) Masson's trichrome staining of myocardial fibrosis (C) and quantification of fibrotic area (D). Scale bars, 50 μm. n = 12 per group from at least 3 mice. (**E**) Masson staining of whole heart sections. (**F**) The heart weight to tibial length (HW/TL) ratio. n = 7 per group. (**G** and **H**) FITC-labeled wheat germ agglutinin staining of heart sections (g) and quantification of cardiomyocytes cross-sectional area (about 200 cells counted per heart). Scale bars, 25 μm. n = 12 per group. (**I**) qRT-PCR analysis of the mRNA levels of atrial natriuretic factor (ANF) and brain natriuretic peptide (BNP) in the hearts. n = 6 per group. (**J** and **K**) Immunohistochemical staining of α-SMA, CD86 and CXCR2 in the hearts (j) and the percentage of positive areas (K). Scale bars, 50 μm. n = 11 per group from at least 3 mice. (**L**) qRT-PCR analysis of the mRNA levels of α-SMA, collagen I, and collagen III in the hearts. n = 9 per group. WGA, wheat germ agglutinin. For (B), (F), (I) and (L), P-values were determined by one-way ANOVA with Fisher's LSD post-hoc test. For (D), (H) and (K), statistical analyses were performed using two-tailed Mann-Whitney U-tests. All data are expressed as mean ± SD. *, P < 0.05; **, P < 0.01; ***, P < 0.001; ****, P < 0.0001.

This approach enabled us to scrutinize the specific roles of METTL3 and METTL14 in monocyte-mediated cardiac responses. Echocardiography demonstrated that BMDM^LPS+siM3&14^ improved cardiac function and mitigated hypertrophy and fibrosis compared to BMDMs^LPS^ (**Figure 3A**-**B**). Histological analysis and immunohistochemical staining echoed these findings, showing reduced cardiomyocyte size, heart weight to tibial length ratio, and markers of cardiac hypertrophy and fibrosis area in BMDM^LPS+siM3&14^ treated animals (**Figure 3C**-**I**). Western blot analysis of the left ventricle revealed that Ang II induction led to a significantly more significant increase in type I collagen than type III, causing a marked elevation in type I/type III collagen ratio (**Figure S7**). This change is indicative of reactive fibrosis, typically resulting from increased afterload or chronic cardiac disease, as opposed to replacement fibrosis often seen following acute myocardial infarction. In a mouse model where chlorate was used to deplete monocytes/macrophages, the reduction in the increased type I/type III collagen ratio caused by intravenous injection of Ang II indicated a decrease in the extent of reactive fibrosis. This effect was also pronounced in LPS-preactivated BMDMs, while those with a prior knockdown of METTL3 and METTL14 effectively reversed the LPS-induced alterations.

Further analysis revealed decreased α-SMA-positive myofibroblasts 43, CD86-positive, and CXCR2-positive cells in the hearts of mice receiving BMDMs^LPS+siM3&14^ compared to those receiving BMDMs^LPS^, suggesting a reduction in an inflammatory response and pro-fibrotic activity (**Figure 3J**-**L**). Additionally, we observed a significant decrease in monocyte recruitment to ischemic hearts in BMDM^LPS+siM3&14^ groups, confirmed by laser scanning confocal microscopy (LSCM) and flow cytometry (**Figure S8A**-**D**). qRT-PCR and protein analysis showed downregulation of M1 markers and upregulation of M2 markers of macrophages isolated from left ventricles days 10 after treatment, indicating a shift in macrophage polarization (**Figure S8E**-**F**). The immunoblot revealed a reduction in the macrophage marker F4/80 and the pro-fibrotic factor TGF-β1 within the myocardial tissue of mice receiving BMDMs^LPS+siM3&14^ compared to those receiving BMDMs^LPS^ (**Figure S8G**). NF-κB pathway analysis further substantiated the role of METTL3 and METTL14 of monocytes in activating these processes (**Figure S8H**).

These findings revealed that the m^6^A modification highly activated monocytes involved in the inflammation responses after myocardial injuries, resulting in the pathophysiological fibrotic process. However, the molecular mechanisms were still unclear.

### m^6^A modification regulates the polarization of monocyte/macrophage in mediating inflammatory responses

**Figure 4:**
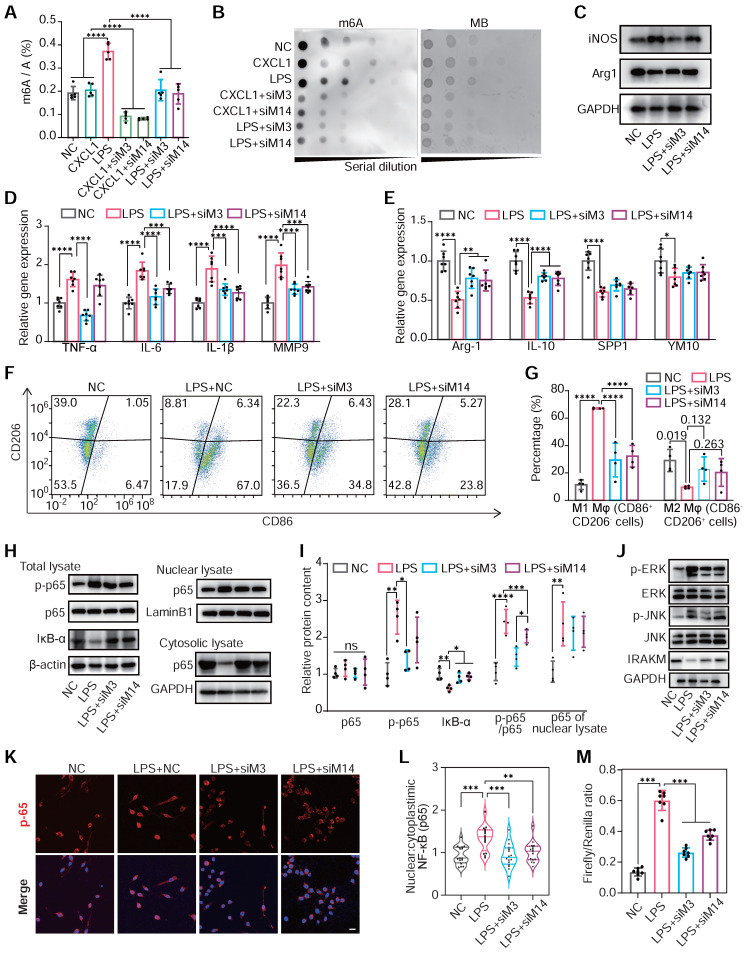
**m^6^A modification regulates the polarization of monocyte/macrophage in mediating inflammatory responses.** (**A** and **B**) The m^6^A levels of mRNA of RAW264.7 cells transfected with NC siRNA, METTL3 siRNA (siM3), METTL14 siRNA (siM14) before treatment with LPS (500 ng/mL) or CXCL1 (20 ng/mL). The global m6a levels were detected by colorimetric (A) and dot blot (B). n = 5 per group. (**C**) Protein content of iNOS (M1 marker) and Arg1 (M2 marker) in RAW264.7 cells after treatment with NC, LPS, LPS+siM3, LPS+siM14. (**D**) qRT-PCR of mRNA levels for M1 markers of TNF-α, IL-6, IL-1β and MMP9. n = 7 per group. (**E**) qRT-PCR of mRNA levels for M2 markers of Arg-1, IL-10, SPP1 and YM1. n = 7 per group. (**F** and **G**) Flow cytometry analysis and quantification of M1 polarization (CD86^+^ CD206^-^) and M2 polarization (CD86^-^ CD206^+^) of RAW264.7 cells. n = 4 per group. (**H**) Immunoblot for total lysate of IκBα, p65, p50 and phosphor-p65 (p-p65, Ser536), nuclear and cytosolic lysates of p65. (**I**) Quantification of protein contents of p-p65, p65, IκB-α and p-p65/p65 of total lysate normalized to GAPDH and p65 of nuclear lysate normalized to Lamin B1. n = 4 per group. (**J**) Western blotting of JNK, ERK, and IRAKM, which are downstream proteins of the TLR4 signaling pathway. (**K**) Immunofluorescence analysis of LPS-induced p65 nuclear translocation. Scale bar, 30 μm. (**L**) Quantification of nuclear:cytoplasmic ratios of p65 immunofluorescence staining. n ≥ 15 per group from at least three mice. (**M**) Firefly & Renilla Luciferase detection using lysates from Raw264.7 cells co-transfected with pGL3-NF-κB-Luc and pRL-CMV. n = 7 per group. For (A), (D), (E), (G), (I), (L) and (M), n ≥5 per group. The data are expressed as the mean ± SD. P-values were determined by one-way ANOVA with Fisher's LSD post-hoc test. *, P < 0.05; **, P < 0.01; ***, P < 0.001; ****, P < 0.0001.

LPS administration, not CXCL1, positively enhanced m^6^A modification levels in total mRNA. Meanwhile, the knockdown of METTL3 and METTL14 would significantly attenuate hypermethylation induced by LPS (**Figure 4A**-**B**). This indicated that m^6^A modification was closely associated with activating the TLR4/NF-κB pathway, an LPS responder 44.

Subsequently, we explored the changes in macrophage polarization of RAW264.7 cells under METTL3 and METTL14 knockdown post-LPS treatment. The knockdown of METTL3 and METTL14 reduced the expression of M1 marker genes and elevated the expression of M2 marker genes under LPS stimulation (**Figure 4C**-**E**). Flow cytometry confirmed these findings, showing reduced M1 macrophages and increased M2 populations following METTL3 or METTL14 knockdown (**Figure 4F**-**G**). Delving into the underlying mechanisms, we found that either METTL3 or METTL14 knockdown hindered LPS-induced p65 phosphorylation, nuclear translocation and IκB-α degradation, thereby attenuating TLR4 pathway activation (**Figure 4H**-**M**). Accordingly, the overexpression of METTL3 and METTL14 resulted in an increased global m^6^A level (**Figure S9A**-**D**), activated the NF-κB pathway, and enhanced M1 polarization in response to LPS by assessing the improved expression of TNF-α and IL-6 (**Figure S9E**-**F**). To identify macrophage mRNA transcripts whose expressions are affected by m^6^A modification, we profiled the m^6^A epitranscriptome of WT and METTL3-knockout (KO) macrophages at baseline or under LPS stimulation. Then, we correlated the m^6^A methylation profiles through integrated analyses of these two datasets. m^6^A peak distribution on different transcript segments showed significant responses upon METTL3 KO under LPS intervention. (**Figure S10A**-**B**) The downregulated METTL3-KO-responsive m^6^A peaks are clustered in immune and inflammatory reactions in Gene Ontology analysis and enriched specifically in NF-kB and TLR4 signaling pathways in Kyoto Encyclopedia of Genes and Genomes pathway analysis (**Figure S10C**-**G**).

Our findings emphasized that METTL3/METTL14-mediated m^6^A modification was vital in influencing macrophage polarization between M1 and M2 phenotypes. This modulation was supposed to be regulated by the TLR4/NF-κB signaling pathway, indicating an association between m^6^A modification and inflammatory response of monocytes/macrophages.

### m^6^A hypermethylation elevates cellular migration of monocyte/macrophage

To further substantiate our study on the role of m^6^A methylation in monocyte-mediated migration and fibrosis, we examined the m^6^A methylation on the *TGF-β1* transcript in WT macrophages following LPS treatment. Read density analysis revealed a significant increase in m^6^A methylation after LPS treatment, an effect substantially reduced in METTL3-KO macrophages (**Figure S10H**). In contrast, we did not observe this methylation pattern on the CXCR2 mRNA. Additionally, increased expression of CXCR2 and TGF-β1 was documented in patient-derived PBMCs, indicating a nuanced regulatory mechanism of m^6^A modification in these transcripts.

The overexpression of METTL3 and METTL14 increased the m^6^A modification and elevated TGF-β1 expression in RAW264.7 cells (**Figure S11A**-**B**). Suppressing METTL3 and METTL14 in LPS-stimulated RAW264.7 cells decreased collagen I production by NIH/3T3 fibroblasts when co-cultured with the supernatant from these treated cells. (**Figure S11C**). By bioinformatics analysis and methylated RNA immunoprecipitation-qPCR (MeRIP-qPCR) using fragmented mRNA, enriched m^6^A peaks on 5'UTR and CDS site of *TGF-β1* mRNA had been observed by LPS treatment (**Figure S11D**-**F**). These insights contribute to our understanding of the pathogenesis of cardiac fibrosis and remodeling. Additionally, we assessed the impact of METTL3/METTL14-mediated m^6^A modification on CXCR2 expression and its subsequent influence on chemotaxis and migration in RAW264.7 cells. LPS treatment reduced CXCR2 expression, further decreased by knockdown of METTL3 and METTL14. Conversely, overexpression of METTL3 and METTL14 would elevate CXCR2 level under LPS treatment (**Figure S12A**). Immunofluorescence and flow cytometry analyses corroborated these findings, revealing parallel trends in CXCR2 surface expression (**Figure S12B**-**D**). Transwell and wound healing assays demonstrated that LPS treatment enhanced RAW264.7 cell chemotaxis and cellular migration. Silencing METTL3 and METTL14 diminished cell chemotaxis and cellular migration, while overexpression of METTL3 and METTL14 enhanced these processes (**Figure S12E**-**H**). However, MeRIP-qPCR assays indicated that overexpression of METTL3 and METTL14 failed to increase the m^6^A modification level of CXCR2 mRNA in LPS-treated cells (**Figure S12I**-**K**), consistent with MeRIP-Seq findings on m^6^A methylation pattern in **Figure S10H**. Additionally, treatment with the NF-κB inhibitor JSH23 did not alter CXCR2 expression in cells by overexpressing METTL3 and METTL14 with or without LPS treatment, indicating that changes in CXCR2 expression were independent of the NF-κB pathway (**Figure S12L**-**M**). We also observed that METTL3 and METTL14 expression levels remained unchanged following the addition of CXCL1, a CXCR2 activator. This is consistent with the inability of CXCL1 to increase overall m^6^A methylation levels, suggesting that CXCR2 is a downstream molecule indirectly regulated by m^6^A methylation (**Figure S12N**-**O**).

In conclusion, our research revealed that m^6^A modification indirectly played a crucial role in regulating CXCR2 expression in monocytes. This modulation, primarily mediated by METTL3 and METTL14, was not directly controlled by m^6^A modification and CXCR2 activation. Instead, it suggested a complex network of regulatory mechanisms where m^6^A modification mediated CXCR2 expression through more intricate, indirect pathways. This modulation of CXCR2, coupled with the direct impact of m^6^A modification on TGF-β1 expression, highlighted the complex interplay of m^6^A modification in monocyte chemotaxis, cellular migration and pro-fibrotic response.

### The m^6^A modification governs NF-κB via *MyD88* in positive feedback mediating pro-inflammatory signaling

**Figure 5:**
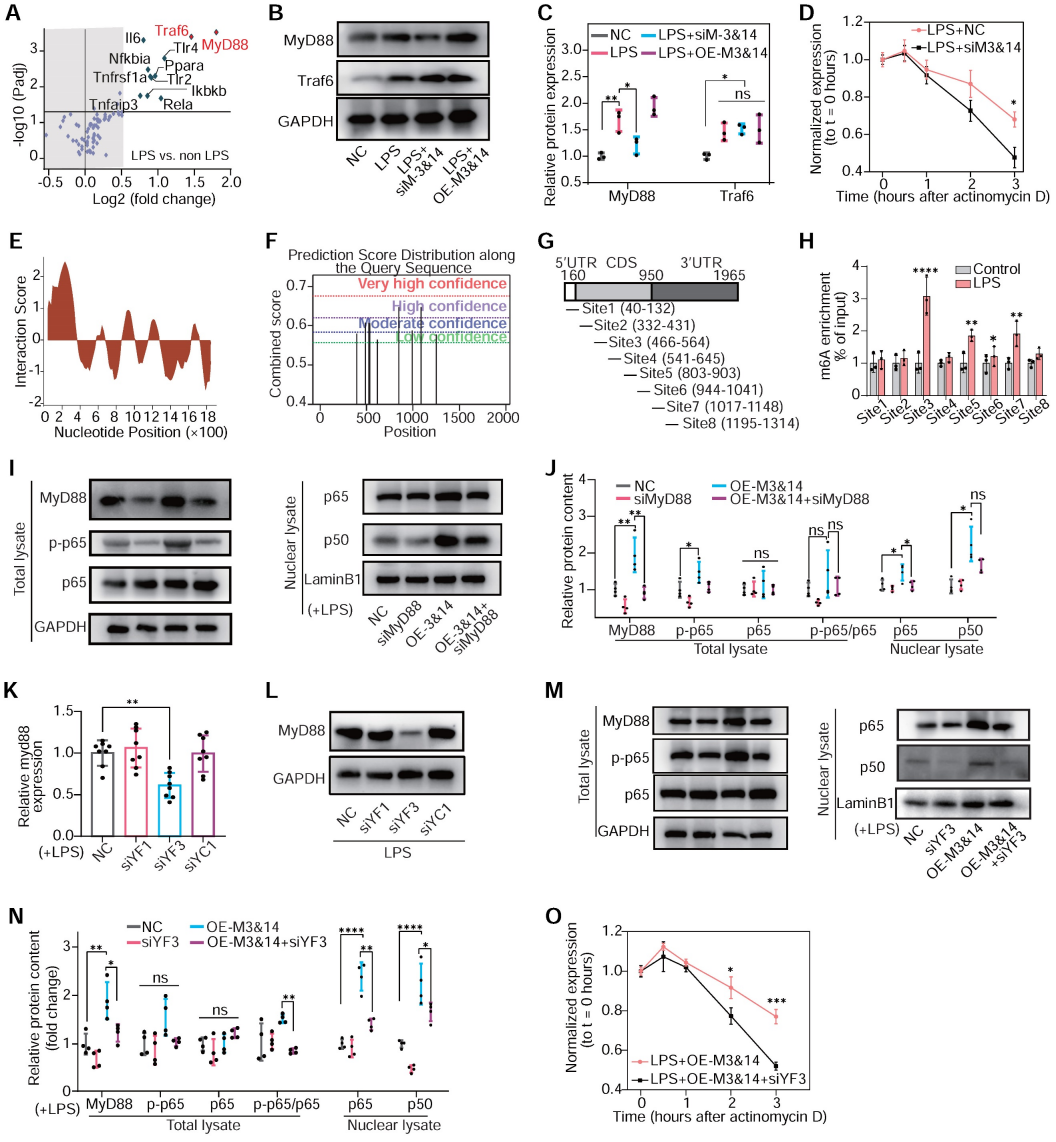
** The m^6^A modification governs NF-κB signaling in macrophage polarization.** (**A**) m^6^A abundance of mRNA associated with TLR4/NF-κB signaling pathway in LPS-treated RAW 264.7 cells detected by PCR array and MeRIP without RNA fragment procedure; n = 3 per group. (**B**) The protein levels of MyD88 and Traf6 of RAW264.7 cells after treatment with NC, LPS+NC, siM3&14, LPS+OE-M3&14. siRNAs or overexpression plasmids were transfected 24 hours before LPS treatment. (**C**) Quantification of MyD88 and Traf6 protein levels in the above cells of (B), normalized to GAPDH. n = 3 per group. (**D**) qRT-PCR analysis of *MyD88* mRNA stability in RAW264.7 cells treated with LPS+NC or LPS+siM3&14 following treatment with actinomycin D (ActD, 2.5 μg/mL). n = 5 per group. (**E**) catRAPID fragments-based prediction of interaction between METTL3 and *MyD88* mRNA and maps METTL3 interaction domains to nts 40-392 (5'-end). (**F**) Prediction sites of *MyD88* mRNA m^6^A peaks as produced from SRAMP. (**G**) Schematic of *MyD88* mRNA segments for MeRIP-qPCR primers. (**H**) MeRIP-qPCR was performed to detect the m^6^A abundance of *MyD88* in LPS-treated cells. n = 3 per group. (**I**) Immunoblot for MyD88, p65, and p-p65 (Ser536) of total lysate, p65 and p50 of nuclear lysates, which were extracted from RAW264.7 cells treated with NC, siRNA of MyD88 (siMyD88), OE-M3&14, OE-M3&14+siMyD88. (**J**) Quantification of protein contents of MyD88, p-p65, p65 and p-p65/p65 of total lysate normalized to GAPDH, p65 and p50 of nuclear lysate normalized to Lamin B1. n = 4 per group. (**K** and **L**) Expression of the MyD88 mRNA and protein were measured in RAW264.7 cells transfected with siRNA of NC, YTHDF1 (siYF1), YTHDF3 (siYF3) and YTHDC1 (siYC1), which were top 3 rated readers of predictive global scores for interaction with propensity between readers and mRNA of *MyD88*, according to catRAPID. n = 8 per group. (**M**) Immunoblot for MyD88, p65, and p-p65 (Ser536) of total lysate, p65 and p50 of nuclear lysates, which were extracted from RAW264.7 cells treated with NC, siYF3, OE-M3&14, OE-M3&14+siYF3. (**N**) Quantification of protein contents of MyD88, p-p65, p65 and p-p65/p65 of total lysate normalized to GAPDH, p65 and p50 of nuclear lysate normalized to Lamin B1. n = 4 per group. (**O**) qRT-PCR analysis of *MyD88* mRNA stability in RAW264.7 cells treated with LPS+OE-M3&14 and LPS+OE-M3&14+siYF3 following treatment with ActD (2.5 μg/mL). n = 4 per group. For (C), (D), (H), (J), (K), (N) and (O), n ≥ 3 per group. The data are expressed as the mean ± SD. P-values were determined by one-way ANOVA with Fisher's LSD post-hoc test. *, P < 0.05; **, P < 0.01; ***, P < 0.001; ****, P < 0.0001.

Next, it was critical to identify the dominant genes that were regulated by m^6^A modification and participated in macrophage inflammation and polarization. MeRIP assay and PCR arrays highlighted Myeloid Differentiation Primary Response 88 (*MyD88*) and TNF receptor-associated factor 6 (*Traf6*) as the most m^6^A-enriched genes associated with the TLR4/NF-κB pathway in LPS-treated cells (**Figure 5A**). Moreover, the inhibition of METTL3 and METTL14 downregulated *MyD88* mRNA transcription and protein expression in RAW264.7 cells, while *Traf6* remained unaffected (**Figure 5B**-**C** and **Figure S13A**-**B**). We also examined the expression of MyD88 and Traf6 in the PBMCs of patients after VSD occluder implantation, and the PCB group demonstrated a highly expressed level of MyD88 (**Figure S13C**-**D**). Employing actinomycin D to halt mRNA synthesis allowed for a precise assessment of the role m^6^A modification plays in stabilizing mRNA. The results presented that the knockdown of METTL3 and METTL14 significantly accelerated the degradation of *MyD88* mRNA, suggesting a vital role of m^6^A in its stability (**Figure 5D**). Then, the bioinformatics analysis and MeRIP-PCR with fragmented mRNA confirmed these findings, demonstrating LPS responsiveness of m^6^A peaks on *MyD88* mRNA's CDS and 3'UTR sites (**Figure 5E**-**H** and **Figure S14A**). Knockdown of MyD88 inhibited the gene set enhancement of the LPS-induced TLR4/NF-κB pathway, which was activated by *METTL3* and *METTL14* overexpression (OE-M3&14) (**Figure 5I**-**J**). These results collectively suggested that METTL3/METTL14-mediated m^6^A modification played a critical role in enhancing the stability of *MyD88's* mRNA and activating the downstream NF-κB pathway.

To further explore how m^6^A modification and its reader proteins affect *MyD88* mRNA expression and stability, three top-rated m^6^A readers of predictive global scores for interaction with propensity between readers and mRNA of *MyD88* had been estimated according to catRAPID. Knockdown of YTHDF3 significantly inhibited *MyD88* expression and TLR4/NF-κB pathway in LPS-treated RAW264.7 cells. In contrast, YTHDF1 and YTHDC1 failed to be identified to participate in *MyD88* and TLR4/NF-κB pathway regulation (**Figure 5K**-**N** and **Figure S14B**-**E**). YTHDF3 knockdown also decreased *MyD88* mRNA stability (**Figure 5O**). These results implied that METTL3 and METTL14 regulated *MyD88* expression through m^6^A modification, interacting with YTHDF3 by enhancing mRNA stability and contributing to TLR4/NF-κB pathway activation in macrophages.

Considering that NF-κB is a ubiquitous nuclear transcription factor regulating various downstream genes, we aim to investigate whether the NF-κB pathway can positively influence the expression of METTL3 and METTL14 in a feedback mechanism. We observed that the administration of NF-κB inhibitor JSH23 could attenuate LPS-induced upregulation of METTL3/METTL14 (**Figure S15A**-**B**). Bioinformatics and ChIP-PCR assays identified validated binding sites of p65 on the promoter regions of METTL3 and METTL14 (**Figure S15C**-**G**), suggesting a direct regulatory feedback loop. Thus, the m^6^A modifications mediated by *METTL3* and *METTL14* played a crucial role in modulating NF-κB pathway activation in macrophage by stabilizing *MyD88*, and the activation of NF-κB also increased the expression of *METTL3* and *METTL14* by transcriptional enhancement. This dynamic fostered an “inflammatory cascade effect”, a significant feedback mechanism in inflammation regulation.

**Figure 6:**
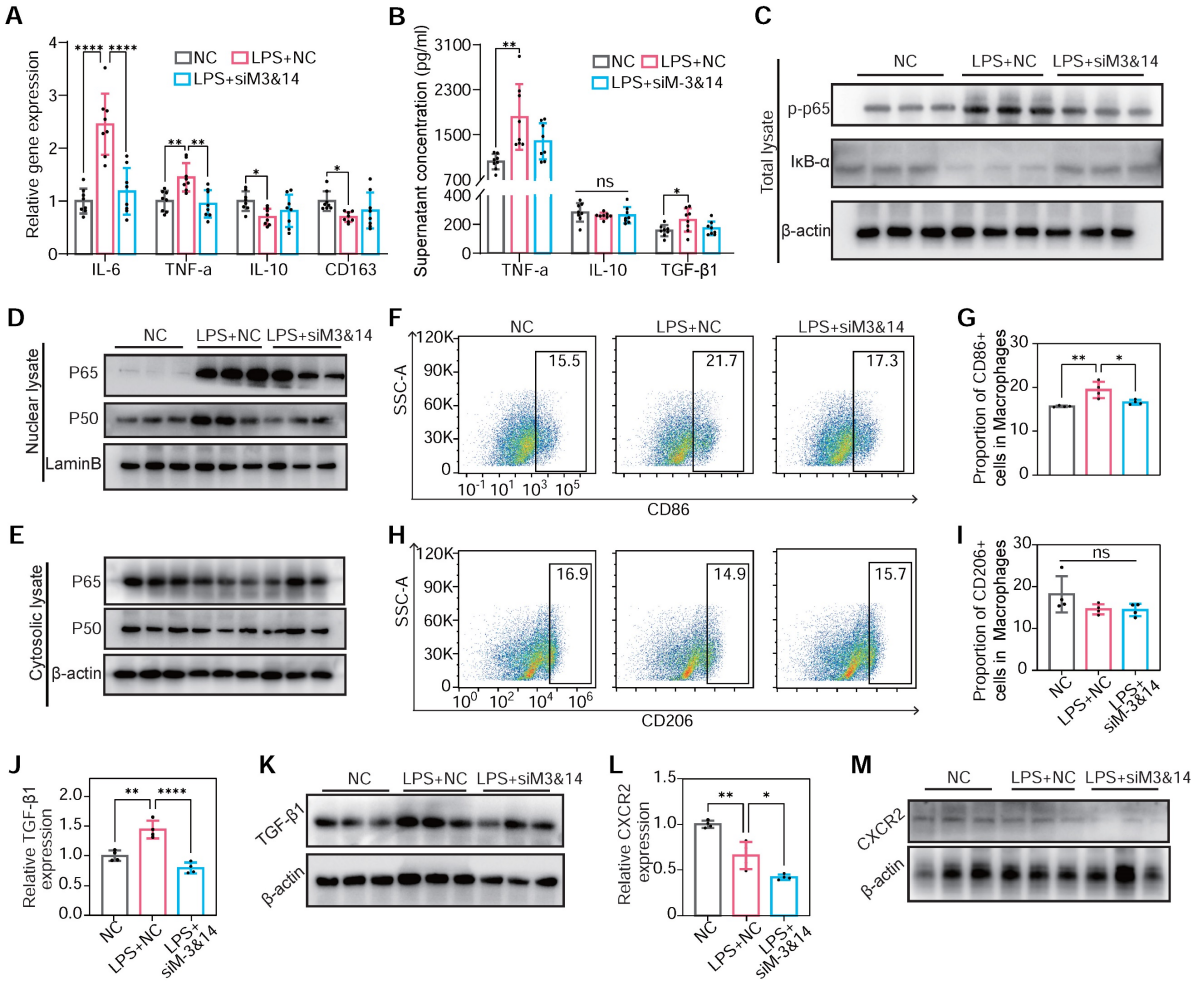
** m^6^A modification induced NF-κB activation, TGF-β1 secretion, and CXCR2 expression in METTL3/METTL14 dependent manner on BMDMs.** (**A**) mRNA expression of M1 markers of TNF-α and IL-6 and M2 markers of IL-10 and CD163 of BMDMs transfected with or without siM3&14 before LPS treatment. n = 8 per group. (**B**) TNF-α, IL-10 and TGF-β1 secretion levels in supernatants of the above cells examined by ELISA. n = 8 per group. (**C**) Immunoblot for total lysate of IκB-α and p-p65 (Ser536) in BMDMs with or without METTL3 and METTL14 knockdown before LPS treatment. (**D** and **E**) p65 and p50 immunoblotted for nuclear (D) and cytosolic lysates (E) extracted from the above cells. (**F**-**I**) Represent images and proportion results of flow cytometry for CD86 positive (M1) (F-G) or CD206 positive (M2) (H-I) cells in macrophages (CD11b^+^F4/80^+^) from BMDMs. n = 4 per group. (**J**) mRNA of *TGF-β1* were examined in BMDMs with or without METTL3 and METTL14 knockdown before LPS treatment. n = 4 per group. (**K**) Western blot of protein levels of TGF-β1 in BMDMs treated with NC, LPS+NC, LPS +siM3&14, which were cultured in medium added monensin (3µM) for 4 h before harvesting of cells. (**L**-**M**) mRNA level and protein content of CXCR2 in cells in (C-I). n = 4 per group. For (A), (G), (H), (I), (J), (L), (N) and (O), the data are expressed as the mean ± SD. P-values were determined by one-way ANOVA with Fisher's LSD post-hoc test. *, P < 0.05; **, P < 0.01; ***, P < 0.001; ****, P < 0.0001.

To validate the results on METTL3/METTL14 mediating macrophage polarization in RAW264.7 cells, BMDMs were used to draw a convincing conclusion. The inhibition of METTL3 and METTL14 reduced M1 gene markers and promoted the expression of M2 markers in LPS-treated BMDMs. This alteration also reduced the expression of TNF-α, TGF-β1, and IL-10 (**Figure 6A**-**B**). Examining the IκB-α, p65, and phosphor-p65 (Ser536) levels before and after LPS treatment, the inhibition of NF-κB pathway and prevention of p65 and p50 nuclear translocation following METTL3 and METTL14 knockdown had been observed (**Figure 6C**-**E**). A reduction in CD86-positive (M1) macrophage percentage upon METTL3 and METTL14 knockdown had been assessed by flow cytometry, indicating a shape in macrophage polarization (**Figure 6F**-**I**). Concurrently, TGF-β1 and CXCR2 protein content decreased in LPS-treated BMDMs post-knockdown of METTL3 and METTL14 (**Figure 6J**-**M**). These findings in BMDMs align with observations in RAW264.7 cells, underscoring a consistent role for METTL3/METTL14-mediated m^6^A modification in regulating the NF-κB pathway, macrophage polarization, and pro-fibrotic cytokine secretion.

### Characterization and drug-loading of bioengineered erythrocyte microvesicles

Underlining the molecular mechanisms of m^6^A in participating in myocardial fibrosis and remodeling, it pointed toward novel intervention strategies, including targeting METTL3 and METTL14 in peripheral blood monocytes. However, our current understanding was limited, as BMDMs modified *in vitro* might not accurately reflect the activation state of monocytes in peripheral blood* in vivo*. One of the challenges was the targeted intervention of peripheral blood monocytes, compounded by the absence of specific promoters for monocyte-specific gene knockout 45. We investigated bioengineering strategies targeting peripheral blood monocytes with high Ly6C expression to tackle this challenge. We applied STM2457 to inhibit the METTL3-mediated m^6^A modifications in these cells, evaluating the impact on myocardial fibrosis and cardiac remodeling. This method is part of our broader goal to develop an efficient, cost-effective treatment strategy that improves outcomes in clinical therapy for heart conditions.

To specifically target peripheral blood monocytes with a small molecule inhibitor of METTL3 (STM2457), we leveraged monocytes' inherent ability to phagocytose erythrocyte MVs. Based on this biological process, we developed a biohybrid microvesicular system, utilizing erythrocyte MVs as the carrier. Our study explored various methods to induce MVs from donor erythrocytes, including treatments with PBS, A23187, and t-BOOH for 24 hours and prolonged storage at 4°C for 10 days. Notably, t-BOOH treatment significantly enhanced annexin V expression in MVs, suggesting increased macrophage recognition and phagocytosis potential (**Figure S16**). Purified MVs from erythrocytes were enriched with typical MV markers, including ALIX and TSG101, and Stomatin (STOM), a specific marker of erythrocyte-derived MVs. (**Figure S17**). Flow cytometry analysis demonstrated that erythrocyte-derived MVs, identified by the erythrocyte-specific antigen CD235a, exhibited minimal contamination from platelet-derived MVs, which were the most abundant MVs in the bloodstream and marked by CD41 (**Figure S18**).

Western blot analysis of BMDMs showed Hemoglobin A (HBA) uptake after exposure to erythrocyte-derived MVs, an effect absent in control cells not treated with MVs (**Figure S19**). Trypsin treatment, which targets surface-bound MVs, had little effect on the DiR signal, indicating that MV uptake transcends passive adsorption. Inhibition experiments involving cooling, energy depletion agents (NaF, NaN3, antimycin A), and endocytosis inhibitors (cytochalasin D, dynasore) significantly reduced MV internalization, confirming its reliance on active cellular processes rather than membrane fusion or nonspecific binding. Moreover, introducing heparin reduced MV uptake by 60-70%, highlighting the role of heparan sulfate proteoglycans in mediating this process (**Figure S20**). Continuing with nanoparticle (NP) assembly, we employed a combination of epigallocatechin gallate (EGCG), trivalent iron ions (Fe^3+^), STM2457, and coumarin 6, maintaining a mass ratio of STM2457:coumarin 6 for 5:1. Coumarin 6, a hydrophobic fluorescent small molecule, was used for fluorescent labeling of the NPs 46. We constructed an MV-based hybrid system utilizing a three-step MPN method. Unlike biomacromolecules, the binding capacity of hydrophobic small molecules with MPN is relatively low. To enhance the loading efficiency and capacity of the MVs, in the first step, we created tiny pores on the MVs using low-power ultrasound while adding EGCG, STM2457, and coumarin 6. After a 10-minute shaking incubation, Fe^3+^ was introduced with ultrasonic treatment to initiate the assembly process. To enhance the solubility and biocompatibility of the drug-loaded MVs, polyethylene glycol (PEG) was incorporated. 47,48 Finally, the drug-loaded MVs were standardized using an Avanti extruder, passing them through a filter membrane with a pore size of 250 nm to ensure uniformity. The MVs forming MPN on their surface and interior for drug loading are referred to as STM@MV*nex-u*. The drug-loaded MV system created without ultrasound assembly and Avanti extruder is termed STM@MV*nex,* and the nanoparticles formed without the assembly of mixed MVs or STM2457 are referred to as STM@NP*nex-u* or MV*nex-u*, respectively.

**Figure 7:**
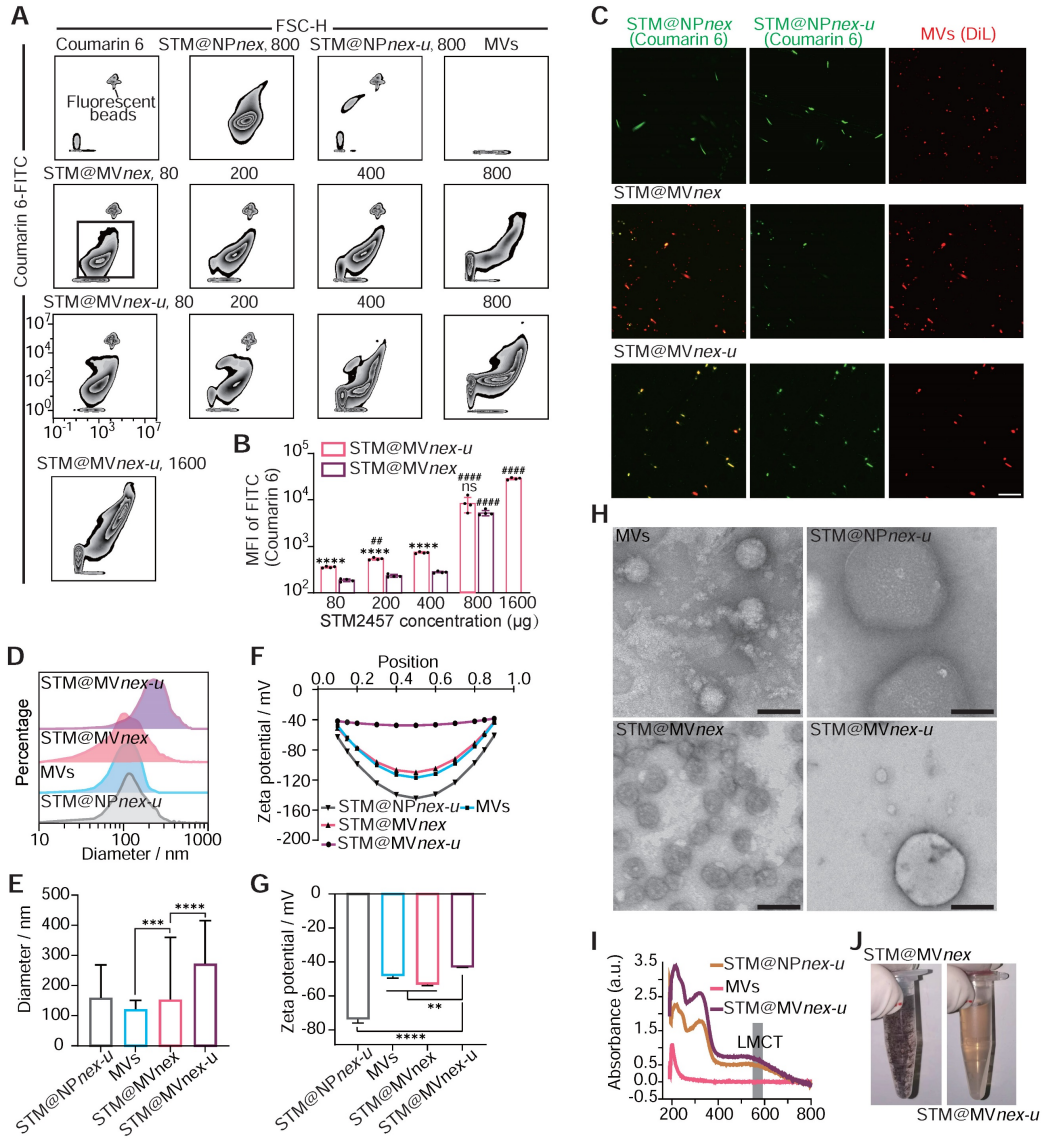
** Assembly and characterization of drug**-**loaded nanoparticles on erythrocyte microvesicles.** (**A**) Flow cytometric analysis illustrating the increase in fluorescence intensity of drug-loaded nanoparticles corresponding to rising concentrations of the STM2457. The fluorescent beads are labeled with FITC fluorescence and have a diameter of 400 nm. (**B**) Quantitative assessment of fluorescence intensity on STM@MV*nex* and STM@MV*nex-u* across varying drug concentrations. n = 4 per group. * denotes significance between groups STM@MV*nex* and STM@MV*nex-u* at the identical STM2457 concentration. ^#^ denotes significance within STM@MV*nex* and STM@MV*nex-u* compared to the nearest preceding concentration. (**C**) LSCM images showcasing STM@NP*nex*, STM@NP*nex-u*, MVs, STM@MV*nex*, and STM@MV*nex-u*. Microvesicles are marked with DiL (red), and drug-loaded NPs are labeled with coumarin 6 (green). Scale bars, 10 μm. (**D** and **E**) DLS measurements depicting the size distribution and diameter quantification of STM@NP*nex-u*, MVs, STM@MV*nex*, and STM@MV*nex-u*. (**F** and **G**) Zeta potential analysis and its quantification for STM@NP*nex-u*, MVs, STM@MV*nex*, and STM@MV*nex-u*. (**H**) TEM images providing detailed insights into particle morphology. Scale bars, 200 nm. (**I**) UV-visible absorption spectra for STM@NP*nex-u*, MVs, and STM@MV*nex-u*, highlighting the emergence of the LMCT band. (**J**) Photographs of STM@MV*nex* and STM@MV*nex-u* after 30 minutes of settling, demonstrating their stability. For (A), (E), and (G), statistical analyses were performed using one-way ANOVA with Fisher's LSD post-hoc test. All data are expressed as mean ± SD *, P < 0.05; **, P < 0.01; ***, P < 0.001; ****, P < 0.0001.

Subsequently, we quantified the proportion of NPs formed from MVs using the erythrocyte marker Ter-119a. Flow cytometry analyses demonstrated a decrease in the proportion of Ter-119a positive nanoparticles as the concentrations of EGCG increased. This observation suggested the formation of MPN nanoparticles not associated with MVs. Based on these findings, a 0.4 mg/mL concentration for EGCG was chosen for the following experiments (**Figure S21A**-**B**). Moreover, flow cytometry analysis revealed a direct correlation between increased STM2457/coumarin 6 concentration and the fluorescence intensity of drug-loaded nanoparticles, leading to the selection of an optimal concentration of 0.8 mg/mL STM2457 for nanoparticle assembly based on the observed plateau in the fluorescence intensity of STM@MV*nex-u* beyond this concentration (**Figure 7A**-**B**). High-performance liquid chromatography (HPLC) confirmed the efficiency of STM2457 loading into/onto MVs after sequential extrusion was 8.7% for STM@MV*nex-u* and 5.6% for STM@MV*nex*, respectively. With an increase from 0.8 mg/mL in the concentration of STM2457 within a specific range, the loading capacity of STM@MV*nex-u* does not exceed 9.5%. Further, LSCM confirmed the successful integration of hydrophobic molecules onto/into the MVs, evidenced by dual fluorescence in MVs (red fluorescence from DiL) and the loaded drug (green fluorescence from coumarin 6) (**Figure 7C**). Interestingly, compared to STM@MV*nex-u*, many NPs in STM@MV*nex* group displayed only coumarin 6 fluorescence, suggesting independent MPN formation from MVs and increased drug loading capacity using ultrasound of the three-step MPN formation method. Dynamic light scattering (DLS) measurements further supported these findings, showing STM@NP*nex-u* and MVs diameters ranging between 100-200 nm. In comparison, STM@MV*nex* and STM@MV*nex-u* exceeded 250nm, indicating successful assembly (**Figure 7D**-**E**). Zeta potential measurements also validated effective drug loading, with STM@NP*nex-u* zeta potential below -70mv, MVs, and STM@MV*nex* below -45mv, and STM@MV*nex-u* around -38mv (**Figure 7F**-**G**).

Transmission electron microscopy (TEM) offered more profound insights into the morphology of the nanoparticles and their interaction with the MPN (**Figure 7H**). Significantly, STM@MV*nex* showcased independent nanoparticles connected to MVs instead of the cargo distributed around the membrane of the MVs in STM@MV*nex-u*. Therefore, the actual loading efficiency of STM@MV*nex* is significantly lower than the measured values. UV-visible absorption spectra analysis revealed the emergence of a characteristic ligand-to-metal charge transfer (LMCT) band at approximately 570 nm, a clear indicator of metal-phenolic coordination (**Figure 7I**). After a 30-minute settling period, STM@MV*nex-u* demonstrated enhanced stability compared to STM@MVnex, likely due to the more adsorption of MPN onto MVs, reducing the concentration of EGCG and Fe^3+^ in the solution. (**Figure 7J**). Finally, the BCA method, utilizing serial twofold dilutions of STM@MV*nex-u*, demonstrated a linear correlation between coumarin 6 fluorescence units, MV quantity, and MV protein content, confirming the consistency and reliability of our drug-loading and nanoparticle assembly processes (**Figure S21C**).

### Enhanced targeting of MV*nex-u* for inflammatory monocytes/macrophages in blood and heart

**Figure 8:**
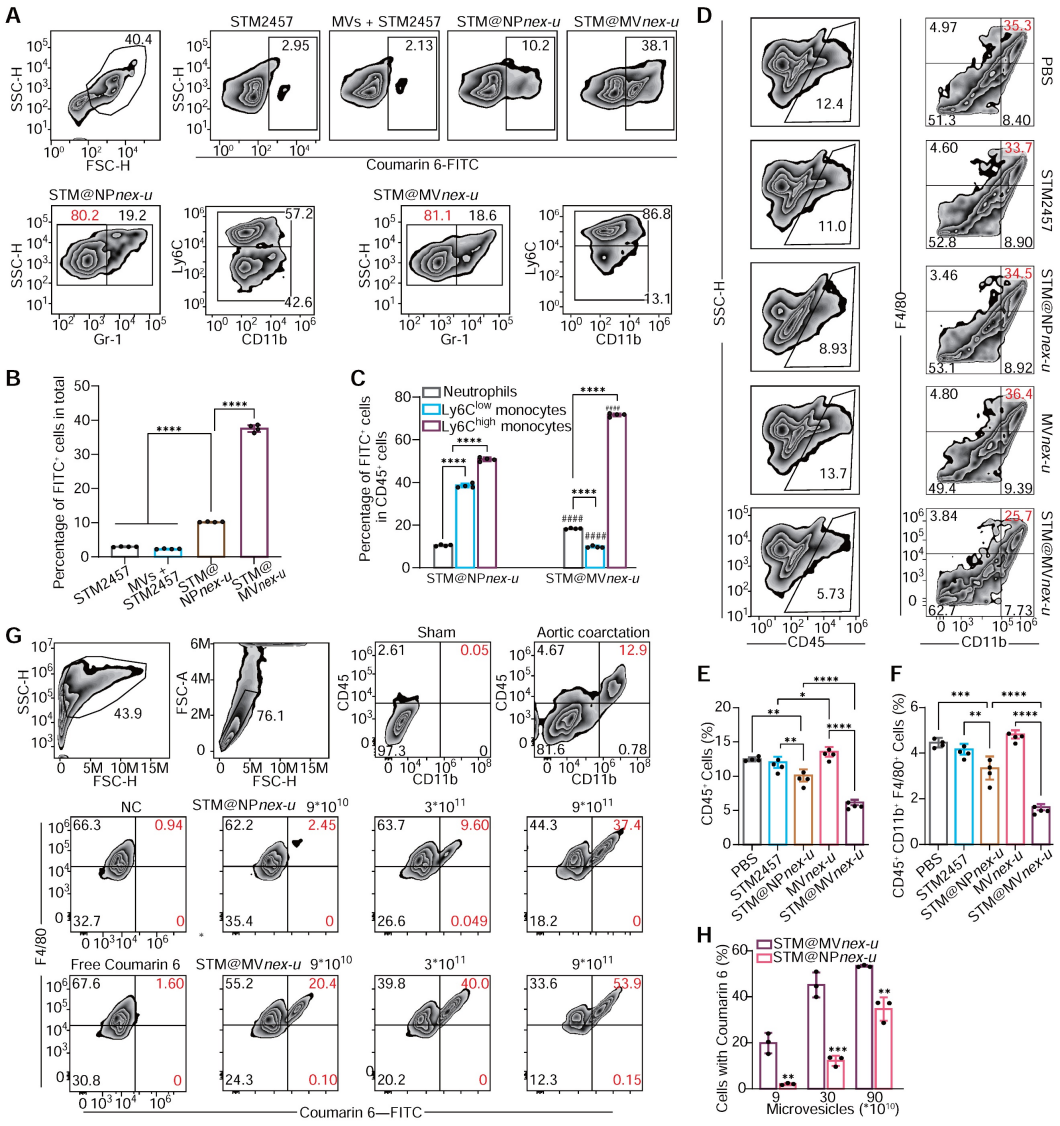
** Evaluation of nanoparticle uptake and targeting in blood and heart.** (**A**) Flow cytometry analysis of blood cells post-treatment with STM2457, MVs+STM2457, STM@NP*nex-u*, and STM@MV*nex-u*, showing the proportion of coumarin 6 FITC-positive cells. The proportions of the target cell population were marked with red numerical labels. (**B**) Quantitative comparison of FITC-positive cells in blood following each treatment. n = 4 per group. (**C**) Quantification of different cell types within coumarin 6 FITC-positive cells. n = 4 per group. (**D**) Flow cytometry of cardiac cells post-treatment for macrophage analysis. (**E**) Quantification of CD45^+^ white cells in cardiac tissue. n = 4 per group. (**F**) Quantification of macrophage proportions in cardiac tissue. n = 4 per group. (**G**) Flow cytometry of coumarin 6 FITC-positive cells in cardiac tissue post-treatment. (**H**) Quantification of coumarin 6-positive cell proportions in the heart. n = 3 per group. For (B), (C), (E), (F), and (H), statistical analyses were performed using one-way ANOVA with Fisher's LSD post-hoc test. All data are expressed as mean ± SD. *, P < 0.05; **, P < 0.01; ***, P < 0.001; ****, P < 0.0001.

Plasma MVs were analyzed using DLS, which showed that two hours after the injection of 2×10^11^ MVs, the proportion of endogenous MVs was nearly negligible (**Figure S22**). Flow cytometry analysis assessed the cellular uptake and targeting efficiency of free STM2457 (containing 20% by mass of coumarin 6), MVs+STM2457, STM@NP*nex-u*, and STM@MV*nex-u* in blood cells (**Figure 8A**). This examination revealed a substantial increase in coumarin 6 FITC-positive cells, particularly following STM@MV*nex-u* treatment, which exhibited the highest proportion of such cells (**Figure 8B**). This finding suggests the superior uptake efficiency of STM@MV*nex-u*, distinct from the effects of MPN alone. Furthermore, a significant shift in cell type proportions within the FITC-positive population was noted. After the STM@NP*nex-u* treatment, most of these cells were Ly6C-high- and Ly6C-low-expressing monocytes. However, after STM@MV*nex-u* administration, there was a notable increase in Ly6C high-expressing cells, implying targeted drug delivery to a specific monocyte subset (**Figure 8C**). Following tail vein injection of MVs or STM@MV*nex-u* (pre-labeled with DIR fluorescence), STM@MV*nex-u* showed a slighter decrease in MVs levels, indicating that the MPN on MVs may provide a protective effect against rapid* in-vivo* clearance mechanisms (**Figure S23A**-**B**) 49,50. These findings laid the groundwork for potential therapeutic interventions targeting inflammatory monocyte-mediated adverse events.

Analysis of cardiac single-cell suspensions revealed a notable reduction in macrophage populations following STM@MV*nex-u* treatment (**Figure 8D**), coupled with a decrease in CD45^+^ white blood cell infiltration, indicating its anti-inflammatory potential (**Figure 8E**-**F**). Notably, a substantial proportion of FITC-positive cells in the heart, predominantly macrophages, was observed in the STM@MV*nex-u* group (**Figure 8G**-**H**), consistent with blood cell uptake patterns and indicating effective blood monocyte and cardiac macrophage targeting. Following the administration of high doses of MVs or STM@MV*nex-u*, most non-myocardial cells displayed DIR positivity, suggesting a wider distribution of MVs compared to the pattern seen with coumarin 6-labeled nanoparticles (**Figure S23C**-**D**). This likely results from monocytes/macrophages ingesting MVs and releasing new DIR-labeled exosomes without nanoparticles, affecting neighboring cells and resulting in a higher prevalence of DIR fluorescence 51.

### Therapeutic efficacy and safety assessment of STM2457@MV*nex-u* for cardiac fibrosis *in vivo*

**Figure 9:**
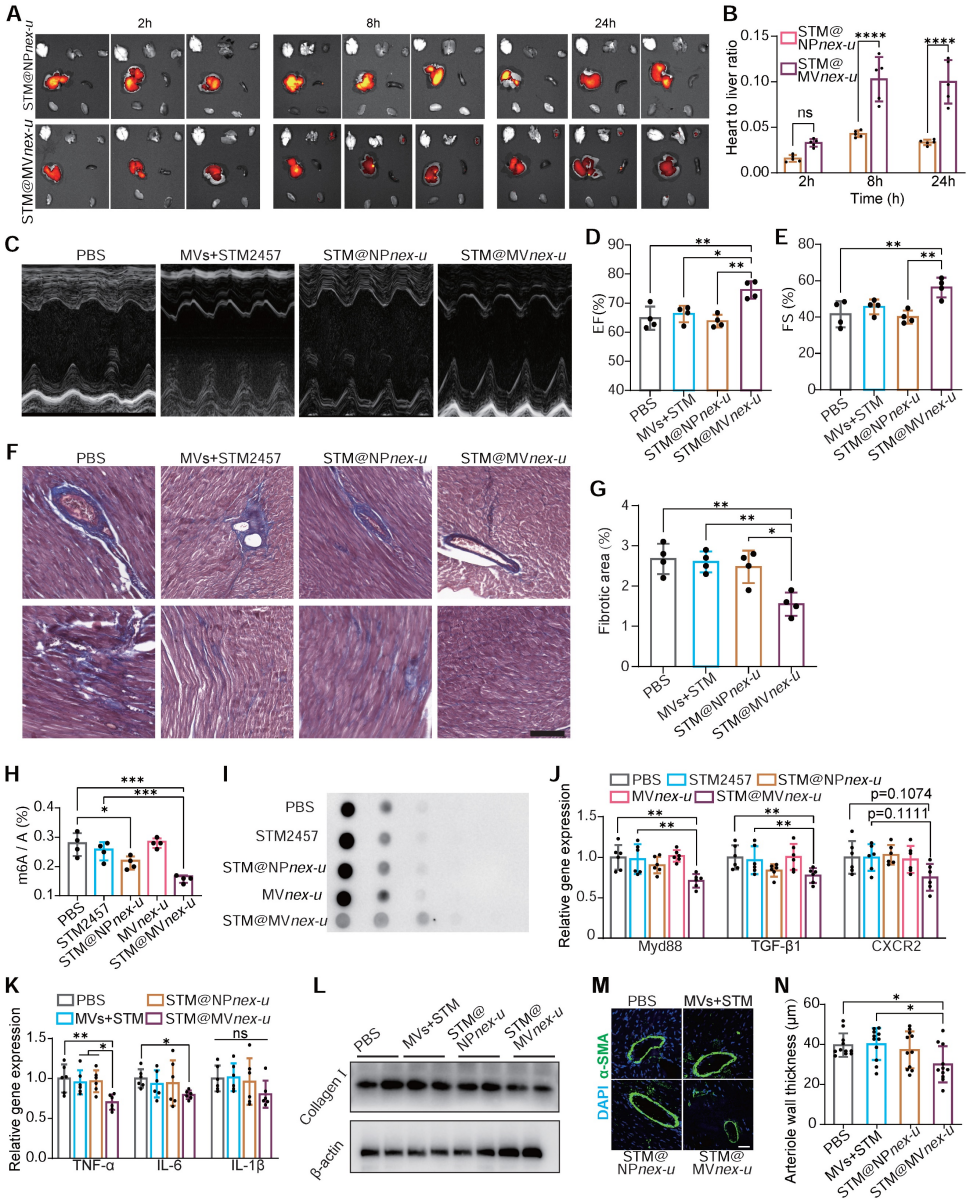
** Comprehensive evaluation of m^6^A RNA modification in cardiac macrophages and its correlation with post**-**treatment myocardial structural and functional improvement.** (**A**) IVIS imaging of DIR-loaded STM@MV*nex-u* or STM@NP*nex-u* accumulation in the heart post-treatment. (**B**) Heart targeting index (heart-to-liver fluorescence ratio) analysis of DIR. n = 5 per group. (**C**-**E**) M-mode echocardiography of left ventricular chamber (C), and measurement of ejection fraction (EF%) (D) and fractional shortening (FS%) (E). n = 4 per group. (**F** and **G**) Massons' trichrome staining of heart tissues (F), and quantification of fibrotic area (G). n = 4 per group. (**H** and **I**) Assessment of m^6^A RNA modification levels by colorimetric (H) and dot blot (I) analyses in cardiac macrophages from Ang II-induced mice treated with PBS, STM2457, STM@NP*nex-u*, MV*nex-u* and STM@MV*nex-u*. n = 4 per group. (**J**) qPCR analysis of MyD88, TGF-β1, and CXCR2 mRNA levels in cardiac macrophages post-treatment. n = 6 per group. (**K**) qPCR analysis of M1 macrophage markers including TNF-α, IL-6, and IL-1β of mice treated with PBS, STM2457, STM@NP*nex-u* or STM@MV*nex-u*. n = 6 per group. (**L**) Western blot analysis of Collagen I expression in the left ventricle post-treatment. (**M**) Immunofluorescence of arteriolar smooth muscle (SM) hyperplasia (SMA-stained, green fluorescence) in the left ventricle at 28 days post-treatment. Scale bar, 20 μm. (**N**) Quantitative measurement of large arteriolar wall thickness (M). n = 11 per group from at least 3 mice. Large arterioles (α-SMA-positive vessels with diameters over 20 μm) were analyzed to assess therapeutic effects in the left ventricle. For (B), (D), (E), (G), (H), (J), (K), and (N), statistical analyses were performed using one-way ANOVA with Fisher's LSD post-hoc test. All data are expressed as mean ± SD *, P < 0.05; **, P < 0.01; ***, P < 0.001; ****, P < 0.0001.

*In vivo* imaging system (IVIS) demonstrated significant cardiac enrichment of DIR-loaded nanoparticles following treatment (**Figure 9A**). The heart targeting index, defined as the ratio of heart-to-liver fluorescence intensity, was substantially higher for STM@MV*nex-u* compared to STM@NP*nex-u*. Specifically, the index for STM@MV*nex-u* reached over 0.12, while the highest value for STM@NP*nex-u* did not exceed 0.05 (**Figure 9B**). Due to the effective targeted administration of drugs to cardiac macrophages, there was a substantial improvement in the heart function of Ang II-treated mice (**Figure 9C**-**E**). This strategic delivery also led to a considerable decrease in fibrosis in the heart (**Figure 9F**-**G**).

The molecular efficacy of STM@MV*nex-u* was further substantiated at a cellular level. Post-treatment analysis of cardiac macrophages in Ang II-induced mice revealed decreased m^6^A modification levels of total mRNA (**Figure 9H**-**I**). RT-qPCR analysis demonstrated a significant reduction in the expression of key regulatory factors of *MyD88*, *TGF-β1*, and *CXCR2* in cardiac macrophages. (**Figure 9J**). These results were complemented by a substantial reduction in pro-inflammatory M1 macrophage markers, including TNF-α, IL-6, and IL-1β (**Figure 9K**), showcasing STM@MV*nex-u*'s anti-inflammatory properties. Western blotting confirmed decreased collagen I expression in the left ventricle following STM@MV*nex-u* treatment (**Figure 9L**). Additionally, analysis of α-SMA in the left ventricle post-MI revealed a reduction in smooth muscle hyperplasia in large arterioles (diameter >20μm) in STM@MV*nex-u* treated mice (**Figure 9M**-**N**), suggesting its role in alleviating inflammation-induced vascular alterations.

Toxicity evaluations at one and three months post-administration of STM@MV*nex-u* via tail vein injections in mice demonstrated no significant adverse effects. At the one-month mark, a comprehensive analysis of blood and urine parameters, including levels of aminotransferases, alkaline phosphatase, creatinine, bilirubin, and total proteins, as well as complete blood cell counts, showed no deviations from baseline (**Figure S24**). Histological examinations confirmed the absence of discernible damage to major organs (**Figure S25**). Subsequent evaluations at three months maintained that clinical chemistry and hematological parameters remained stable (**Figure S26**). Additionally, assessments of iron and ferritin concentrations in serum and liver, conducted using colorimetric and ELISA techniques, indicated consistent levels, verifying the long-term safety of the Fe^3+^ component in STM@MV*nex-u* (**Figure S27**).

These results demonstrate that STM@MV*nex-u* effectively modulates inflammatory responses and macrophage activity by inhibiting m^6^A modification through STM2457. This finding aligns with our prior research, which identified a strong link between m^6^A modification in monocytes and their migration, inflammation, and fibrosis within cardiac pathology. Targeting m^6^A modification thus emerges as a promising strategy for treating cardiac fibrosis and remodeling. Additionally, using t-BOOH-induced MVs combined with MPN and STM2457 represents a practical therapeutic approach in these pathological conditions.

## Discussion

This research elucidates the pivotal role of m^6^A RNA methylation in cardiac remodeling and fibrosis, highlighting the modulation of monocyte and macrophage activity through METTL3 and METTL14 enzymes. Our findings demonstrate a significant upregulation of these methyltransferases in monocytes from patients with VSD occluder implantation-associated CB. Monocytes' contribution to cardiac inflammation and fibrosis is further exemplified by the altered expression of CXCR2 and TGF-β1, critical factors in monocyte migration and fibrosis.

Central to our findings is the impact of m^6^A modification on *MyD88* mRNAs. The study unveils a novel feedback mechanism in which NF-κB transcription factors upregulate METTL3 and METTL14, leading to enhanced m^6^A modification on *MyD88* mRNA. This modification notably increases the stability of *MyD88* mRNA by recognition of YTHDF3, subsequently elevating MyD88 expression. This feedback loop is critical in influencing macrophage polarization and activating the TLR4/NF-κB signaling pathway. On the other hand, the overexpression of METTL3 and METTL14 leads to increased infiltration of monocytes into damaged myocardial tissue and elevates the production of pro-fibrotic factors. This effect is mediated through the upregulation of CXCR2, enhancing monocyte migration, and through the elevated expression of TGF-β1, boosting their pro-fibrotic capacity. This discovery underlines the intricate connection between m^6^A methylation and the vital function and fate of monocytes/macrophages, which is essential for comprehending cardiac pathology.

Our study employs bio-nanotechnology to establish a link between m^6^A modification in peripheral blood monocytes and Ang II-induced cardiac remodeling and fibrosis. Innovatively, we have developed a monocyte-hitchhiked erythrocyte MVs-based system for delivering a METTL3 inhibitor, STM2457, targeted at inflammatory monocytes, which ultimately become the macrophages in the heart. This method revolutionizes how monocytes infiltrate, differentiate, and function within cardiac tissues, leading to optimized drug delivery and reduced inflammation and fibrosis, ultimately enhancing cardiac health. The observed improvements strongly support our hypothesis regarding the critical role of m^6^A modification in monocyte functionality for cardiac tissue. In contrast to conventional gene editing, our bio-nanotechnological approach is more efficient, bypassing the laborious process of generating transgenic mice, thereby cutting down on time and cost. Additionally, it provides a solution to the limitations posed by the lack of specific promoters for CRISPR gene editing in monocytes through targeted delivery capabilities. This strategy offers therapeutic benefits and paves the way for new methods in biological research for particular cell types, expanding the scope of nanotechnology's applications 52.

Despite the promising results, transitioning these findings to clinical settings involves notable challenges. We observed m^6^A modification changes and varied gene expressions in the PBMCs of patients with VSD occluder implantation-associated CB. It's critical to note that PBMCs contain monocytes and many other leukocytes. While these arrhythmias are intricately linked with local edema, inflammation, and fibrosis, other factors, such as the stimulated Purkinje system's potential role in inducing arrhythmias or the impact of metallic occluders on cardiac electrical conduction, cannot be overlooked 53. Furthermore, our inability to create VSD closure models in animals led us to adopt an Ang II-induced mice model for cardiac fibrosis and remodeling. This model was preferred due to its relatively minor trauma and fewer confounding factors in fibrosis research, especially when compared to myocardial infarction models, where extensive damage and ischemia-hypoxia-influenced outcomes could overshadow the role of monocytes in cardiac prognosis. Despite these considerations, the dissimilarities between mouse models and human patients are significant and cannot be ignored. Lastly, although we utilized multiple control groups to exclude the impacts of MVs and the MPN system, the potential influence of components such as MVs, iron ions, and EGCG, and how these might influence the destiny of monocytes are aspects that have not been completely clarified in our study 28,54-57.

This study marks a significant advance in cardiovascular research, revealing the transformative role of m^6^A methylation in monocyte and macrophage function for cardiac pathology and opening new pathways for targeted treatments in cardiac remodeling and fibrosis. It represents a pivotal step in understanding and addressing complex cardiac diseases through innovative molecular mechanisms and bioengineered approaches.

## Materials and Methods

*Study population and ethics statement:* This study was approved by the Ethics Committee of West China Second Hospital of Sichuan University (**2019**-**069**). For the participants, we obtained written informed consent to participate in this research from all the reported 97 patients' parents, including the patients' blood in subsequent studies and publications. All patients received transcatheter closure of VSD at our center and were diagnosed by transthoracic echocardiography and/or clinical evidence of left-to-right interventricular shunt. Before the closure procedure, patients were evaluated by transthoracic echocardiography. Inclusion and exclusion criteria were previously described in detail 58.

*General materials*: Epigallocatechin gallate (EGCG, 95%) was purchased from Sigma Aldrich (USA). Ferric (III) chloride hexahydrate (FeCl3·6H2O, ≥99.0%) was purchased from Chron Chemical Co., LTD (Shanghai, China). STM2457 (99.06%) was purchased from Targetmol (Shanghai, China). PBS buffer was purchased from Adamas life (China). All reactants were of analytical purity grade and were used as received without further purification. 6-well plates, 24-well plates, and 96-well plates were supplied by Jetbiofil Cnreagent Co., Ltd (Guangzhou, China). Dulbecco's Modified Eagle Medium (DMEM), Roswell Park Memorial Institute 1640 (RPMI 1640), Fetal Bovine Serum (FBS), penicillin-streptomycin, Trypsin-EDTA solution (0.25%), giemsa stain, 4,6-diamidino-2-phenylindole (DAPI) and 1,1-dioctadecyl-3,3,3,3-tetramethylindotricarbocyanine iodide (DiR) were purchased from Sigma-Aldrich (USA). Cell Counting Kit-8 was sourced from KGI Biotechnology Co., Ltd (Jiangsu, China). High-purity Milli-Q (MQ) water with a resistivity of 18.2 MΩ cm was obtained from an inline Millipore RiOs/Origin water purification system. All solutions were freshly prepared for immediate use in each experiment.

*Isolation of peripheral blood monocytes:* Collect fresh anticoagulated whole blood and dilute it with an equal volume of DPBS. Mix reagents A and D (A:D volume ratio is 3:2) of PBMC isolation regent kit (Solabri P8680 for human, P5230 for mouse) in a sterile centrifuge tube to form a clear gradient interface and ensure clear stratification. Spread diluted blood on the top of the separation fluid and maintain a clear interface. Centrifuge at room temperature at 500-800g for 20-30min and adjust conditions for optimal separation (max speed 1000g). Transfer mononuclear cells to a sterile centrifuge tube and add 10 mL PBS to mix. Centrifuge at 250g for 10 minutes, discard the supernatant and resuspend cells in PBS. Purify cells by differential adhesion method using mononuclear cell culture medium at a 10^6^ cells/mL density, and incubate for 2-4 hours to obtain monocytes adhered to the surface.

*Cytokine Quantification:* CXCL1, CXCL2, CXCL3, CXCL7, TNF-α, IL-10, TGF-β1 and IL-6 concentrations in the plasma and in cell culture supernatant were quantified using enzyme-linked immunosorbent assay kits purchased from Multisciences (Hangzhou, China) according to the manufacturer's instructions.

*Quantitative real-time PCR analysis:* NucleoZOL Kit (740404.200) isolated small and large RNA in two separate fractions. Genomic DNA removal and reverse transcription for large RNA were performed using PrimeScript™ RT reagent Kit with gDNA Eraser (Takara, RR047A). Real-time PCR for large RNA was performed using a Biorad CFX96 with TB Green Advantage qPCR Premix User Manual (Takara, 639676), and qPCR primers were listed in **Table S4**.

*Western blot:* cells or tissues were lysed in the RIPA lysis buffer system (Santa Cruz Biotechnology, sc-24948) with Mini Protease Inhibitor Cocktail Tablets (cOmplete, 4693124001). Cytosolic lysate and nuclear lysate were separated by CelLytic/NuCLEA Extraction Kit (Solarbio, R0050). Total protein concentrations were normalized using BCA analysis (Life Technologies, 23227). After boiling with 4× loading buffer for 5 min, 20 μL cell lysate of each sample was separated on an 8-12% gel, transferred to a PVDF membrane, and blocked by 4% BSA/TBST. Primary antibodies of METTL3 (1:2000, Abcam, ab195352), METTL14 (1:2000, Abcam, ab309096), CXCR2 (1:2000, Abcam, ab89254), TGFβ1 (1:2000, Abcam, ab215715), iNOS (1:2000, Abcam, ab178945), iNOS (1:2000, Abcam, ab178945), Arg1 (1:2000, Proteintech, 16001-1-AP), IκB-α (1:2000, Proteintech, 10268-1-AP), c, phosphor-p65 (1:2000, Abcam, ab76302) phosphor-IκB-α (1:2000, Abcam, ab92700), F4/80 (1:2000, Abcam, Ab300421), MyD88 (1:2000, proteintech, 67969), Collagen1 (1:2000, proteintech, 14695), β-actin (1:2000, proteintech, 81115), p50 (1:2000, CST, 13586), Lamin B1 (1:2000, proteintech, 12987-1-AP), Traf6 (1:2000, Abcam, ab33915), α-SMA (1:2000, CST, 14968), CD86 (1:2000, Abcam, 19589), GAPDH (1:2000, ProteinTech, 60004), were incubated with the membrane overnight at 4 °C. After washing, membranes were incubated with HRP-conjugated secondary antibodies for at least 45 min. Immunoblots were then treated with Immobilon Western chemiluminescent HRP substrate (Millipore, WBKLS0500). Chemiluminescence was detected using a BioRad blot scanner.

*Flow cytometry*: all flow cytometry was performed on a BD FACSCelesta™ Flow Cytometer (BD Biosciences, San Jose, CA) and analyzed with FlowJo (Tree Star, Ashland, OR). All Antibodies for flow cytometry were purchased from BioLegend and BD biosciences: APC anti-CD11b (101211; Biolegend), BV421 anti-CD206 (C068C2, Biolegend), BV510 anti-Ly6C (HK1.4, Biolegend), BV605 anti-F4/80 (BM8, Biolegend) and FITC anti-CD86 (105005, Biolegend). Cells were washed using cell staining buffer (Biolegend, cat # 420401) and subjected to staining with surface antibodies (1:100 dilution). All antibodies were matched with their correct fluorescent-conjugated isotype control.

*Transwell assay*: Cell culture inserts were put into the cell plate. RAW264.7 cells were added into cell culture inserts (polycarbonate membrane, 5.0 µm pore size, 6.5 mm membrane diameter, 0.33 cm surface area, upper chambers). Meanwhile, the plate (lower chambers) wells were filled with CXCL1. After four hours, cell culture inserts were removed, and cells on the upper side of the membrane were removed.

*Histology staining*: hearts were harvested immediately after mice were euthanized by CO_2_, and the tissue was fixed by 4% paraformaldehyde overnight at 4 °C. Fixed cardiac tissues were cryoprotected by soaking in 30% sucrose for 2-4 h at room temperature. The tissues were embedded in an optimal cutting temperature (OCT) compound (SAKURA, Tissue-Tek). A cryostat cut six micrometers of cryo-sections (Leica, CM3050). Masson staining (G1343-7, Solarbio, China) was a cardiac extracellular matrix remodeling marker for interstitial collagen fiber accumulation. The ratio of interstitial fibrosis to the total left ventricular area was calculated from 15 randomly selected microscopic sidles in individual sections per heart using a camera attached to a Leica DM2000 microscope, with images further analyzed by ImageJ, excluding coronary vessels and perivascular regions.

*Dot blot assays*: Total RNA from PBMCs of patients and RAW264.7 cells was extracted using TRIzol reagent (Invitrogen, Carlsbad, USA). The RNAs (200, 100 and 50 ng) were double diluted, denatured by heating at 95°C for 5 min and chilled on ice immediately. The RNAs were then spotted onto nitrocellulose membranes (Solarbio, Beijing, China). Then, the membranes were ultraviolet (UV) crosslinked, blocked, and incubated with an m^6^A-specific antibody (Huabio, Hangzhou, HA601049). The other membrane was stained with methylene blue as a loading control.

*Wound healing*: After stimulation with LPS for 24 h, the cell monolayer was scratched with a sterile pipette tip and washed with PBS. The scratch wound areas were observed at 0 and 24 h.

*mRNA decay analysis*: RAW264.7 cells were treated with actinomycin D at a final concentration of 2.5 μg/mL for 0, 0.5, 1, 2, and 3 h. Total RNA was extracted at the indicated time points for reverse transcription and qRT-PCR. The mRNA decay rate was normalized to that at 0 h.

*ChIP Assay*: Cells were crosslinked in 1% formaldehyde and quenched by the addition of 125 mM glycine, scraped, and collected by centrifugation, and then washed twice with cold phosphate-buffered saline. Cells were lysed from 1 plate (10 cm in diameter) with 1 mL sodium dodecyl sulfate lysis buffer containing protease inhibitor cocktail (GRF101; Yamei, Shanghai, China), by resuspension the pellet and pipetting up and down several times in a microcentrifuge tube. Crude chromatin preparations were sonicated on ice to yield DNA fragments of 150-300 bp in length and precleared with salmon sperm DNA-treated protein A/G agarose beads (30 mL, P2080S; Beyotime, Shanghai, China). The mixture of precleared chromatin preparations and 2 mg of primary antibody p65 (ab16502; Abcam, USA) were incubated overnight at 4 ℃. A negative control was included with normal IgG. Protein A/G agarose beads were added to capture the immunoprecipitated chromatin complexes. Finally, DNA fragments were released from the immunoprecipitated complexes by reverse cross-linking at 65 C for 1 hour, and quantitative real-time PCR was used to quantify the fragments of target gene promoters with specific primers (**Table S4**) using purified immunoprecipitated DNA as the template at 0 h.

*MeRIP-qPCR for Detection of m^6^A*: Total RNA was isolated and chemically fragmented (0.1 M Tris-HCl, pH = 7.0; 0.1 M EDTA, pH = 8.0) to approximately 200 nt in size, ethanol-precipitated and purified using the EasyPure RNA Purification Kit (ER701-01; TransGen, Beijing, China). 40 mg fragmented total RNA was precleared with protein A/G agarose beads (30 mL, P2080S; Beyotime, Shanghai, China) supplemented with 40U RNase inhibitor overnight at 4 ℃. The mixture and 2 mg m^6^A antibody (ab16502; Abcam) were incubated overnight at 4 ℃. A negative control was included with normal IgG (2729S; Cell Signaling Technology). Protein A/G agarose beads were added to capture the immunoprecipitated complexes. RNA was eluted from the beads by incubation in 300 mL of elution buffer (5 mM Tris-HCl, pH = 7.4; 1 mM EDTA, pH = 8.0 and 0.05% sodium dodecyl sulfate) with 20 mg of proteinase K for 1 hour at 60 ℃. Following phenol extraction and ethanol precipitation, the input and m^6^A-enriched RNA were reversely transcribed with random hexamers, and the enrichment was determined by qPCR. The primers used to detect m^6^A-enriched gene mRNA are shown in **Table S4**.

*Erythrocyte microvesicles (MVs) collection* 59-62: Mice blood was obtained via cardiac puncture. Erythrocytes were prepared by centrifugation of whole blood at 400× g in HEPES buffer with EGTA for 3 min at room temperature. As indicated, washed erythrocytes were resuspended in HEPES buffer with EGTA, and adjusted to 0.5 × 10^9^ cells/mL (corresponding to Hematocrit 4.0-4.5%). The top 1/4th layer was carefully discarded in each wash using a glass Pasteur pipette to remove residual leukocytes and platelets. The hematological counter controlled the main blood parameters (red blood cell count and mean cell volume (MCV)). Oxidative stress was induced by tert-butyl hydroperoxide (t-BOOH, 2 mM). Calcium stress, as increased intracellular Ca^2+^ concentration, was inducted by calcium ionophore A23187 (1 µM) in HEPES buffer with 2 mM calcium. Then the samples were gently centrifuged (50 × g, 7min) and the supernatant was collected for future analysis. Erythrocyte supernatant was centrifuged at 20,000× g for 30 min to separate free Hb and pellet-containing MVs. 50 kDa Millipore ultrafiltration tubes were used for the separation of MVs.

*Isolation of MVs from mice whole blood:* mice whole blood was collected in an EDTA/heparin Vacutainer and centrifuged for 5 minutes at 1500g to obtain plasma. To deplete the plasma of platelets and large membrane fragments, it was further centrifuged for 20 minutes at 1500g. MVs were then pelleted by centrifugation at 20,000g for 30 minutes. The pelleted MVs were washed with calcium-free Ringer's solution, resuspended in regular Ringer's solution, and quantified using dynamic light scattering (DLS).

*Fabrication of drug*-*loaded bioengineered erythrocyte microvesicles (STM@MVnex-u)*: STM@MV*nex-u* was prepared through a three-step assembly. Briefly, to prepare STM@MV*nex-u* specifically, STM2457 (100 μL, 19 mg mL^-1^), Coumarin 6 (100 μL, 3.8 mg mL^-1^), EGCG (200 μL, 5 mg mL^-1^) and erythrocyte MVs (final concentration, 6×10^11^ mL^-1^) were successively added to PBS (2 mL) under probe sonication (Sonics Vibracell probe sonicator, Newtown, CT, USA) for 10 min, with 2 s on, 2 s off at 15% amplitude. The assembly was initiated by introducing FeCl_3_·6H2O (64 μL, 5 mg mL^-1^) into the solution, and finally, PEG (200 μL, 20 mg mL^-1^) was added. STM@MV*nex-u* were obtained and stabilized by stirring the mixture for 10 min at room temperature. Finally, STM@MV*nex-u* were standardized using an Avanti extruder, passing them through a filter membrane with a pore size of 250 nm to ensure uniformity. High-speed centrifugation (8000 g, 10 min) was applied to remove excess materials, and the resultant STM@MV*nex-u* was finally dispersed in buffer solution for future use. STM@MV*nex* is made using the same process and concentrations but without ultrasound. STM@NP*nex-u* or MV*nex-u* follows the same method but omits erythrocyte MVs or STM2457, respectively.

*HPLC analysis of STM2457 content*: the STM2457 content in STM@MV*nex-u* or STM@MV*nex* was indirectly determined by measuring the unbound STM2457 remaining in the supernatant after assembly. Briefly, 0.8 mL of supernatant was collected and mixed with 0.2 mL of MeCN. HPLC analysis was conducted with an Agilent 1100 HPLC system (Palo Alto, CA, USA) equipped with an Ascentis C18 column (100 cm by 2.1 mm; particle size, 1.7 μm). The sample injection volume was 20 μL. The mobile phase was a 76:24 volume mixture of aqueous formic acid solution (0.12%) and MeCN and eluted at a 0.6 mL/min flow rate. The column temperature was maintained at 35°C. STM2457 was detected at a wavelength of 254 nm. STM2457 was dissolved in the mobile phase to 200 to 1000 μg/mL and analyzed in the same condition to build a calibration curve. The STM2457 loading capacity was calculated by subtracting the unbound STM2457 from the STM2457 feed. The STM2457 content was calculated as the STM2457 amount divided by the nanoparticle mass.

*Animal study design*: Sample sizes are reported in Figure Legend. The animal studies were performed on different models (n ≥ 4 biological replicates for each group). The sample size for the treatment experiments was determined mainly by referring to 10.1038/nature22076. No statistical methods were used to pre-determine sample sizes. No data were excluded from this research. Data presented were repeated at least three times (biological replicates) and the successful attempts at replication were confirmed. Animal groups were randomized by body weight. Animals were randomly allocated to each experimental group, but mice in the sham-operated group were additionally labeled and individually placed in a group.

*Cardiac fibrosis and remodeling model of mice*: Long-term Ang II infusion established a cardiac fibrosis model. 8- to 10-week-old mice were implanted with osmotic minipumps (ALZET; Durect Corp) containing Ang II (1,000 ng/kg/min, T7040, Targetmol, Shanghai, China). These osmotic minipumps were implanted subcutaneously in mice under anesthesia with 2% isoflurane. These mice were subjected to Ang II infusion for at least 4 weeks until the mice were sacrificed. The treatment group of experimental mice received injections of 2×10^11^ of STM@MV*nex-u* at three-day intervals for five injections. Meanwhile, the control group was administered an equivalent dosage of 2×10^11^ MVs or 0.07 mg of STM2457.

*Blood collection and processing for flow cytometry*: Mouse whole blood was collected by cardiac puncture using a heparin-precoated syringe and stored in BD Microtainer blood collection tubes at 4°C before use. The tubes were kept at room temperature for at least 90 minutes and then centrifuged (3,000 r.p.m. for 10 minutes at room temperature) to obtain serum. For flow cytometry, whole blood was diluted 1:10 in 1 × ACK Lysing Buffer (Thermofisher) and incubated at room temperature for 30 minutes on a rotator. Then, the cells were washed and 1×10^6^ cells were resuspended in 50 μL PBS containing 1% BSA and 2 mM sodium azide.

*Measurement of cardiac function by echocardiography*: echocardiography was performed on a VisualSonics Vevo 3100 Imaging System with a 40 MHz MicroScan transducer. Echocardiography was performed blinded to all groups. Mice were anesthetized with isoflurane (2.5% for induction and 0.5% for maintenance). Left ventricle end-diastolic and end-systolic chamber dimensions were measured from the 2-D short-axis under M-mode tracings at the papillary muscle level. Left ventricle functional parameters such as the percentage of fractional shortening (FS%) and ejection fraction (EF%) were calculated using the above primary measurements and accompanying software.

*Iron Analysis:* liver tissues were subjected to digestion in an acid solution comprising 10% trichloroacetic acid and 3 M HCl, maintained at 65°C for 20 hours. The determination of non-heme iron concentrations in both liver tissue and serum was performed utilizing bathophenanthrolinedisulfonic acid (BPS)-based colorimetry with background correction.

*Ferritin Analysis:* serum samples were obtained from blood specimens. Liver tissues were homogenized in RIPA buffer at a dilution ratio of 1:10 (w/v). The resulting lysate was centrifuged at 16,000×g for 6 minutes at 4°C, yielding a supernatant. Both serum and tissue supernatant were appropriately diluted using diluent buffer and subjected to a mouse ferritin ELISA assay (Abcam ab157713).

All procedures followed protocols approved by the West China Second University Hospital, Sichuan University, Animal Care and Use Committee. All animal experiments were performed in male mice to avoid possible shielding of estrogens.

*Statistical analyse*s: all the experiments were repeated at least three times. All statistical analyses were performed using GraphPad Prism 8. All data are presented as mean±SD. Student's t-tests, one-way ANOVA, two-way ANOVA with Tukey's HSD analysis, or Mann-Whitney U-tests were used to determine statistical significance. The number of antigen-positive cells in each section was calculated based on the mean number of more than three fields in one section. The Mantel-Cox test was used to analyze Kaplan- Meier survival curves. Asterisks represent different levels of significance; *P < 0.05 **P < 0.01 ***P < 0.001 ****P < 0.0001.

## Supplementary Material

Supplementary figures and tables.

## References

[1] Honold L, Nahrendorf M (2018). Resident and monocyte-derived macrophages in cardiovascular disease. Circ Res.

[2] Ginhoux F, Jung S (2014). Monocytes and macrophages: developmental pathways and tissue homeostasis. Nat Rev Immunol.

[3] Sanin DE, Ge Y, Marinkovic E, Kabat AM, Castoldi A, Caputa G (2022). A common framework of monocyte-derived macrophage activation. Sci Immunol.

[4] Yap J, Irei J, Lozano-Gerona J, Vanapruks S, Bishop T, Boisvert WA (2023). Macrophages in cardiac remodelling after myocardial infarction. Nat Rev Cardiol.

[5] Liu J, Dou X, Chen C, Chen C, Liu C, Xu MM (2020). N (6)-methyladenosine of chromosome-associated regulatory RNA regulates chromatin state and transcription. Science.

[6] Kumari R, Ranjan P, Suleiman ZG, Goswami SK, Li J, Prasad R (2022). mRNA modifications in cardiovascular biology and disease: with a focus on m6A modification. Cardiovasc Res.

[7] Berulava T, Buchholz E, Elerdashvili V, Pena T, Islam MR, Lbik D (2020). Changes in m6A RNA methylation contribute to heart failure progression by modulating translation. Eur J Heart Fail.

[8] Zheng Y, Li Y, Ran X, Wang D, Zheng X, Zhang M (2022). Mettl14 mediates the inflammatory response of macrophages in atherosclerosis through the NF-κB/IL-6 signaling pathway. Cell Mol Life Sci.

[9] Jian D, Wang Y, Jian L, Tang H, Rao L, Chen K (2020). METTL14 aggravates endothelial inflammation and atherosclerosis by increasing FOXO1 N6-methyladeosine modifications. Theranostics.

[10] Du J, Liao W, Liu W, Deb DK, He L, Hsu PJ (2020). N(6)-adenosine methylation of Socs1 mRNA is required to sustain the negative feedback control of macrophage activation. Dev Cell.

[11] Dubey PK, Patil M, Singh S, Dubey S, Ahuja P, Verma SK (2022). Increased m6A-RNA methylation and FTO suppression is associated with myocardial inflammation and dysfunction during endotoxemia in mice. Mol Cell Biochem.

[12] Zhu X, Tang H, Yang M, Yin K (2023). N6-methyladenosine in macrophage function: a novel target for metabolic diseases. Trends Endocrinol Metab.

[13] Jiang X, Liu B, Nie Z, Duan L, Xiong Q, Jin Z (2021). The role of m6A modification in the biological functions and diseases. Signal Transduct Target Ther.

[14] Feng Y, Dong H, Sun B, Hu Y, Yang Y, Jia Y (2021). METTL3/METTL14 Transactivation and m(6)A-dependent TGF-β1 translation in activated kupffer cells. Cell Mol Gastroenterol Hepatol.

[15] Zhang X, Li X, Jia H, An G, Ni J (2021). The m(6)A methyltransferase METTL3 modifies PGC-1α mRNA promoting mitochondrial dysfunction and oxLDL-induced inflammation in monocytes. J Biol Chem.

[16] Lim GB (2022). Macrophages and neutrophils modulate arrhythmia risk after myocardial infarction. Nat Rev Cardiol.

[17] Huang JS, Chen Q, Chen LW, Kuo YR, Hong ZN, Cao H (2019). Changes in the levels of inflammatory markers after transthoracic device closure of ventricular septal defects in pediatric patients. J Cardiothorac Surg.

[18] Dzeshka MS, Lip GY, Snezhitskiy V, Shantsila E (2015). Cardiac fibrosis in patients with atrial fibrillation: mechanisms and clinical implications. J Am Coll Cardiol.

[19] Yankova E, Blackaby W, Albertella M, Rak J, De Braekeleer E, Tsagkogeorga G (2021). Small-molecule inhibition of METTL3 as a strategy against myeloid leukaemia. Nature.

[20] Usman WM, Pham TC, Kwok YY, Vu LT, Ma V, Peng B (2018). Efficient RNA drug delivery using red blood cell extracellular vesicles. Nat Commun.

[21] Tang TT, Wang B, Wu M, Li ZL, Feng Y, Cao JY (2020). Extracellular vesicle-encapsulated IL-10 as novel nanotherapeutics against ischemic AKI. Sci Adv.

[22] Zhang J, Ji C, Zhang H, Shi H, Mao F, Qian H (2022). Engineered neutrophil-derived exosome-like vesicles for targeted cancer therapy. Sci Adv.

[23] Cheng G, Li W, Ha L, Han X, Hao S, Wan Y (2018). Self-Assembly of extracellular vesicle-like metal-organic framework nanoparticles for protection and intracellular delivery of biofunctional proteins. J Am Chem Soc.

[24] Noubouossie DF, Henderson MW, Mooberry M, Ilich A, Ellsworth P, Piegore M (2020). Red blood cell microvesicles activate the contact system, leading to factor IX activation via 2 independent pathways. Blood.

[25] Li X, La Salvia S, Liang Y, Adamiak M, Kohlbrenner E, Jeong D (2023). Extracellular vesicle-encapsulated adeno-associated viruses for therapeutic gene delivery to the heart. Circulation.

[26] Ding J, Lu G, Nie W, Huang LL, Zhang Y, Fan W (2021). Self-activatable photo-extracellular vesicle for synergistic trimodal anticancer therapy. Adv Mater.

[27] Liang J, Zhu F, Cheng K, Ma N, Ma X, Feng Q (2023). Outer membrane vesicle-based nanohybrids target tumor-associated macrophages to enhance trained immunity-related vaccine-generated antitumor activity. Adv Mater.

[28] Yong T, Wei Z, Gan L, Yang X (2022). Extracellular-vesicle-based drug delivery systems for enhanced antitumor therapies through modulating the cancer-immunity cycle. Adv Mater.

[29] Théry C, Ostrowski M, Segura E (2009). Membrane vesicles as conveyors of immune responses. Nat Rev Immunol.

[30] Hattangadi SM, Lodish HF (2007). Regulation of erythrocyte lifespan: do reactive oxygen species set the clock?. J Clin Invest.

[31] O'Dea KP, Tan YY, Shah S, B VP, K CT, Wilson MR (2020). Monocytes mediate homing of circulating microvesicles to the pulmonary vasculature during low-grade systemic inflammation. J Extracell Vesicles.

[32] Gao Z, Wang N, Ma Y, Sun H, Li M, Dai Y (2024). Targeting neutrophils potentiates hitchhiking delivery of drugs and agonists for postsurgical chemo-immunotherapy. Nano Today.

[33] Nguyen PQ, Courchesne ND, Duraj-Thatte A, Praveschotinunt P, Joshi NS (2018). Engineered living materials: prospects and challenges for using biological systems to direct the assembly of smart materials. Adv Mater.

[34] Xie J, Lee S, Chen X (2010). Nanoparticle-based theranostic agents. Adv Drug Deliv Rev.

[35] Guo J, Tardy BL, Christofferson AJ, Dai Y, Richardson JJ, Zhu W (2016). Modular assembly of superstructures from polyphenol-functionalized building blocks. Nat Nanotechnol.

[36] Dai Y, Yang Z, Cheng S, Wang Z, Zhang R, Zhu G (2018). Toxic reactive oxygen species enhanced synergistic combination therapy by self-assembled metal-phenolic network nanoparticles. Adv Mater.

[37] Ganea G, Cinteză EE, Filip C, Iancu MA, Balta MD, Vătășescu R (2023). Postoperative cardiac arrhythmias in pediatric and neonatal patients with congenital heart disease-a narrative review. Life (Basel).

[38] Wang L, Zhang YL, Lin QY, Liu Y, Guan XM, Ma XL (2018). CXCL1-CXCR2 axis mediates angiotensin II-induced cardiac hypertrophy and remodelling through regulation of monocyte infiltration. Eur Heart J.

[39] Gordon S (2012). Targeting a monocyte subset to reduce inflammation. Circ Res.

[40] Guo Q, Furuta K, Lucien F, Gutierrez Sanchez LH, Hirsova P, Krishnan A (2019). Integrin β(1)-enriched extracellular vesicles mediate monocyte adhesion and promote liver inflammation in murine NASH. J Hepatol.

[41] Kong P, Christia P, Frangogiannis NG (2014). The pathogenesis of cardiac fibrosis. Cell Mol Life Sci.

[42] Zeisberger SM, Odermatt B, Marty C, Zehnder-Fjällman AH, Ballmer-Hofer K, Schwendener RA (2006). Clodronate-liposome-mediated depletion of tumour-associated macrophages: a new and highly effective antiangiogenic therapy approach. Br J Cancer.

[43] Shinde AV, Humeres C, Frangogiannis NG (2017). The role of α-smooth muscle actin in fibroblast-mediated matrix contraction and remodeling. Biochim Biophys Acta Mol Basis Dis.

[44] Yunna C, Mengru H, Lei W, Weidong C (2020). Macrophage M1/M2 polarization. Eur J Pharmacol.

[45] Schmidl C, Renner K, Peter K, Eder R, Lassmann T, Balwierz PJ (2014). Transcription and enhancer profiling in human monocyte subsets. Blood.

[46] Thomsen T, Ayoub AB, Psaltis D, Klok HA (2021). Fluorescence-based and fluorescent label-free characterization of polymer nanoparticle decorated T cells. Biomacromolecules.

[47] Chen J, Pan S, Zhou J, Lin Z, Qu Y, Glab A (2022). Assembly of bioactive nanoparticles via metal-phenolic complexation. Adv Mater.

[48] Guo Y, Sun Q, Wu FG, Dai Y, Chen X (2021). Polyphenol-containing nanoparticles: synthesis, properties, and therapeutic delivery. Adv Mater.

[49] Ju Y, Dai Q, Cui J, Dai Y, Suma T, Richardson JJ (2016). Improving targeting of metal-phenolic capsules by the presence of protein coronas. ACS Appl Mater Interfaces.

[50] Zhao Z, Pan DC, Qi QM, Kim J, Kapate N, Sun T (2020). Engineering of living cells with polyphenol-functionalized biologically active nanocomplexes. Adv Mater.

[51] de Couto G (2019). Macrophages in cardiac repair: Environmental cues and therapeutic strategies. Exp Mol Med.

[52] Zhang Z, Dalan R, Hu Z, Wang JW, Chew NW, Poh KK (2022). Reactive oxygen species scavenging nanomedicine for the treatment of ischemic heart disease. Adv Mater.

[53] Haissaguerre M, Vigmond E, Stuyvers B, Hocini M, Bernus O (2016). Ventricular arrhythmias and the His-Purkinje system. Nat Rev Cardiol.

[54] Delvasto-Núñez L, Roem D, Bakhtiari K, van Mierlo G, Meijers JCM, Jongerius I (2022). Iron-driven alterations on red blood cell-derived microvesicles amplify coagulation during hemolysis via the intrinsic tenase complex. Thromb Haemost.

[55] Melgarejo E, Medina MA, Sánchez-Jiménez F, Urdiales JL (2009). Epigallocatechin gallate reduces human monocyte mobility and adhesion in vitro. Br J Pharmacol.

[56] Li Y, Karim MR, Wang B, Peng J (2022). Effects of green tea (-)-epigallocatechin-3-gallate (EGCG) on cardiac function - a review of the therapeutic mechanism and potentials. Mini Rev Med Chem.

[57] von Eckardstein A (2019). Iron in coronary heart disease-J-shaped associations and ambivalent relationships. Clin Chem.

[58] Li Y, Hua Y, Fang J, Wan C, Wang C, Zhou K (2015). Identification of risk factors for arrhythmia post transcatheter closure of perimembranous ventricular septal defect. J Invasive Cardiol.

[59] Nguyen DB, Ly TB, Wesseling MC, Hittinger M, Torge A, Devitt A (2016). Characterization of microvesicles released from human red blood cells. Cell Physiol Biochem.

[60] Lutz HU, Liu SC, Palek J (1977). Release of spectrin-free vesicles from human erythrocytes during ATP depletion. I. Characterization of spectrin-free vesicles. J Cell Biol.

[61] Jana S, Strader MB, Meng F, Hicks W, Kassa T, Tarandovskiy I (2018). Hemoglobin oxidation-dependent reactions promote interactions with band 3 and oxidative changes in sickle cell-derived microparticles. JCI Insight.

[62] Dinkla S, Brock R, Joosten I, Bosman GJ (2013). Gateway to understanding microparticles: standardized isolation and identification of plasma membrane-derived vesicles. Nanomedicine (Lond).

