# Supplementary material for: Deciphering m6A methylation in monocyte-mediated cardiac fibrosis and monocyte-hitchhiked erythrocyte microvesicle biohybrid therapy
Source: Theranostics. 2024 Jun 1;14(9):3486–508. doi: 10.7150/thno.95664 (PMC11209724; doi:10.7150/thno.95664)
Supplement: Supplementary file 1 — Supplementary figures and tables. [file thnov14p3486s1.pdf]

Supporting Information

Deciphering m^6A methylation in monocyte-mediated cardiac fibrosis and monocyte-hitchhiked erythrocyte microvesicle biohybrid therapy

Jiawen Li, Li Wei, Kaifeng Hu, Yunxiang He, Guidong Gong, Qisong Liu, Yue Zhang, Kaiyu Zhou, Junling Guo, Yimin Hua*, Jun Tang*. Yifei Li*

Corresponding author: Yimin Hua, Nathan_hua@163.com; Jun Tang, tj1234753@sina.com; Yifei Li, liyfwcsh@scu.edu.cn.

§ These authors contributed equally

The PDF file includes:

Figs. S1 to S27

Table S1 to S4

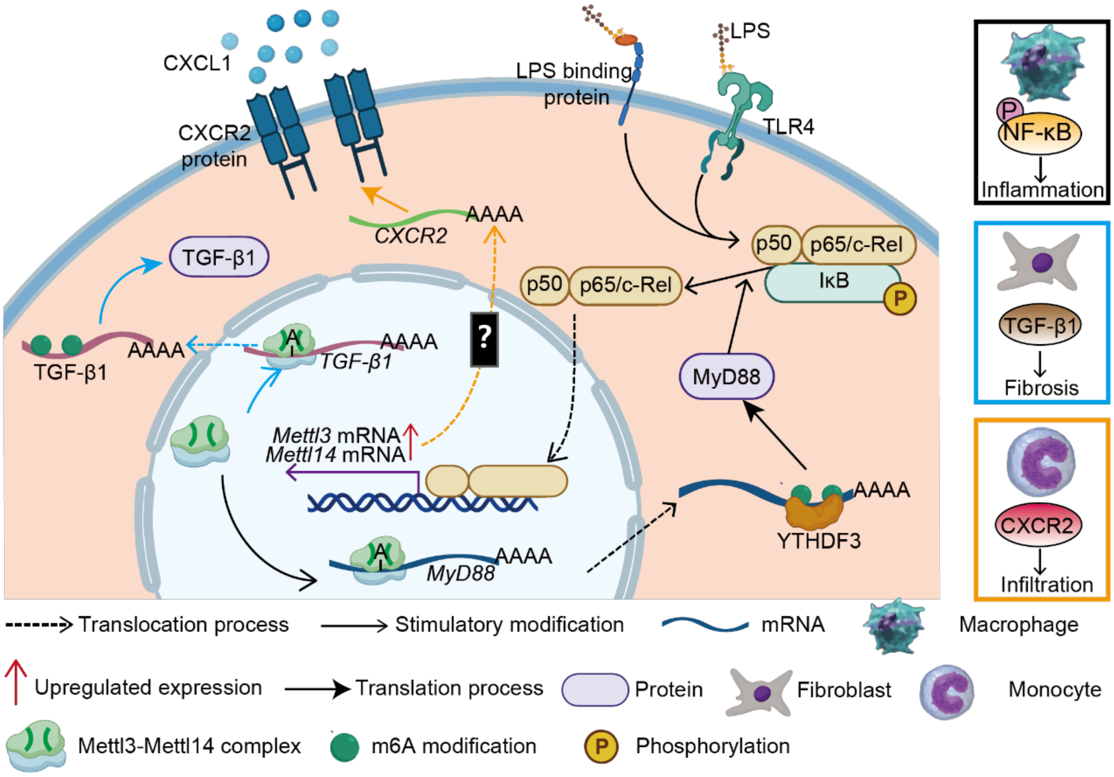

Figure S1: A working model for the interrelationship between monocyte inflammatory phenotypes, migration, and fibrosis with m^{6}A modification. m^{6}A modification enhances the stability of *MyD88* mRNA through recognition by YTHDF3, thereby strengthening the activation of the NF-κB pathway triggered by LPS. In turn, the NF-κB transcriptional activators act on the promoters of METTL3 and METTL14, enhancing their transcription. On another front, METTL3 and METTL14 promote the expression of m^{6}A-modified TGF-β1 mRNA directly, and CXCR2 expression indirectly.

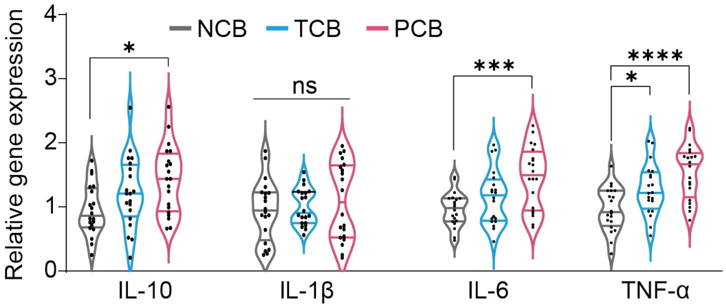

Figure S2. mRNA levels of anti-inflammatory cytokine of IL-10 and pro-inflammatory cytokines of IL-1β, IL-6 and TNF-a. n ≥ 16 per group. The data are expressed as the mean ± SD. P-values were determined by one-way ANOVA with Fisher's LSD post-hoc test. *, $P < 0.05$; **, $P < 0.01$; ***, $P < 0.001$; ****, $P < 0.0001$.

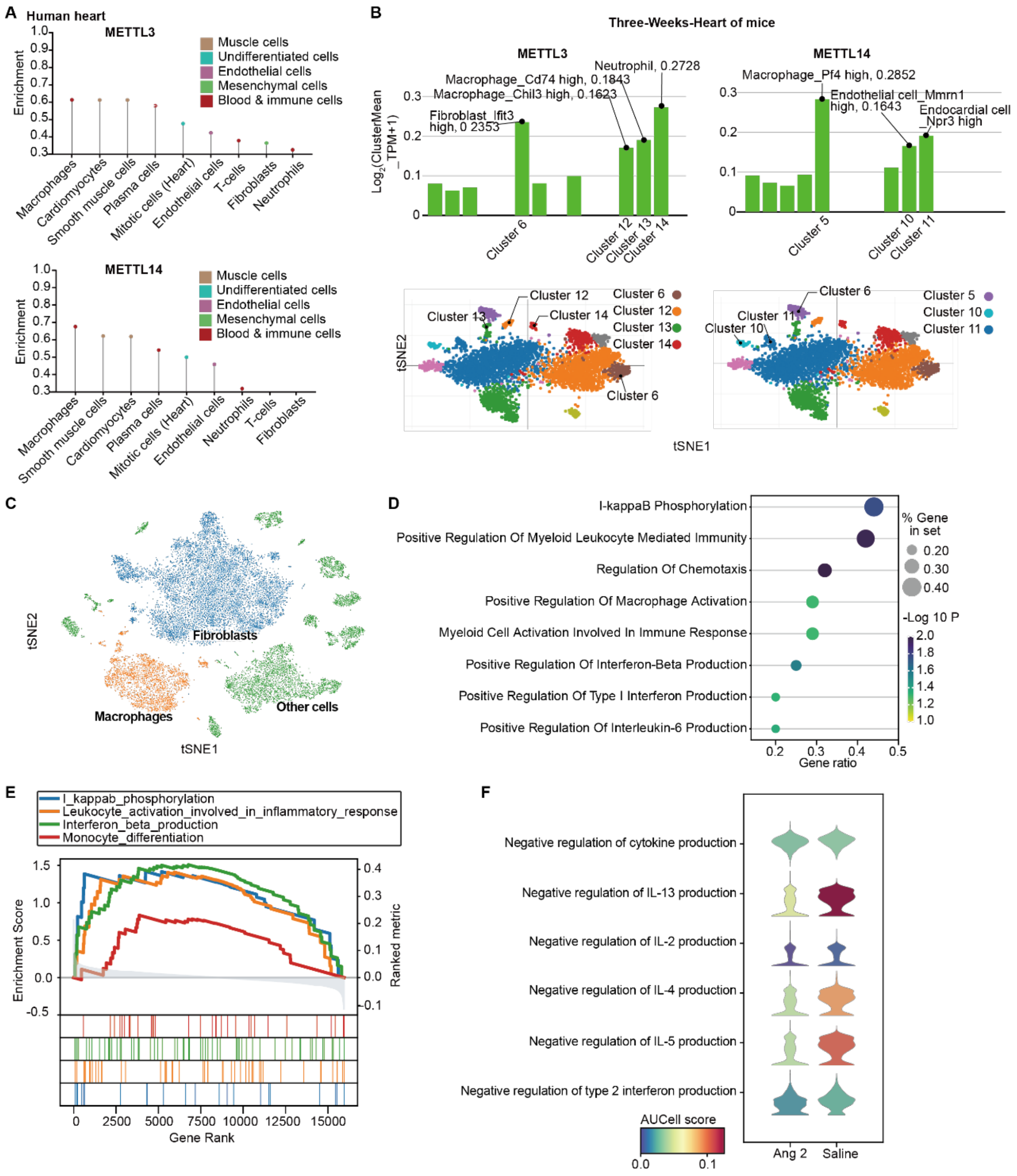

Figure S3 Single-cell RNA sequencing analysis of METTL3 and METTL14 in human and mouse heart cells following Ang II induction.

(**A**) Enrichment prediction score for METTL3 and METTL14 across each cell type profiled in human heart tissue according to The Human Protein Altas. This score calculates the mean correlation between METTL3/METTL14 and three reference transcripts representing each cell type. (**B**) t-SNE plot of single-cell RNA-seq data from 3-week-old mouse hearts. Analysis using the Mouse Cell Atlas reveals high METTL3 and METTL14 expression in cardiac macrophages, indicating significant transcript abundance in these cells. (**C**) UMAP mapping of single-cell RNA sequencing data from mouse hearts post-Ang II induction, displaying clusters of macrophages, fibroblasts, and other cell types. (**D**) GO-Term analysis shows significant enrichment of signaling pathways in macrophages before and after Ang II

induction, including I-κB phosphorylation, macrophage activation, chemotaxis, and the production of IL-1β, IFN-1, and IL-6. (**E**) GSEA highlights the trends in enriched pathways following Ang II induction. (**F**) AUCell analysis of gene sets in macrophages reveals enhanced activity levels post-Ang II induction, particularly in producing pro-inflammatory cytokines.

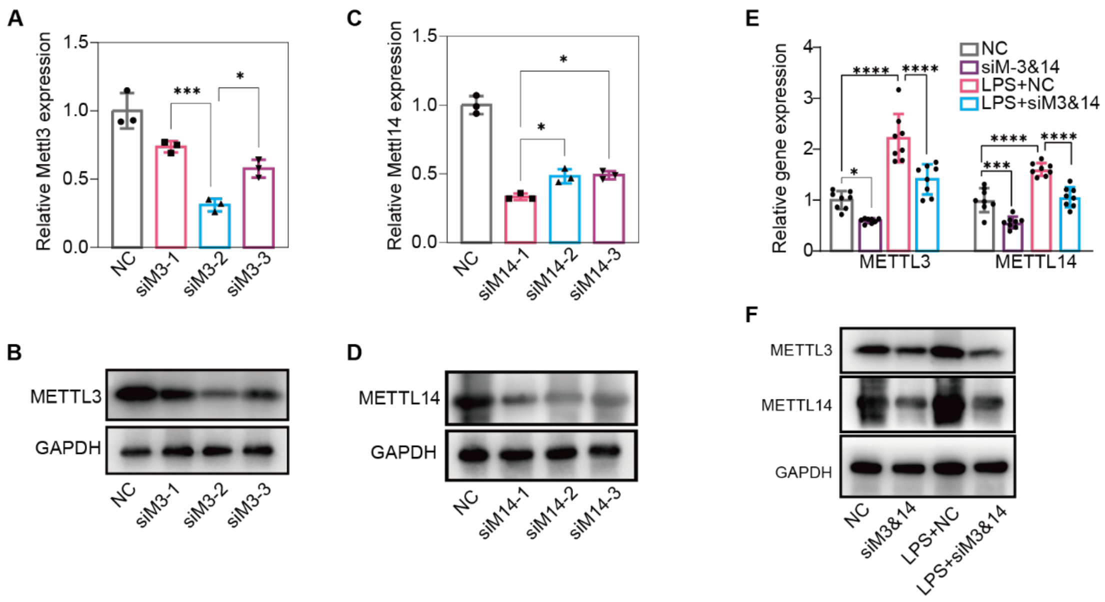

Figure S4 The efficiency of the siRNA-mediated downregulation of METTL3 and METTL14 was assessed at the mRNA and protein levels—Knockdown of siRNA for METTL3 and METTL14 with 3 target sites, respectively.

(**A-D**) Expression of the METTL3 (A and B) and METTL14 (C and D) mRNA and protein measured in RAW264.7 cells transfected with siRNA of METTL3 (siM3) or siRNA of METTL14 (siM14) using qRT-PCR and western blot. n = 3 per group. (**E** and **F**) Knockdown of METTL3 and METTL14 of siRNA (siM3-2, siM14-1) chosen according to results in (**A-D**), with or without treatment of LPS for BMDMs. BMDMs were harvested 24 hours after LPS treatment. mRNA levels (A) and protein contents (B) of METTL3 and METTL14 were examined by qRT-PCR and western blot. P-values were determined by one-way ANOVA with Fisher's LSD post-hoc test. *, $P < 0.05$; **, $P < 0.01$; ***, $P < 0.001$; ****, $P < 0.0001$.

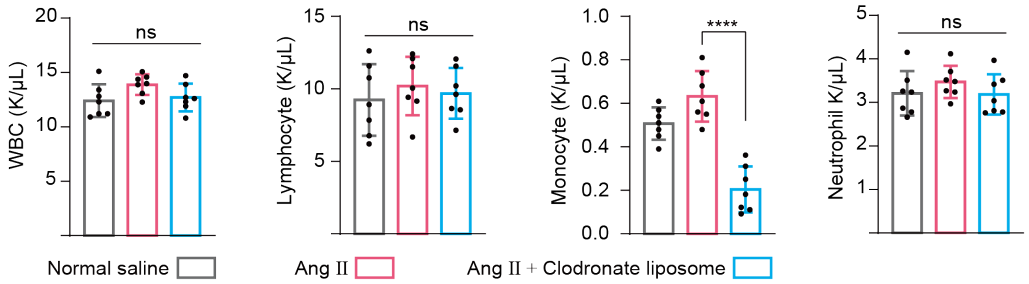

Figure S5 Complete blood counts, including leukocytes, lymphocytes, monocytes, and neutrophils, from mice with Ang II-induced myocardial fibrosis treated with clodronate liposomes were assessed using a hemocytometer. Normal saline-treated mice and Ang II-treated mice that did not receive clodronate liposome injections were used as controls. n = 7 per group. P-values were determined by one-way ANOVA with Fisher's LSD post-hoc test. *, P < 0.05; **, P < 0.01; ***, P < 0.001; ****, P < 0.0001.

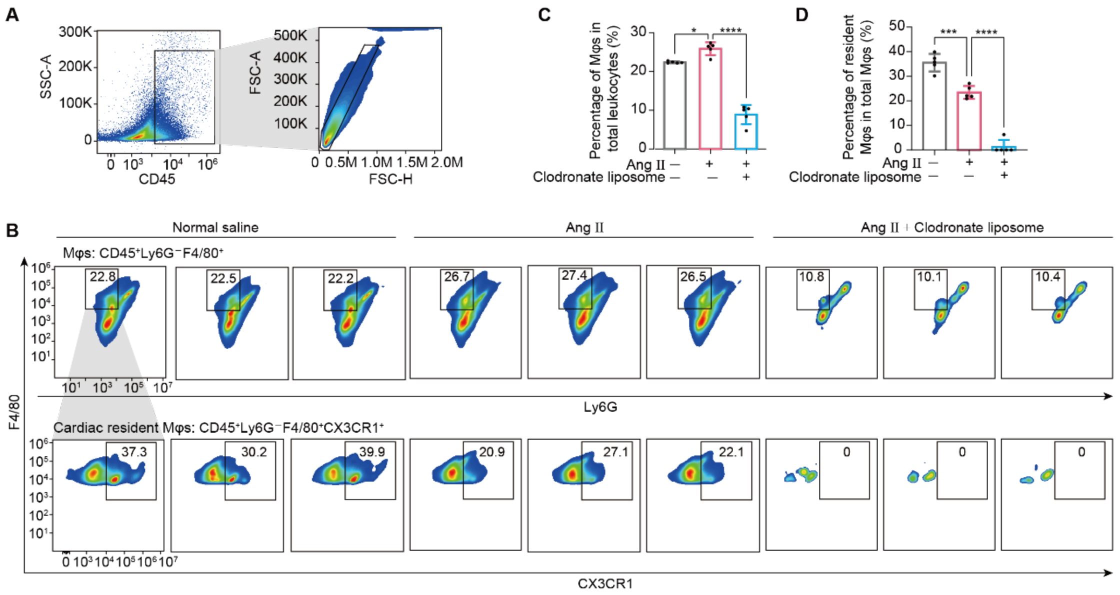

Figure S6 Effects of Clodronate Liposome on Mice with Ang II-Induced Myocardial Fibrosis were assessed by flow cytometry. Hearts were harvested and dissociated into single-cell suspensions 5 days post-injection. Controls included Ang II-treated mice that did not receive clodronate liposome injections and untreated mice.

(**A**) Flow cytometry gating strategy identified macrophages as $CD45^{+}Ly6G^{-}F4/80^{+}$ cells. (**B**) Representative dot plots of cardiac single-cell suspensions highlight total and resident macrophages ($CX3CR1^{+}$). (**C**) Flow cytometric quantification of macrophage percentages among leukocytes. (**D**) Quantitative analysis of resident macrophages as a percentage of total macrophages. Bar charts are presented as mean ± SD. P-values were determined by one-way ANOVA with Fisher's LSD post-hoc test. *, $P < 0.05$; **, $P < 0.01$; ***, $P < 0.001$; ****, $P < 0.0001$.

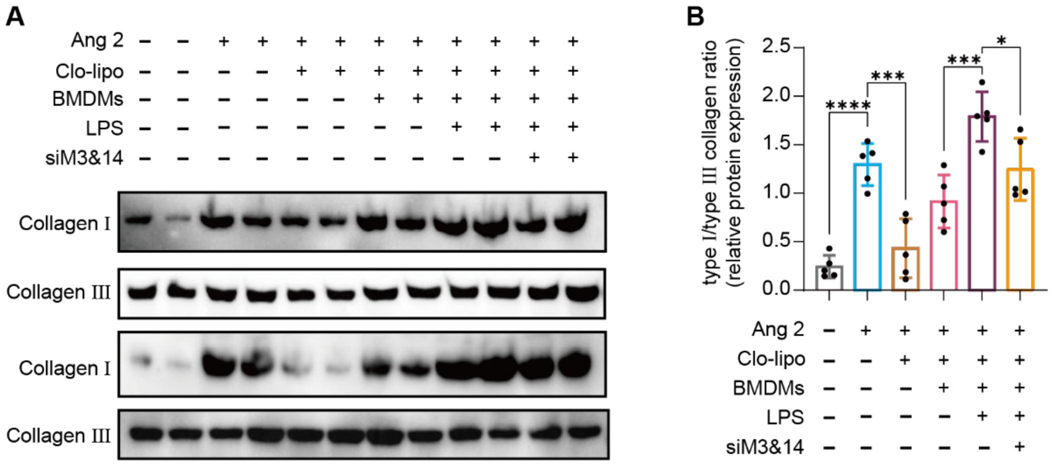

Figure S7 Identification of myocardial fibrosis types through the protein expression ratio of Type I to Type III collagen fibers. (A) Western blot analysis of Collagen I and Collagen III following treatment with Ang II, clodronate liposomes (Clo-lipo), the injection of in-vitro-cultured BMDMs that are either untreated, treated by LPS with or without transfection of siM3&14. (B) Quantitative analysis depicting the statistical evaluation of the ratio between Collagen I and Collagen III across the experimental groups detailed above. Bar charts are presented as mean ± SD. P-values were determined by one-way ANOVA with Fisher's LSD post-hoc test. *, $P < 0.05$; **, $P < 0.01$; ***, $P < 0.001$; ****, $P < 0.0001$.

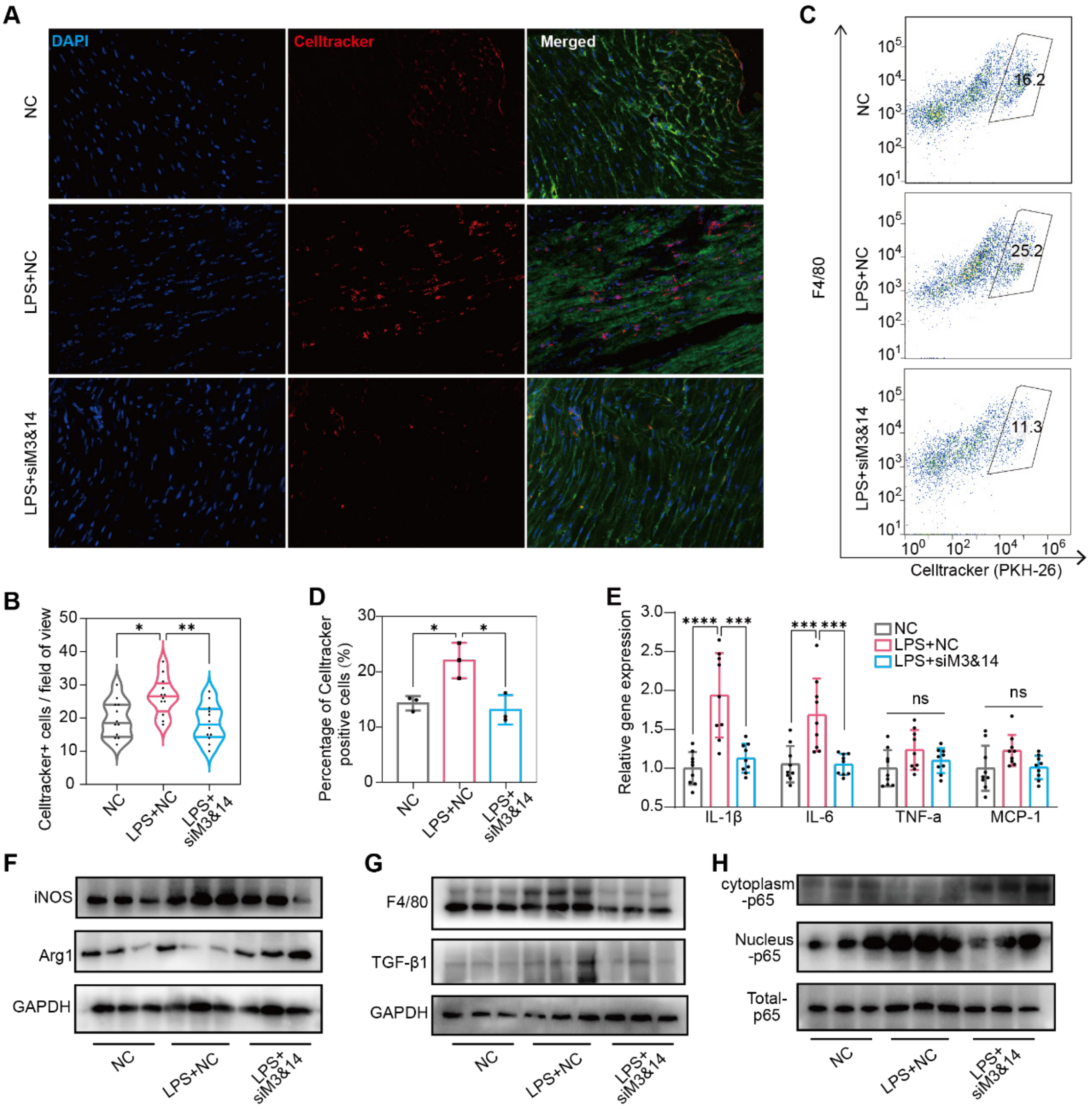

Figure S8 Knockdown of METTL3 and METTL14 in adoptively transferred BMDMs alters monocyte recruitment and macrophage polarization. Clodronate liposomes were used to deplete monocytes and macrophages after inducing myocardial fibrosis in mice with Ang II. Subsequently, exogenous BMDMs were injected to reconstruct the monocyte/macrophage system. Three groups of mice were injected with BMDMs which were intervened with siRNA-NC (NC group), LPS+siRNA-NC (LPS+NC group), and LPS+siM3&14, respectively.

(**A** and **B**) LSCM images of PKH-26 positive donor monocytes in hearts (A) and quantification of the number of the recruited monocytes (B). n = 12 per group. (**C**) Flow cytometry analysis of single-cell suspension of hearts 10 days after monocytes (prelabeled with PKH-26) injection. By forward scatter versus side scatter plots we exclude cell doublets and other types of cells with big morphological differences with macrophages, such as cardiomyocyte and endothelial cells. Cells displaying triple positivity for CD11b, F4/80, and PKH-26 were identified as donor cells. Gate shows the double positive population within the $CD11b^+$ population. The gating area was chosen based on the sample from untreated mice. (**D**) Quantifying the percentage of macrophages from donor BMDMs ($PKH\text{-}26^+$) of total

macrophages ($CD11b^{+}F4/80^{+}$) in hearts. The donor BMDMs were subjected to pre-treatment with NC, LPS+NC, or LPS+siM3&14. n = 3 per group. (**E**) qRT-PCR analysis of the mRNA levels of M1 macrophage markers, including IL-1b, IL-6, TNF-a and MCP-1 in the hearts. n = 9 per group. (**F**) Protein contents of iNOS (M1 macrophage marker) and Arg1 (M2 macrophage marker) of macrophage in the left ventricles 10 days after treatment. (**G**) Protein contents of F4/80 and TGF-β1 in the left ventricles 10 days after treatment. (**H**) The protein content of p65 in total lysate, cytoplasmic lysate, and nuclear lysate. P-values were determined by one-way ANOVA with Fisher's LSD post-hoc test. *, $P < 0.05$; **, $P < 0.01$; ***, $P < 0.001$; ****, $P < 0.0001$.

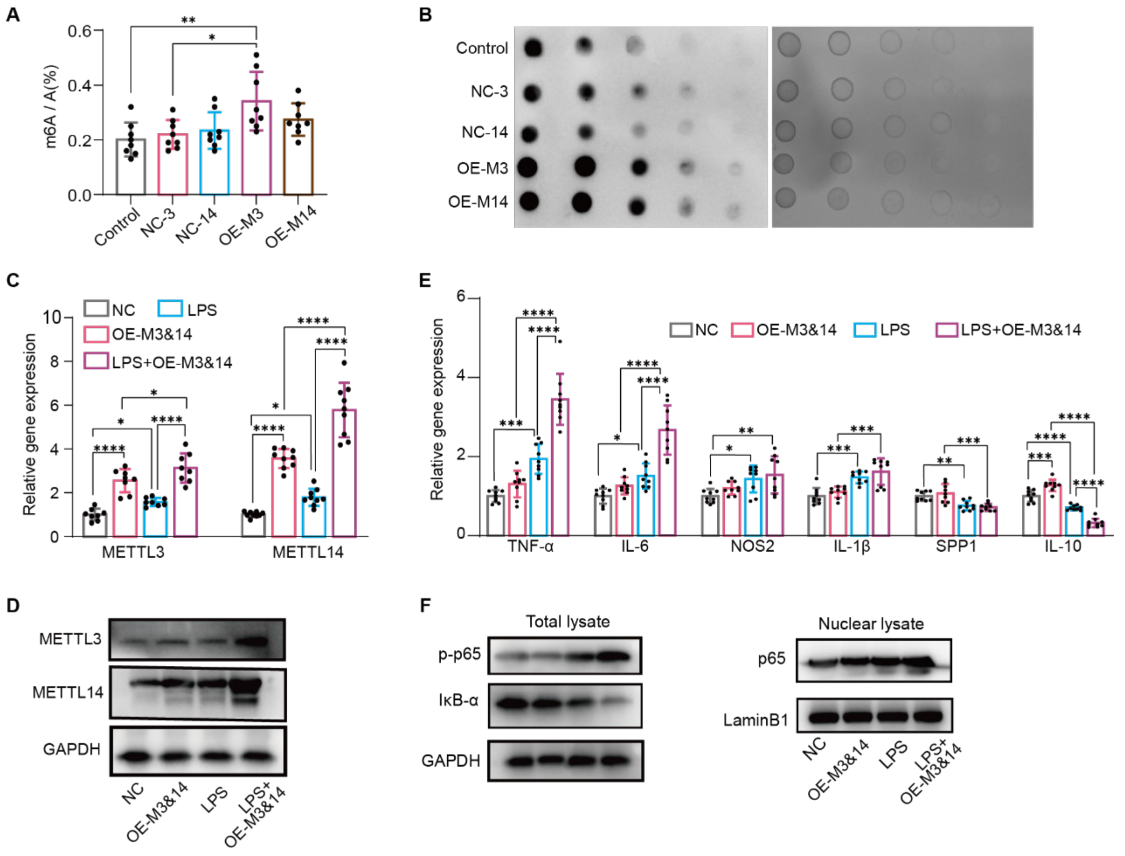

Figure S9 Enhancement of LPS-induced effects by overexpression of METTL3 and METTL14 on m^{6}A modification and inflammatory responses in RAW 264.7 cells.

(**A** and **B**) Global m^{6}A level of RAW 264.7 cells detected by colorimetric (A) and dot blot (B). Control, only transfection reagents; NC-3, control plasmid of *METTL3* overexpression plasmid; NC-14, control plasmid of *METTL14* overexpression plasmid; OE-M3, overexpression plasmid of *METTL3*; OE-M14, overexpression plasmid of *METTL14*. n = 8 per group. (**C** and **D**) Expression of METTL3 and METTL14 mRNA and protein in RAW264.7 cells. Groups include negative control (NC), cells transfected with overexpression plasmid of *METTL3* and *METTL14*. (OE-M3&14), LPS-treated cells (LPS), and LPS plus transfection group (LPS+OE-M3&14). n = 9 per group. (**E**) The mRNA levels of M1 markers, including TNF-α, IL-6, NOS2 and IL-1β, and M2 markers, including SPP1 and IL-10 of RAW264.7 cells after treatment with NC, OE-M3&14, LPS and LPS+ OE-M3&14. n = 9 per group. (**F**) Immunoblot for IκBα, and p-p65 (Ser536) of total lysate, p65 of nuclear lysates, which were extracted from the above cells. P-values were determined by one-way ANOVA with Fisher's LSD post-hoc test. *, $P < 0.05$; **, $P < 0.01$; ***, $P < 0.001$; ****, $P < 0.0001$.

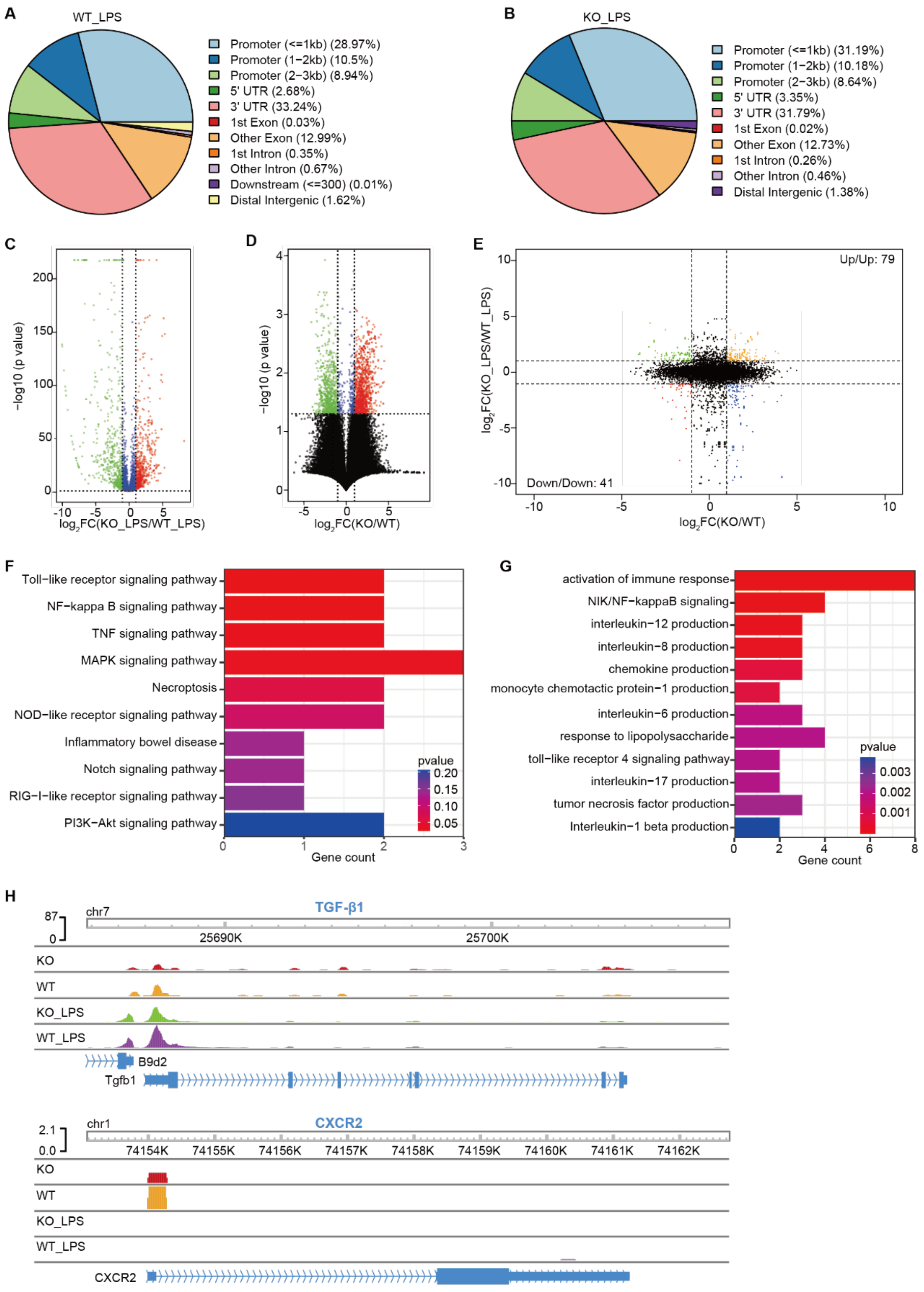

Figure S10. m^{6}A modification compared WT macrophages with METTL3-KO macrophages with or without LPS intervention by MeRIP-seq.

(**A** and **B**) Pie chart presenting fractions of m^{6}A peaks in different transcript segments of WT and METTL3-KO macrophages with LPS intervention. (**C** and **D**) RNA-seq volcano plots of WT and METTL3-KO macrophages with or without LPS intervention. (**E**) Quadrant_plots of differential transcripts. (**F**, **G**) Gene Ontology (GO) and Kyoto Encyclopedia of Genes and

Genomes (KEGG) enrichment analysis in the biological process and signaling pathway category of transcripts bearing m^6A modification for intersection transcripts down-regulated by knockdown of METTL3 with or without LPS [Down/Down in (E)]. (**H**) Read density in TGF-β1 and CXCR2 transcript.

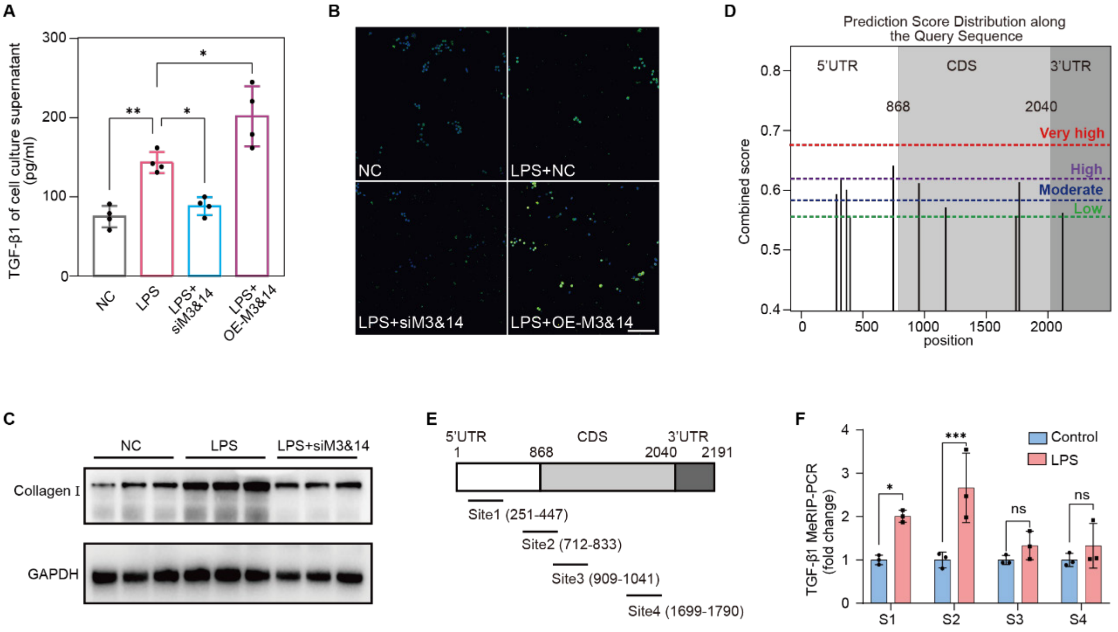

Figure S11. METTL3/METTL14-driven m^{6}A regulation of TGF-β1 and pro-fibrosis in LPS-treated RAW 264.7 cells

(**A**) TGF-β1 concentrations measured by ELISA in the supernatant of RAW264.7 cells treated with NC, LPS+NC, LPS+siM3&14 and LPS+OE-M3&14. n = 4 per group. (**B**) Immunofluorescence of TGF-β1 in RAW 264.7 cells treated with NC, LPS+NC, LPS +siM3&14 and LPS+OE-M3&14 in medium–added monensin (3μM) for 4 h before harvest of cells. (**C**) The collagen I protein levels in NIH-3T3 cells after stimulation with supernatant from above cells in (A). (**D**) Prediction sites of *TGF-β1* mRNA m^{6}A peaks as produced from SRAMP. (**E**) Schematic of *TGF-β1* mRNA segments. (**F**) MeRIP-qPCR was performed to detect the m^{6}A abundance of *TGF-β1* of 4 sites (F) in LPS-treated cells. n = 3 per group. P-values were determined by one-way ANOVA with Fisher's LSD post-hoc test. *, P < 0.05; **, P < 0.01; ***, P < 0.001; ****, P < 0.0001.

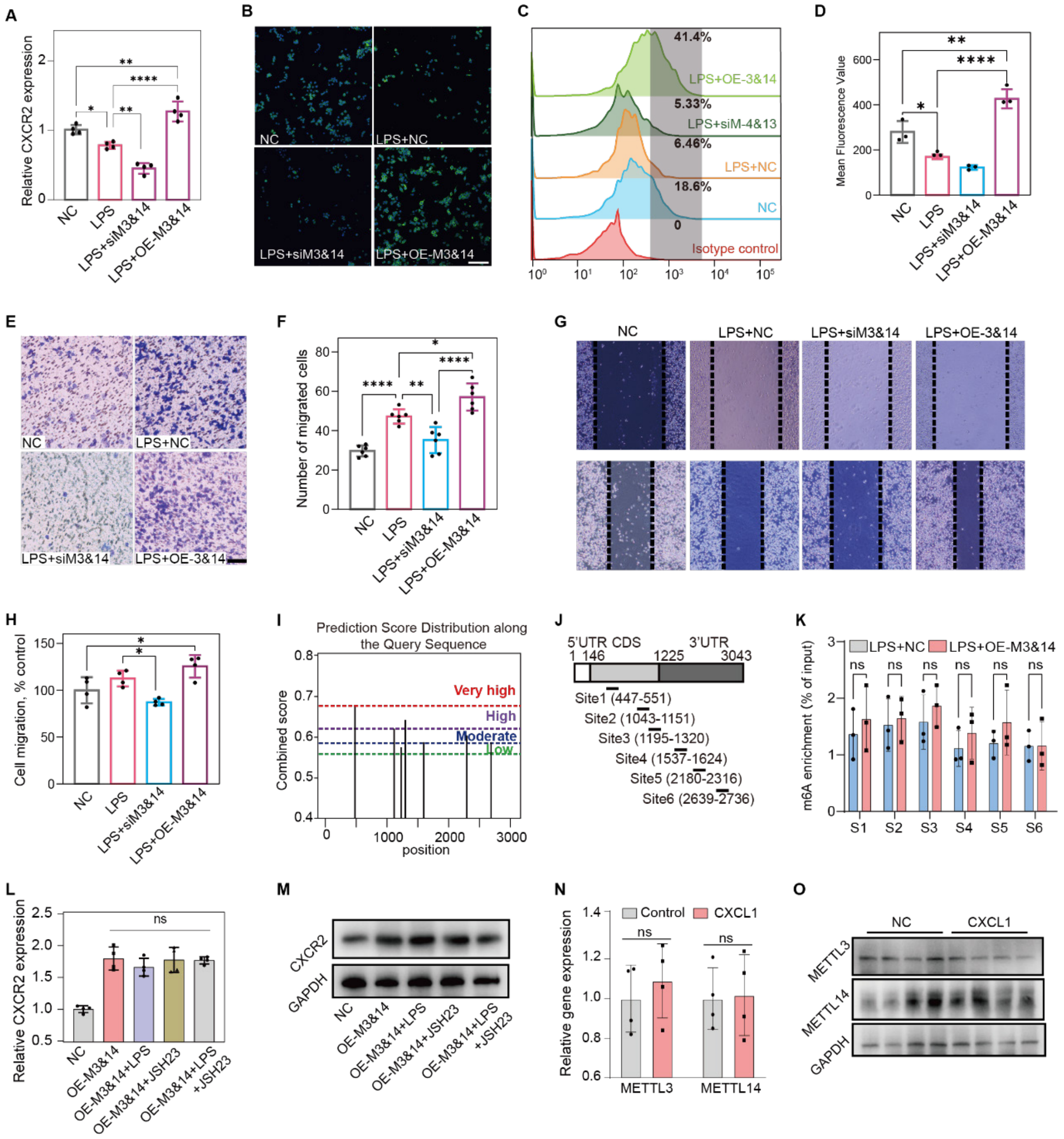

Figure S12. METTL3/METTL14 overexpression promotes CXCR2 expression in monocytes, which regulates chemotaxis and migration in vitro.

(**A**) mRNA and protein expression of CXCR2 measured by qRT-PCR and immunoblot in the above cells. RAW264.7 cells were transfected with NC, siM3&14, or OE-M3&14 before treatment with LPS (100 ng/ml). n = 4 per group. (**B**) Immunofluorescence of above cells. (**C**) Typical flow cytometry results of CXCR2 surface expression and percentage of CXCR2^{+} cells (**D**) Mean fluorescence values of CXCR2 in cells of (C). n = 3 per group. (**E** and **F**) Typical transwell images (E) and the chemotactic number (F) of the above cells toward CXCL1 in the lower chamber after 6 h. n = 6 per group. (**G** and **H**) Cell migration (G) and quantification (H) detected by wound healing assay. n = 4 per group. (**I**) Prediction sites of CXCR2 mRNA m^{6}A peaks as produced from SRAMP. (**J**) Schematic of CXCR2 mRNA segments. (**K**) MeRIP-PCR was performed to detect the m^{6}A abundance in LPS-treated cells with or without overexpression of METTL3 and METTL14. n = 3 per group. (**L** and **M**) qPCR and western blot were used to detect the mRNA expression and CXCR2 protein content after treatment with NC, OE-M3&14, OE-M3&14+LPS, OE-M3&14+JSH23, OE-

M3&14+LPS+JSH23. n = 4 per group. (**N** and **O**) qPCR and western blot were used to detect the METTL3 and METTL14 mRNA expression (N) and protein content (O) after treatment with CXCR2 activator CXCL1. n = 3 per group. P-values were determined by one-way ANOVA with Fisher's LSD post-hoc test. *, $P < 0.05$; **, $P < 0.01$; ***, $P < 0.001$; ****, $P < 0.0001$.

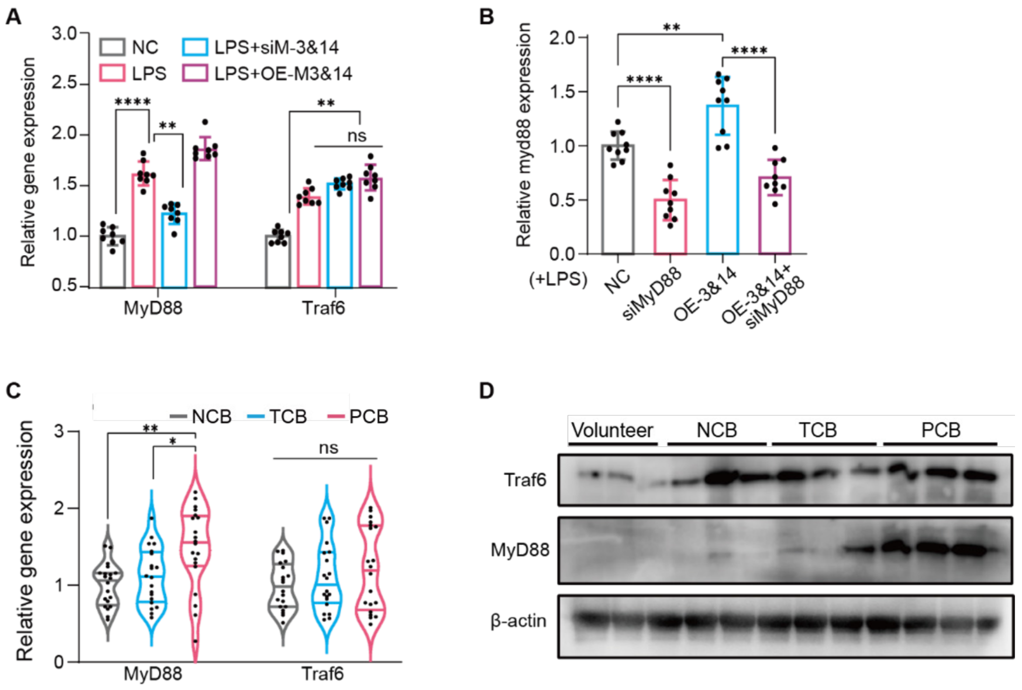

Figure S13. Differential expression of MyD88 and Traf6 in RAW 264.7 cells and PBMCs (**A**) The mRNA levels of *MyD88* and *Traf6* of RAW264.7 cells after treatment with NC, LPS+NC, LPS+ siM3&14, LPS+OE-M3&14. n = 8 per group. (**B**) The expression of *MyD88* mRNA was detected by qRT-PCR and normalized to GAPDH for RAW264.7 cells treated with NC, siMyD88, OE-M3&14, OE-M3&14+siMyD88. n = 9 per group. (**C**) mRNA expression of *MyD88* and *Traf6* of PBMC from patients. n = 20 per group. (**D**) Protein level of MyD88 and Traf6 in PBMCs from healthy volunteers and patients. P-values were determined by one-way ANOVA with Fisher's LSD post-hoc test. *, $P < 0.05$; **, $P < 0.01$; ***, $P < 0.001$; ****, $P < 0.0001$.

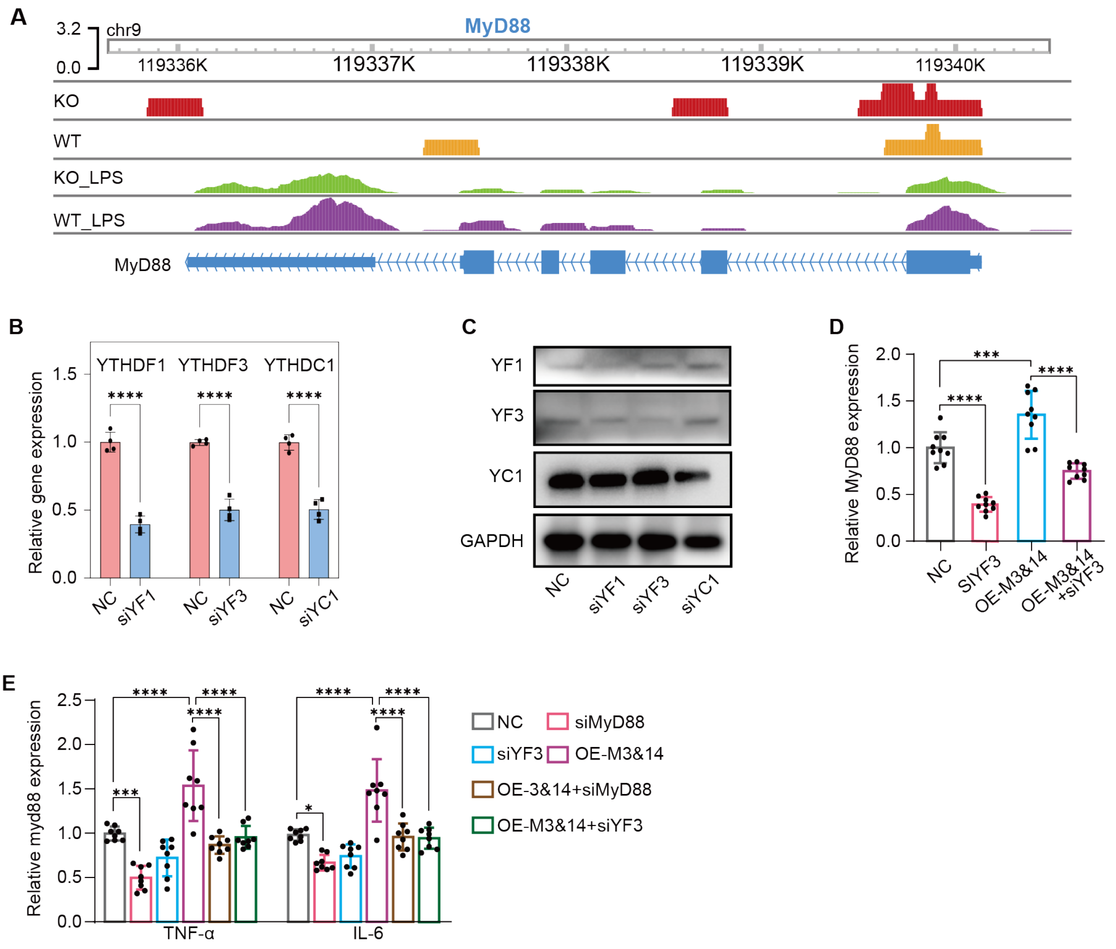

Figure S14. Impact of siYF3 and siMyD88 on METTL3/14-overexpressed RAW 264.7 cells

(**A**) Read density in *MyD88* transcript of WT macrophages and METTL3-KO macrophages with or without LPS intervention, assessed by MeRIP-seq. (**B**) Expression of the *YTHDF1*, *YTHDF3*, *YTHDC1* mRNA was measured in RAW264.7 cells transfected with siRNA of YTHDF1 (siYF1), YTHDF3 (siYF3) or YTHDC1 (siYC1) using qRT-PCR. n = 4 per group. (**C**) Immunoblot of the YTHDF1, YTHDF3, YTHDC1 was measured in RAW264.7 cells transfected with siYF1, siYF3 and siYC1. (**D**) The mRNA levels of *MyD88* of RAW264.7 cells transfected with NC, siYF3, OE-M3&14, OE-M3&14+siYF3 before LPS treatment. n = 9 per group. (**E**) The mRNA levels of TNF-a and IL-6 of RAW264.7 cells transfected with NC, siMyD88, siYF3, OE-M3&14, OE-M3&14+siMyD88, OE-M3&14+siYF3 before LPS treatment. n = 8 per group. P-values were determined by one-way ANOVA with Fisher's LSD post-hoc test. *, P < 0.05; **, P < 0.01; ***, P < 0.001; ****, P < 0.0001.

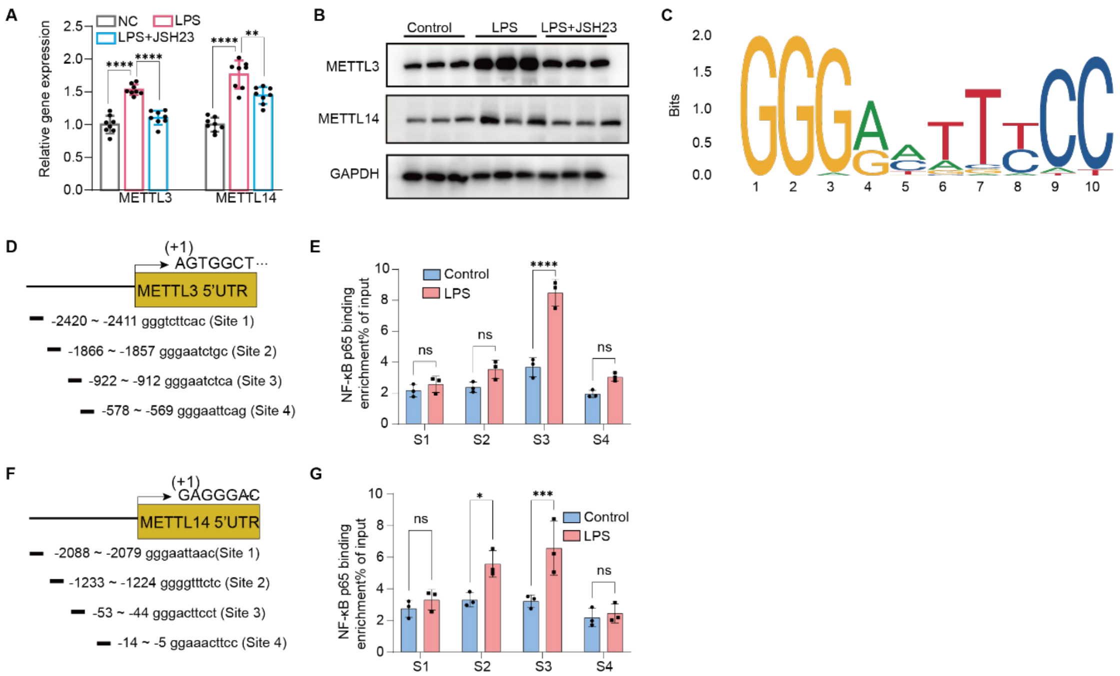

Figure S15. METTL3 and METTL14 genes are activated by NF-κB transcriptional activator p65.

(**A** and **B**) qPCR (A) and western blot (B) were used to detect the METTL3 and METTL14 mRNA and protein expression after treatment with LPS and NF-κB inhibitor JSH23. n = 8 per group. (**C**) Sequence logo representing consensus motif of DNA binding sites of NF-κB transcription factors according to JASPAR. (**D**) Schematic representation of NF-κB transcription factors binding sites of METTL3 promoter region predicted by EPD and JASPAR. (**E**) ChIP-PCR assay was used to measure the binding sites (S1, S2, S3, S4) of NF-kB on METTL3 with or without treatment of LPS. n = 3 per group. (**F**) Schematic representation of NF-κB transcription factors binding sites of METTL14 promoter region. (**G**) ChIP-PCR assay was used to measure the binding sites of NF-kB on METTL14. n = 3 per group. P-values were determined by one-way ANOVA with Fisher's LSD post-hoc test. *, P < 0.05; **, P < 0.01; ***, P < 0.001; ****, P < 0.0001.

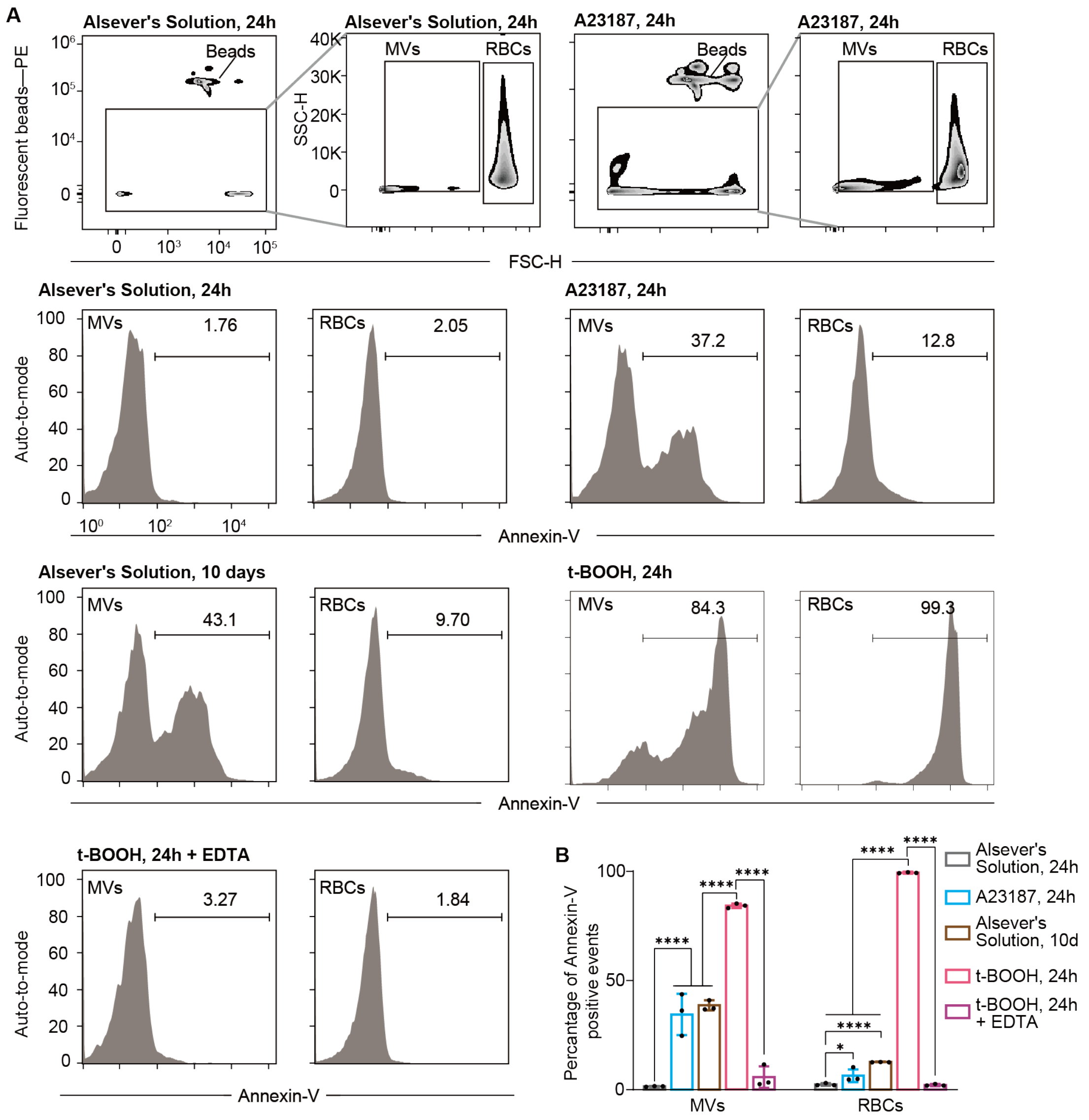

Figure S16. Flow cytometry analysis and Annexin V expression quantification in MVs and RBCs.

(**A**) Flow cytometry of MVs and RBCs for annexin V expression. MVs were inducted in RBCs treated with PBS, A23187, and t-BOOH for 24 hours or stored at 4°C for 10 days. MVs were separated from RBCs using 4 μm diameter PE-labeled microspheres. This method distinguishes PE-negative RBCs and MVs by their forward scatter height (FSH): the former have a larger FSH, while the latter has a smaller FSH, compared to microspheres. (**B**) Quantification of the percentage of annexin V positive RBCs and MVs, highlighting elevated annexin V expression in t-BOOH treated MVs. n = 3 per group. P-values were determined by one-way ANOVA with Fisher's LSD post-hoc test. *, $P < 0.05$; **, $P < 0.01$; ***, $P < 0.001$; ****, $P < 0.0001$.

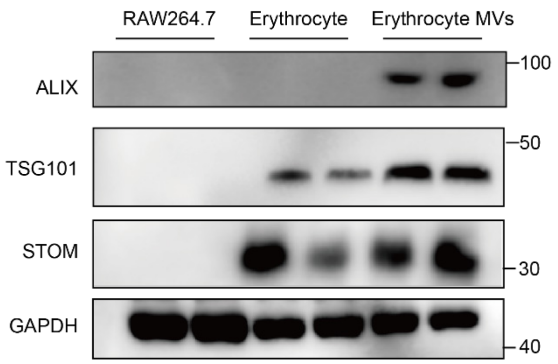

Figure S17. Characterization of the distinctive features of MVs compared to erythrocytes and RAW264.7 cells. Western blot analysis illustrates the detection of MV markers ALIX and TSG101 alongside the marker for erythrocytes-derived MVs, Stomatin (STOM), in comparison to GAPDH, serving as a loading control. The samples include MVs from erythrocytes, cell lysates from erythrocytes, or RAW264.7 cells.

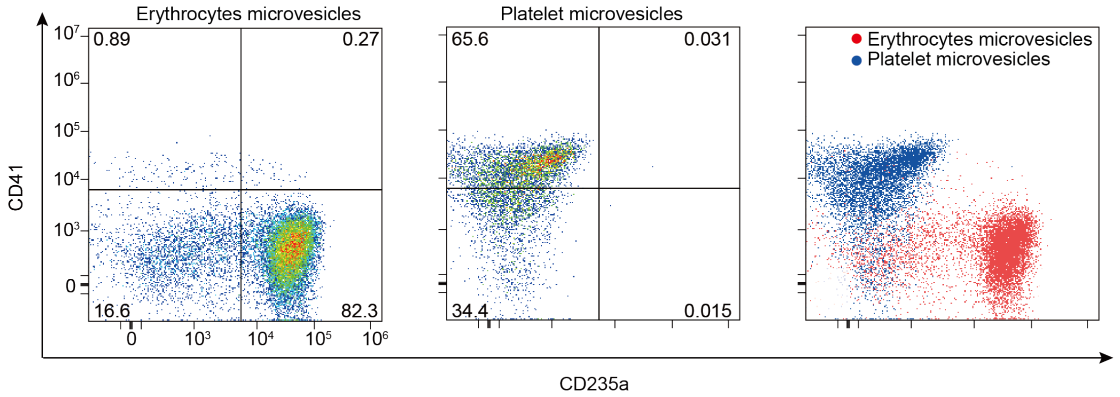

Figure S18. Purity assessment of erythrocyte-derived MVs via flow cytometry. MVs isolated from platelets were used as a control. Identification markers included the erythrocyte-specific antigen CD235a and the platelet-specific antigen CD41. Flow cytometric analysis demonstrated that the isolated erythrocyte MVs were virtually free of contamination from platelet-derived MVs.

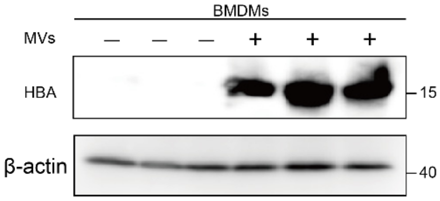

Figure S19. Western blot analysis of HBA relative to GAPDH in BMDMs untreated or incubated with 8 × 10^{11} erythrocyte-derived MVs for 24 h.

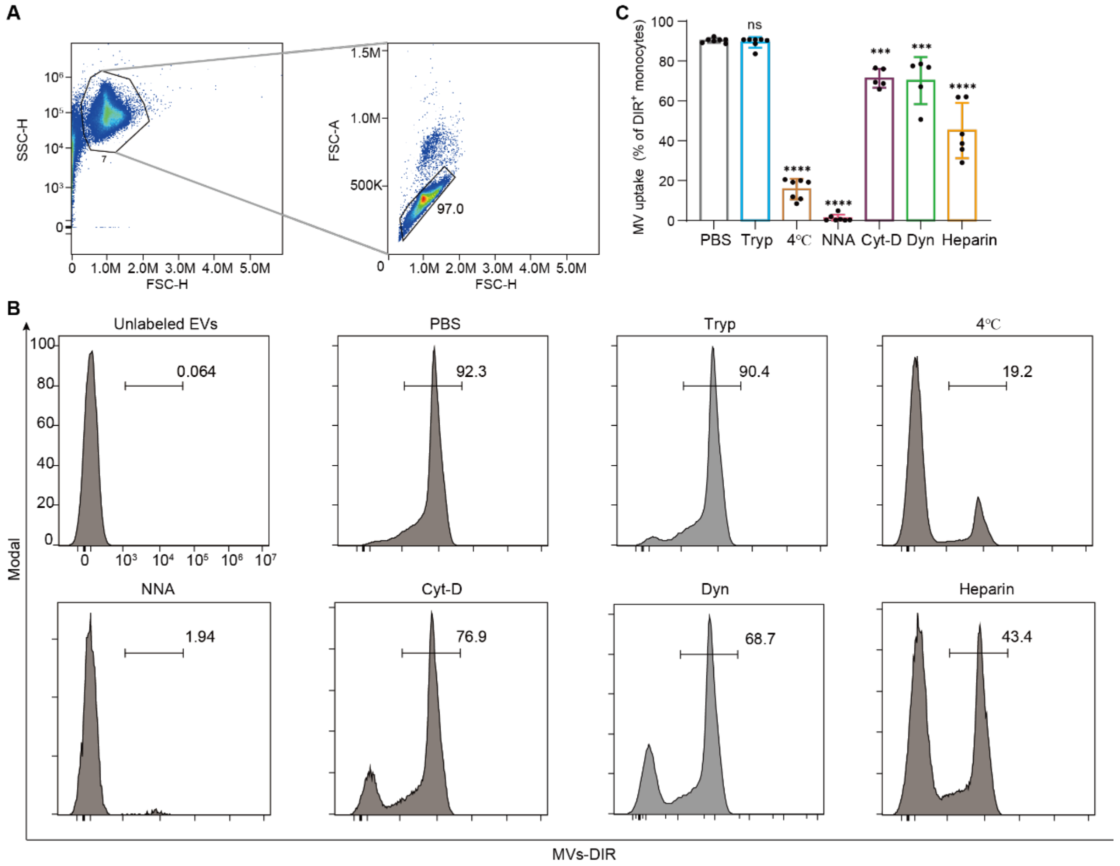

Figure S20: Flow cytometric analysis of bone marrow-derived macrophages (BMDMs) after co-incubation with erythrocyte microvesicles (MVs).

(**A**) Flow cytometry gating strategy was employed to identify BMDMs. (**B**) Representative peak profiles of fluorescently positive BMDMs uptaking DIR-prelabeled erythrocyte MVs. The numbers in the figure represent the proportion of DIR fluorescence-positive cells. (**C**) Quantification of fluorescence-positive cells (indicating successful uptake of erythrocyte MVs) by: (1) Trypsin treatment of cells post-MV-DiR incubation to remove any surface-bound MVs; (2) MV-cell co-incubation at 4°C or under energy depletion (NNA: NaF, NaN3, and antimycin A) to differentiate between active and passive uptake; (3) Inhibition of cytoskeleton rearrangement (Cyt-D: cytochalasin D) and dynein (Dyn: dynasore) to inhibit endocytic processes; (4) Heparin treatment for 24 hours to investigate the role of heparan sulfate proteoglycans. We compared the statistical differences between intervention conditions and the control PBS group. Bar charts are presented as mean ± SD. P-values were determined by one-way ANOVA with Fisher's LSD post-hoc test. *, $P < 0.05$; **, $P < 0.01$; ***, $P < 0.001$; ****, $P < 0.0001$.

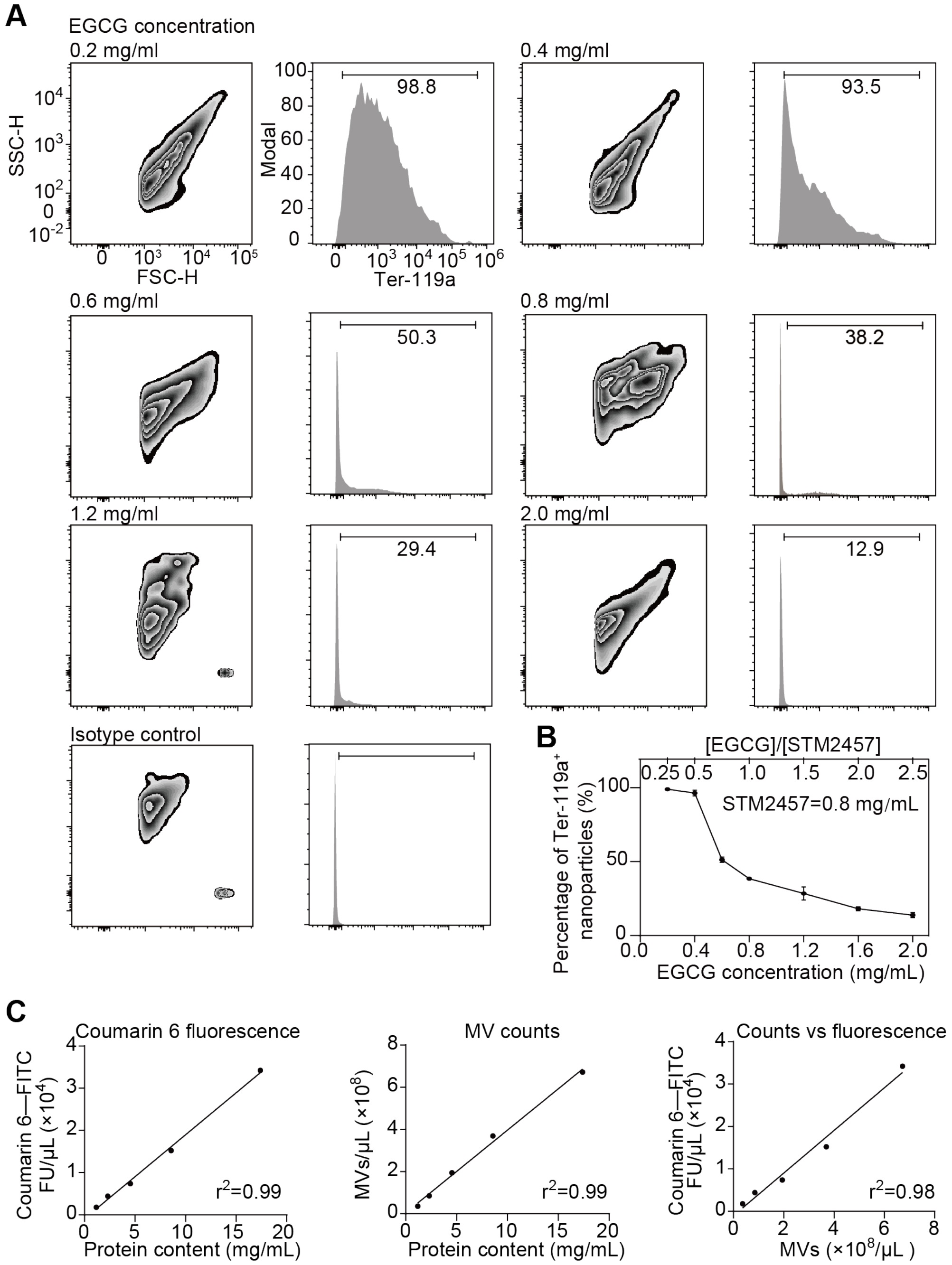

Figure S21: Flow cytometry and quantification of Ter-119a positive nanoparticles in drug-loaded STM@MV*nex-u*.

(**A**) Flow cytometry showing the proportion of Ter-119a positive nanoparticles in response to varying concentrations of EGCG. (**B**) Quantitative analysis of the percentage of Ter-119a positive nanoparticles, indicating the formation of MPN nanoparticles with MVs. n = 3 per group. (**C**) Linear correlation in serially diluted STM@MV*nex-u* between coumarin 6 fluorescence units (FU), MVs counts and total protein content of MVs as determined by BCA method, highlighting the measurable range of coumarin 6 fluorescence and consistency in drug-loading. P-values were determined by one-way ANOVA with Fisher's LSD post-hoc test. *, $P < 0.05$; **, $P < 0.01$; ***, $P < 0.001$; ****, $P < 0.0001$.

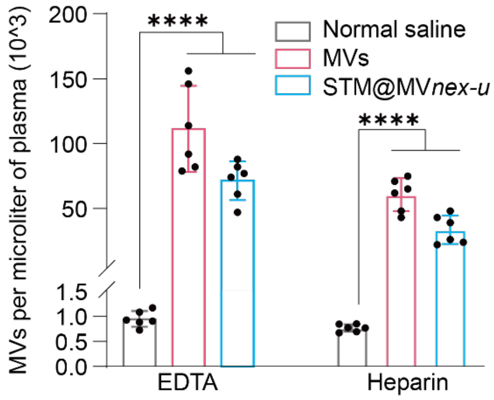

Figure S22. Quantification of MVs isolated from whole blood collected using different anticoagulants. The quantification was conducted utilizing DLS technique. Two hours post-injection of 2×10^{11} MVs, the proportion of endogenous MVs in the plasma was nearly negligible.

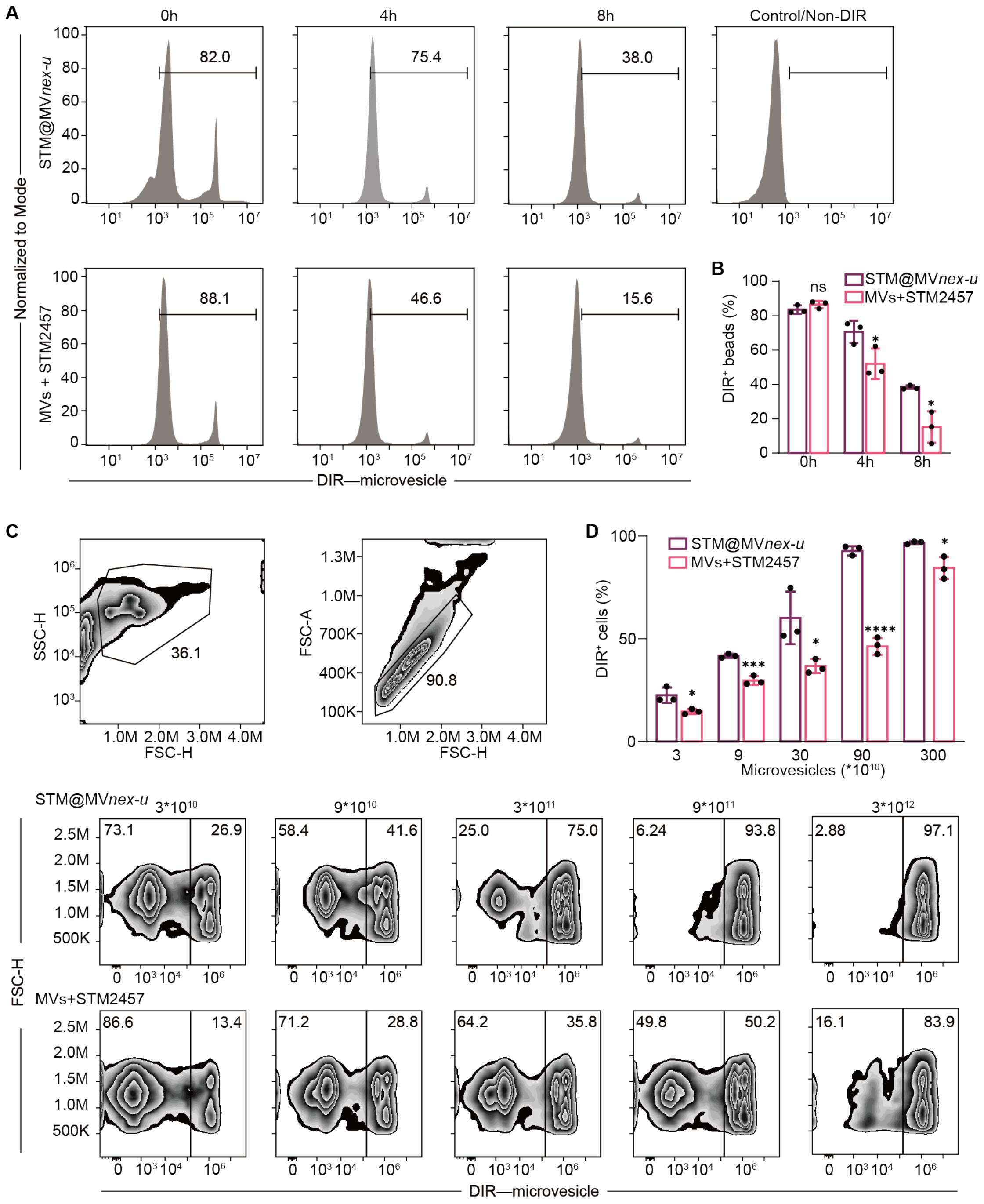

Figure S23. MVs dynamics in serum and cardiac tissue post-treatment

(**A**) Flow cytometric analysis of serum exosomes captured on gel beads after treatment of MVs and STM@MV*nex-u*. (**B**) Quantification of DIR fluorescence in gel beads post tail vein injection of MVs or STM@MV*nex-u*. n = 3 per group. (**C**) Flow cytometry of cardiac cells assessing DIR-positive cells. (**D**) Quantification of DIR-positive non-myocardial cells. n = 3 per group. P-values were determined by one-way ANOVA with Fisher's LSD post-hoc test.

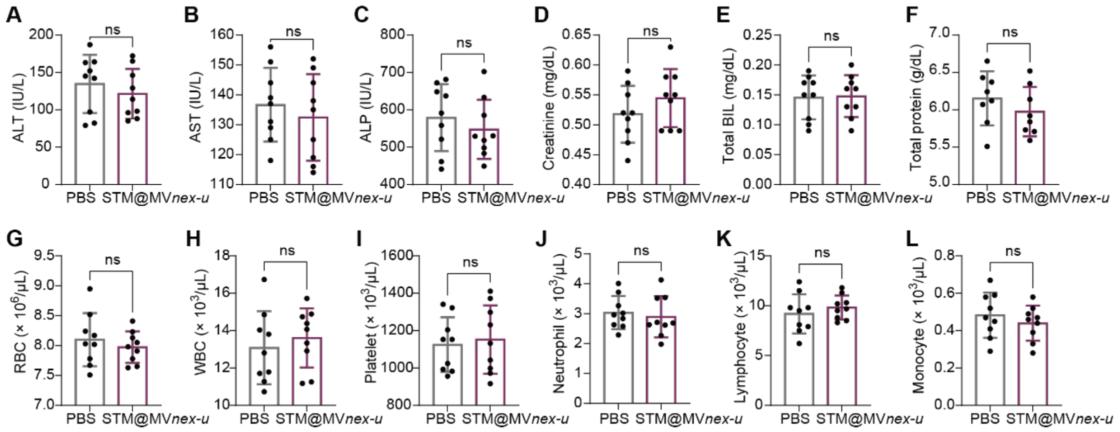

Figure S24. *In-vivo* toxicity of STM@MV*nex-u* one month after injection into mice's tail veins.

(**A**-**F**) Clinical chemistry analysis for constituents in blood and urine, including aminotransferase (ALT and AST), alkaline phosphatase (ALP), creatinine, total bilirubin (BIL), and total proteins. n = 9 per group. (**G**-**L**) Hematological analysis for STM@MV*nex-u* injected into mice's tail veins. The blood cell count levels at 29 days post-injection, including red blood cells (RBCs), white blood cells (WBCs), platelets, neutrophils, lymphocytes, and monocytes. n = 9 per group. P-values were determined by one-way ANOVA with Fisher's LSD post-hoc test. *, $P < 0.05$; **, $P < 0.01$; ***, $P < 0.001$; ****, $P < 0.0001$.

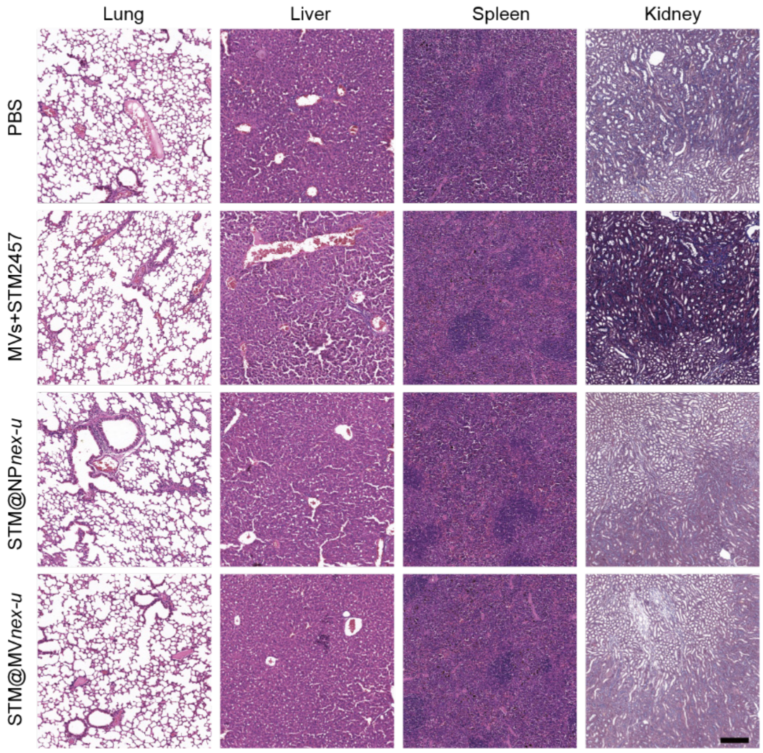

Fig. S25. H&E of mouse organs following different treatments in the Ang II-induced cardiac fibrosis and remodeling model

Mice received the same doses of formulations as in the efficacy study (Fig. 8). Organs of mice were analyzed by H&E staining. Representative of n = 6 biologically independent animals per group.

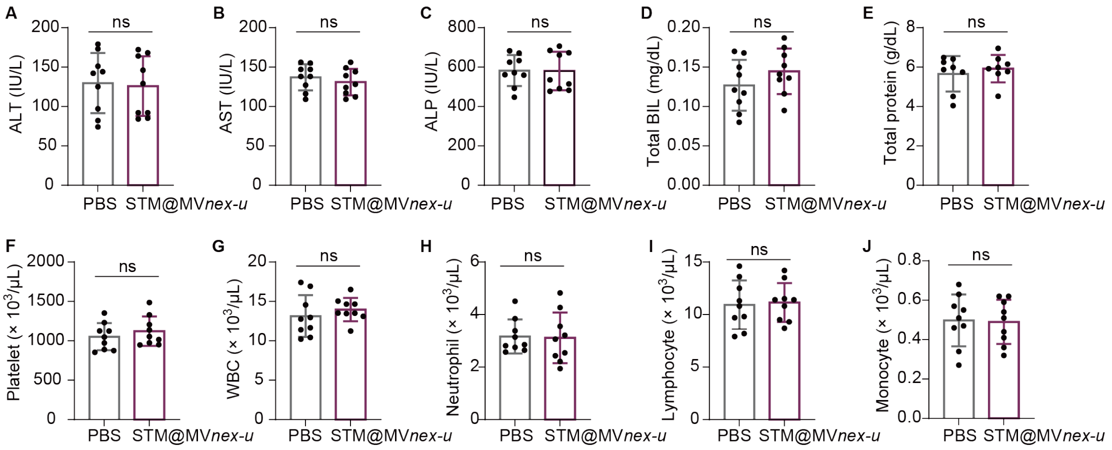

Figure S26. In vivo long-term toxicity assessment of STM@MV*nex-u* three months after injection in mice

(**A-E**) Clinical chemistry analysis for constituents in blood, including ALT, AST, ALP, total BIL, and total proteins. n = 9 per group. (**F-J**) Hematological analysis performed on mice injected STM@MV*nex-u* by tail veins. The blood cell count levels at 3 months post-injection, including platelets, WBCs, neutrophils, lymphocytes, and monocytes. n = 9 per group. P-values were determined by one-way ANOVA with Fisher's LSD post-hoc test. *, $P < 0.05$; **, $P < 0.01$; ***, $P < 0.001$; ****, $P < 0.0001$.

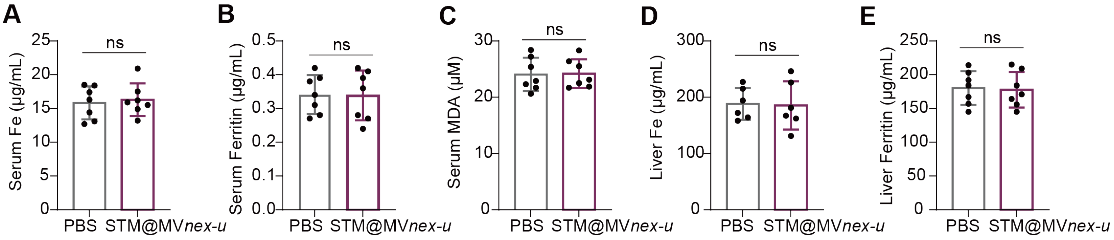

Figure 27. Iron and ferritin levels of plasma and liver in STM@MV*nex-u* treated mice three months after injection. Iron was quantified using a bathophenanthroline sulfonate (BPS) colorimetric assay, and ferritin was quantified via ELISA.

(**A**) Serum iron concentration. (**B**) Serum ferritin concentration. (**C**) Serum malondialdehyde (MDA) level. (**D**) Iron concentration in the liver. (**E**) Ferritin concentration in the liver. P-values were determined by one-way ANOVA with Fisher's LSD post-hoc test. *, $P < 0.05$; **, $P < 0.01$; ***, $P < 0.001$; ****, $P < 0.0001$.

Table S1. Types and proportions of conduction block

Classification	TCB (%)	PCB (%)
RBBB	29(43.26%)	9 (36%)
LBBB	7 (10.4%)	2 (8%)
LAFB	15 (22.4%)	5 (20%)
RBBB with LAFB	8 (11.9%)	4 (16%)
I° AVB	5 (7.5%)	2 (8%)
II° AVB	2 (3%)	2 (8%)
III° AVB	1 (1.5%)	1 (4%)
Total	67	25

RBBB, right bundle branch block; LBBB, left bundle branch block; LAFB, left anterior fascicular block; AVB, atrioventricular block.

Table S2. Analysis of risk factors for conduction block prognosis

Variable	Transient<br>n = 67	Persistent<br>n = 25	Sig
Age (years)	3.99±2.71	3.52±2.39	0.42
gender			0.237
Male	32（47.8%）	16（64%）	
Female	35（52.2%）	9（36%）	
PR in+K20:M38terval (ms)	126.51±15.67	131.16±19.48	0.407
P-R-T axis (°)	41.92±56.44	7.92±54.11	0.108
QRS (ms)	88.73±10.49	93.49±16.82	0.233
P wave width (ms)	88.54±13.62	92.41±12.57	0.337
P wave height (mV)	0.13±0.07	0.12±0.07	0.671
QT (ms)	327.61±36.65	327.92±38.45	0.981
QTC (ms)	416.24±22.56	419.71±20.62	0.721
RV5 (mV)	1.67±0.74	1.51±0.72	0.345
SV1 (mV)	0.88±0.64	0.76±0.51	0.249
RV5+SV1 (mV)	2.49±0.74	2.35±0.88	0.312
RV1 (mV)	0.84±0.57	0.56±0.31	0.023
SV5 (mV)	0.74±0.75	0.69±0.34	0.713
RV1+SV5 (mV)	1.45±0.79	1.22±0.47	0.071
Entrance of defect (mm)	6.74±2.69	6.09±2.41	0.464
Outlet of defect (mm)	4.21±2.14	4.08±1.29	0.828
Distance from AO (mm)	1.51±1.48	1.62±1.53	0.729
Clock bit	10.14±0.29	10.05±0.3	0.572
Conveyance sheath size(F)	6.24±1.37	6.39±1.27	0.654
Size of occluder (mm)	6.79±2.37	7.51±2.88	0.154
Defect type			0.468
Perimembrane	60（89.6%）	21（84%）	
Funnel	7（10.4%）	4（16%）	
PAH	11（16.4%）	7（28.0%）	0.232

Valve regurgitation			0.686
Aortic regurgitation	1（1.5%）	0（0.0%）	
Mitral regurgitation	8（11.9%）	2（8%）	
Tricuspid regurgitation	21（31.3%）	7（28%）	
LV enlargement	24（35.8%）	13（52%）	0.098
LA enlargement	41（61.2%）	14（56%）	0.537
Occluder type			0.13
Symmetric	47（70.1%）	22（88%）	
ADOII	13（19.4%）	4（16%）	
Residual shunt	2（3.0%）	1（4%）	0.684
Hemolysis	2（3.0%）	0（0.0%）	0.496

Table S3. Multivariable analysis of risk factors for persistent conduction block prognosis

Variable	OR	Sig.	Lower 95% CI	Upper 95%CI
Age	0.9	0.264	0.761	1.064
gender	1.428	0.474	0.548	3.72
Entrance of defect	1.031	0.699	0.896	1.187
Outlet of defect (mm)	1.001	0.997	0.779	1.289
Distance from AO (mm)	0.961	0.689	0.799	1.155
Conveyance sheath size (F)	0.952	0.739	0.71	1.276
Size of occluder (mm)	0.863	0.167	0.703	1.06
Defect type	1.351	0.71	0.256	7.128
PAH	1.113	0.526	0.812	1.524
Valve regurgitation	1.229	0.769	0.32	4.721
LV enlargement	0.452	0.079	0.186	1.096
LA enlargement	0.989	0.823	0.874	1.118
Occluder type	2.472	0.204	0.605	10.106
Residual shunt	1.369	0.838	0.106	17.636
Hemolysis	1.837	0.685	0.081	41.652

Table S4. Primers for PCR (qRT-PCR, MeRIP-PCR, CHIP-PCR)

Gene	Primer sequence Forward (5'-3')	Reverse (5'-3')
**Human**		
METTL3	TTGTCTCCAACCTTCCGTAGT	CCAGATCAGAGAGGTGGTGTAG
METTL14	AGTGCCGACAGCATTGGTG	GGAGCAGAGGTATCATAGGAAGC
METTL16	TTCTGTCAAGGTCGGACAATG	CAGCACCACGAATGTTATGGG
WTAP	CTTCCCAAGAAGGTTCGATTGA	TCAGACTCTCTTAGGCCAGTTAC
FTO	AACACCAGGCTCTTTACGGTC	TGTCCGTTGTAGGATGAACCC
ALKBH5	CGGCGAAGGCTACACTTACG	CCACCAGCTTTTGGATCACCA
YTHDF1	CAAGCACACAACCTCCATCTTCG	GTAAGAAACTGGTTCGCCCTCAT
YTHDF2	TAGCCAGCTACAAGCACACCAC	CAACCGTTGCTGCAGTCTGTGT
YTHDF3	GCTACTTTCAAGCATACCACCTC	ACAGGACATCTTCATACGGTTATTG
YTHDC1	TCAGGAGTTCGCCGAGATGTGT	AGGATGGTGTGGAGGTTGTTCC
IL-1β	TGGCTTATTACAGTGGCAATGAG	GTAGTGGTGGTCGGAGATTCG
TNF-α	GTCTGGGCAGGTCTACTTTGG	GAGGTTGAGGGTGTCTGAAGG
IL-10	GTTGTTAAAGGAGTCCTTGCTG	TTCACAGGGAAGAAATCGATGA
CD163	CATTATGTCCTTCAGAGCAAGTG	AGCGACCTCCTCCATTTACC
CXCR1	TCCTTTTCCGCCAGGCTTACCA	GGCACGATGAAGCCAAAGGTGT
CXCR2	TCCGTCACTGATGTCTACCTGC	TCCTTCAGGAGTGAGACCACCT
CXCR4	CTCCTCTTTGTCATCACGCTTCC	GGATGAGGACACTGCTGTAGAG
CCR2	CCACATCTCGTTCTCGGTTTATC	CAGGGAGCACCGTAATCATAATC
CCR7	CAACATCACCAGTAGCACCTGTG	TGCGGAACTTGACGCCGATGAA
ITGA4	GCATACAGGTGTCCAGCAGAGA	AGGACCAAGGTGGTAAGCAGCT
ITGB2	TGCGTCCTCTCTCAGGAGTG	GGTCCATGATGTCGTCAGCC
TGF-β1	ACTGGAGTTGTACGGCAGTG	GGATCCACTTCCAACCCAGG
bFGF	AGCGGCTGTACTGCAAAAACGG	CCTTTGATAGACACAACTCCTCTC
IL-6	AGCCACTCACCTCTTCAGAAC	GCAAGTCTCCTCATTGAATCCAG
VEGF	GAGGAGCAGTTACGGTCTGTG	TCCTTTCCTTAGCTGACACTTGT
MyD88	GGCTGCTCTCAACATGCGA	CTGTGTCCGCACGTTCAAGA
Traf6	CAATGCCAGCGTCCCTTCCAAA	CCAAAGGACAGTTCTGGTCATGG
GAPDH	ACCACCCTGTTGCTGTAGCCAA	ACCACCCTGTTGCTGTAGCCAA
β-actin	CACCATTGGCAATGAGCGGTTC	AGGTCTTTGCGGATGTCCACGT
**Mouse**		
METTL3	CAGTGCTACAGGATGACGGCTT	CCGTCCTAATGATGCGCTGCAG
METTL14	AGAGTGCGGATAGCATTGGTGC	CTCCTTCATCCAGACACTTCCG
SPP1	GATAGCTTGGCTTATGGACTGAGG	GACTCCTTAGACTCACCGCTCTT
YM1	TACTCACTTCCACAGGAGCAGG	CTCCAGTGTAGCCATCCTTAGG
Arg1	CATTGGCTTGCGAGACGTAGAC	GCTGAAGGTCTCTTCCATCACC
IL-6	TACCACTTCACAAGTCGGAGGC	CTGCAAGTGCATCATCGTTGTTC
MMP9	GCAATGTGGATGTTTTTGATGCTATT	CCTGTAATGGGCTTCCTCTATGATT
TGF-β1	CCTATATTTGGAGCCTGGACACAC	GCTTGCGACCCACGTAGTAGA
IL-10	GGACAACATACTGCTAAAGGACTCCT	GCCTGGGGCATCACTTCTAC
NOS2	GAGACAGGGAAGTCTGAAGCAC	CCAGCAGTAGTTGCTCCTCTTC

	TNF-a	ATGGCCTCCCTCTGATCAGTT	TCTTTGAGATCCATGCCGTTG
	MyD88	ACCTGTGTCTGGTCCATTGCCA	GCTGAGTGCAAACTTGGTCTGG
	Traf6	TTTCCCTGACGGTAAAGTGCCC	ACCTGGCACTTCTGGAAAGGAC
	CXCR2	CTCTATTCTGCCAGATGCTGTCC	ACAAGGCTCAGCAGAGTCACCA
	CD163	GGCTAGACGAAGTCATCTGCAC	CTTCGTTGGTCAGCCTCAGAGA
**Merip of MyD88**			
S1	40-132	GGTTGCCTGCCATGTCT	GCCTCACTCCCACGTTAAG
S2	332-431	GCTGCTGGCCTTGTTAGA	CTCCTGGTTCTGCTGCTTAC
S3	466-564	GCAGTGTCCCACAAACAAAG	GCAGTAGCAGATAAAGGCA
S4	541-645	TCGATGCCTTTATCTGCTACTG	GGTCGGACACACACAACTTA
S5	803-903	ACAGAAGCGACTGATTCCTATT	TGGTGCAAGGGTTGGTATAG
S6	944-1041	TCCCTGCCCTGAAGATGA	GGAGTCAAAGATGTAGACAGGAC
S7	1017-1148	CCGTCCTGTCTACATCTTTGAC	GAGGAGGCATGTGTGTACTG
S8	1195-1314	CAAGGATAGCTGAGTGGAAGAG	ACCTCCCAAATGCTGAAACTA
**Merip of TGF-β1**			
S1	251-447	GAGCTGGTTGAGAGAAGAGGAA	GAGGGCTGGTCCGGAAT
S2	712-833	CTGCTGCTTTCTCCCTCAA	GGGAACCCTCGGCAAAG
S3	909-1041	ACTCCCGTGGCTTCTAGT	CCTTAGTTTGGACAGGATCTGG
S4	1699-1790	AGAGCCCTGGATACCAACTA	CAACCCAGGTCCTTCCTAAAG
**Merip of CXCR2**			
S1	447-551	CTGTCTGGGCTGCATCTAAA	GTAGCAGAACACTGCTGTAGAA
S2	1043-1151	ATTCTTGGCTTCCTCCACAG	TGCTGACAAGGCCATAAGTAG
S3	1195-1320	GTCTTCAGCAAACACCTCTACT	GGGCTGCATCAATTCAAATACC
S4	1537-1624	AAAGGAAGAGGTGGAGGAGA	TGGTACTCGTCTACTGGTAAGG
S5	2180-2316	GATCCCTGTGTAGTAGTGTGAATG	TTGTCGAGGTGCTAGGATTTG
S6	2639-2736	CCAGAGGAGGAACATCAGTATG	TACAGCTAGAGCCACAAGTTC
**CHIP of METTL3**			
S1	-2420~-2411	AAGTGTTCTGTCCATTATAGCA	CTGAGTTCCAGAACCAAGATT
S2	-1866~-1857	GGGACAGTGAACTCACTCAAG	GGGACAGTGAACTCACTCAAG
S3	-922~-912	CCACACAGCTTTGCCTTAAAT	GGAGGTAGCTCAGTAAAGTGTT
S4	-578~-569	CATCTCTCCACTCATCCTGAAC	CACAATCCTCCTGCCTCAATA
**CHIP of METTL14**			
S1	-2088~-2079	CACCTTCTGATGTTGGGAATTAAC	TCCAGCTGCCTATTGCATAC
S2	-1233~-1224	TCTAGTCCCGGCAGTCATAA	AGAGCAAATTTCAGGGCAGC
S3	-53~-44	GGCGATCCAGTCCTTCACAA	CTGAACAGGAAGTCCCGCC
S4	-14~-5	ACTTCCTGTTCAGGCATAGC	GCAAATCCACCTGCCAAAC
